# Comparative efficacy of different antihypertensive drug classes for stroke prevention: A network meta-analysis of randomized controlled trials

**DOI:** 10.1371/journal.pone.0313309

**Published:** 2025-02-21

**Authors:** Ding Yu, Jun-xia Li, Yuan Cheng, Han-dong Wang, Xin-di Ma, Tao Ding, Zhong-ning Zhu

**Affiliations:** 1 Heart Center, The First Hospital of Hebei Medical University, Shijiazhuang, China; 2 Department of Pharmacology, Hebei Medical University, Shijiazhuang, China; 3 Department of Pathology, Hebei University of Chinese Medicine, Luquan, Shijiazhuang, China; 4 Department of General Surgery, The Fourth Hospital of Hebei Medical University, Shijiazhuang, China; 5 Undergraduate of Clinical Medicine, Hebei Medical University, Shijiazhuang, China; Tehran University of Medical Sciences, ISLAMIC REPUBLIC OF IRAN

## Abstract

**Objective:**

The study aimed to compare the effectiveness of various antihypertensive drugs in preventing strokes in hypertensive patients.

**Methods:**

We conducted a comprehensive search of PubMed, Embase, the Cochrane Library, and ClinicalTrials.gov to identify randomized controlled trials (RCTs) investigating the efficacy of antihypertensive drugs in stroke prevention from inception until April 2023. A network meta-analysis in a Bayesian framework was performed using the random-effects model.

**Results:**

This study included 88 RCTs involving 487,076 patients to investigate the effects of antihypertensive drugs in preventing stroke. Among these trials, 58 RCTs specifically focused on comparing the impact of such drugs on hypertensive subjects. In overall population, Angiotensin-converting enzyme inhibitor (ACEIs), Angiotensin receptor blockers (ARBs), Calcium channel blockers (CCBs), and Diuretics (DIs) demonstrated superiority over placebo in in reducing stroke, all-cause mortality, and cardiovascular mortality. CCBs and DIs outperformed β adrenergic receptor blockers (BBs), ACEIs, and ARBs in stroke reduction. However, when focusing on hypertensive patients, ACEIs, CCBs, and DIs proved superior to placebo in reducing stroke, all-cause mortality, and cardiovascular mortality. ARBs reduced stroke and all-cause mortality but lacked efficacy in reducing cardiovascular mortality. Of the various CCB subclasses, only the Dihydropyridines displayed efficacy in preventing stroke, all-cause mortality, and cardiovascular mortality. Among diuretic subclasses, thiazide-type DIs exhibited no efficacy in preventing all-cause mortality. ACEIs+CCBs were more effective than ACEIs or ARBs monotherapy in reducing stroke, more effective than ACEIs, ARBs, CCBs, or DIs monotherapy in reducing all-cause mortality, and more effective than ARBs in reducing cardiovascular mortality.

**Conclusion:**

These findings suggest that ACEIs, dihydropyridine CCBs, and thiazide-like diuretics may provide superior prevention against stroke, all-cause mortality, and cardiovascular mortality in hypertensive patients. Combinations of ACEIs and CCBs may provide enhanced protection of stroke than ACEIs or ARBs monotherapy.

## Introduction

Despite the considerable improvements in diagnosis and treatment, stroke remains the second leading cause of death for people aged above 60 years and the fifth cause for those between 15 and 59 years old [1]. Hypertension poses a high attributable risk for stroke (25%–50%) [2], and is considered as the primary cause of stroke. Previous clinical trials have confirmed that antihypertensive therapy is most effective in controlling hypertension and preventing subsequent stroke events [3].

The current hypertension guidelines recommend the use of angiotensin-converting enzyme inhibitors (ACEIs), angiotensin-II receptor blockers (ARBs), calcium-channel blockers (CCBs), diuretics (DIs), and β blockers (BBs) alone or in combination as first-line agents for hypertension and its complications, including stroke [4,5]. However, there is no consensus on the most appropriate treatment for stroke prevention. First, despite several published systematic reviews and pairwise meta-analyses, the lack of direct, head-to-head comparisons between antihypertensive medication classes limits the identification of the most effective and safe treatment strategy for stroke prevention. Second, a network meta-analysis of 93 studies by Zhong et al. revealed that DI, CCB, and ARB, either alone or in combination, could be considered as first-line treatments for stroke prevention in the general population [6]. Another network meta-analysis by Wang et al. showed that CCB and DI had the highest probability of reducing stroke incidence in cardiovascular disease patients [7]. However, it remains unclear which antihypertensive drugs are most effective for preventing stroke in hypertensive patients. Third, all major systematic reviews have not considered the differences among the sub-classes of DIs and CCBs. For example, diuretics can either be thiazide-type or thiazide-like subclass, while CCBs can be categorized as dihydropyridine and non-dihydropyridine subclass. Therefore, the primary aim of this study is to conduct a comprehensive network meta-analysis of all relevant RCTs to determine the efficacy of different antihypertensive drugs in hypertensive patients, hoping to provide a foundation for the selection of antihypertensive drugs in clinical practice for stroke prevention in hypertensive patients.

## Methods

The study was conducted in accordance with the PRISMA guidance [8]. The PRISMA checklist was reported in “S1 PRISMA NMA checklist”.

### Literature search strategy

We searched the databases including Pubmed, Embase, the Cochrane Library, and Clinical Trials.gov (Last search was updated on April, 2023). We used search terms (‘‘stroke” OR ‘‘ischemic stroke” OR ‘‘cerebrovascular accident” OR ‘‘CVA”) AND (“antihypertensive agents” OR “blood pressure-lowering” OR “blood pressure lowering” OR “blood-pressure lowering” OR “diuretics” OR “Angiotensin-converting enzyme inhibitors” OR “Angiotensin receptor antagonists” OR “calcium channel blockers” OR “beta blocker” OR “ACEI” OR “ARB” OR “CCB”). Additionally, we conducted a manual search of the bibliographies of all the included articles and related reviews to identify any other potentially eligible trials. The search strategy used for each database are present in S1 Table.

### Study selection criteria

Studies were eligible if they fulfilled all the following: 1) they were randomized controlled trials; 2) they compared an antihypertensive agent (alone or in combination) against a second antihypertensive agent or combination therapy, placebo, or control; 3) they provided information on stroke, all-cause mortality, or cardiovascular mortality events. Trials with a follow-up <6 months were excluded. For duplicated trials, the longest follow-up trials were included.

### Outcomes assessments

Our outcomes of interest were stroke (including fatal and non-fatal events), all-cause mortality, and cardiovascular mortality.

### Data extraction

Two independent investigators reviewed the publications and extracted the data. Controversial data would be reviewed by a third investigator. We extracted the following data: first author, publication year, sample size, treatment class, duration interventions, and outcomes of interest.

### Study quality assessment

We assessed the risk of bias of the included randomized trials using a revised version of the Cochrane’s ’Risk of bias’ tool (RoB 2.0). Each trial was judged to be at ’low risk of bias’, ’some concerns’ or ’high risk of bias’ [9].

### Statistical analysis

We conducted a Bayesian network meta-analysis using Markov chain Monte Carlo methods in JAGS and the GeMTC package (version 0.8–2) in R(version 4.2.3) software [10] and the web application “MetaInsight V4.1.0” (https://crsu.shinyapps.io/MetaInsight/). We calculated treatment estimates as relative risks (RRs) with their 95% credible intervals (CrIs). Model fit was assessed according to the deviance information criteria (DIC). Three different models were run for each outcome: fixed-effect model, random-effects model, and random-effects inconsistency model. The model with the lowest DIC was considered the model providing the best fit to the data. We set 20,000 simulations for each chain as the “burn-in” period, yielding 50,000 iterations to obtain effect estimates, when 4 Markov chains run simultaneously. The assumption of inconsistency between direct and indirect evidence was assessed globally (by fitting both an inconsistency model and a consistency model) and locally (by carrying out the sidesplitting method). We plotted comparison-adjusted funnel plots for each outcome to make visual assessments of possible publication bias. The overall quality of the network meta-analysis was evaluated using the web application CINeMA (Confidence in Network Meta-Analysis). We rated the certainty of each outcome as ‘high’, ‘moderate’, ‘low’ or ‘very low’ [11].

We performed subgroup analyses to investigate whether the treatment effect varies across different subgroups. The diuretic class was categorized into thiazide-type diuretics and thiazide-like diuretics, while the CCB class was subdivided into dihydropyridine, Verapamil, and Diltiazem CCBs. We then reanalyzed the data to assess the effects.

## Results

### Search results and study characteristics

A total of 1347 records were identified from the initial database search. A total of 1223 records were excluded for various reasons based on the titles and abstracts screen (reviews, letters, animal studies, not RCTs, or irrelevant to the analysis). The full texts of the remaining 124 studies were reviewed in detail. Then, 29 articles were excluded for the following reasons: study protocol, non-reporting of the outcomes of interest, non-reporting of comparison of interests. Finally, 88 RCTs with 487,076 patients were included in the network meta-analysis. Of the 88 studies, 58 trials (305,171 patients) compared the effects of antihypertensive drugs for patients with hypertension.

**Fig 1:**
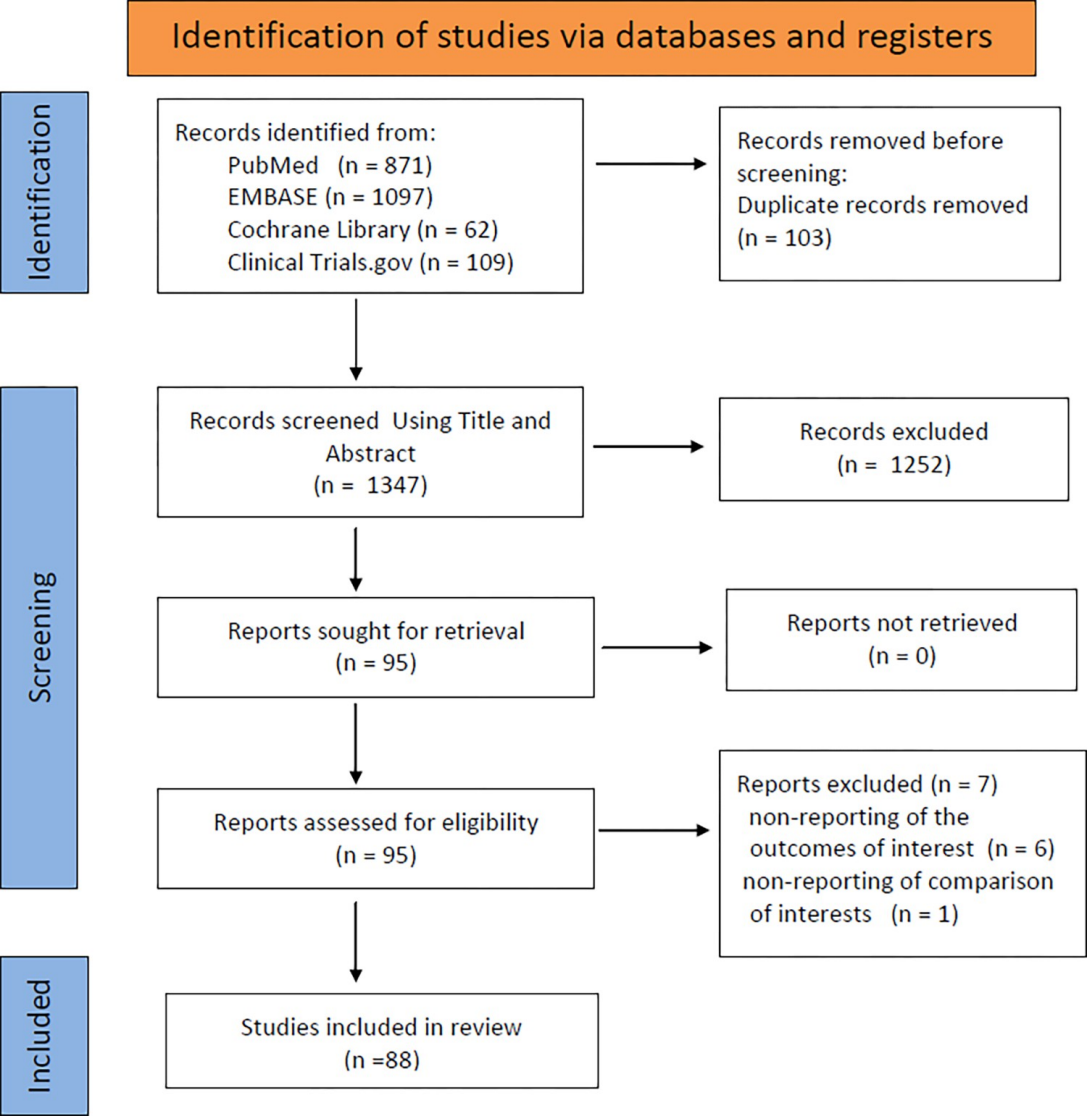
Flowchart of selecting process for meta-analysis.

**Fig 2:**
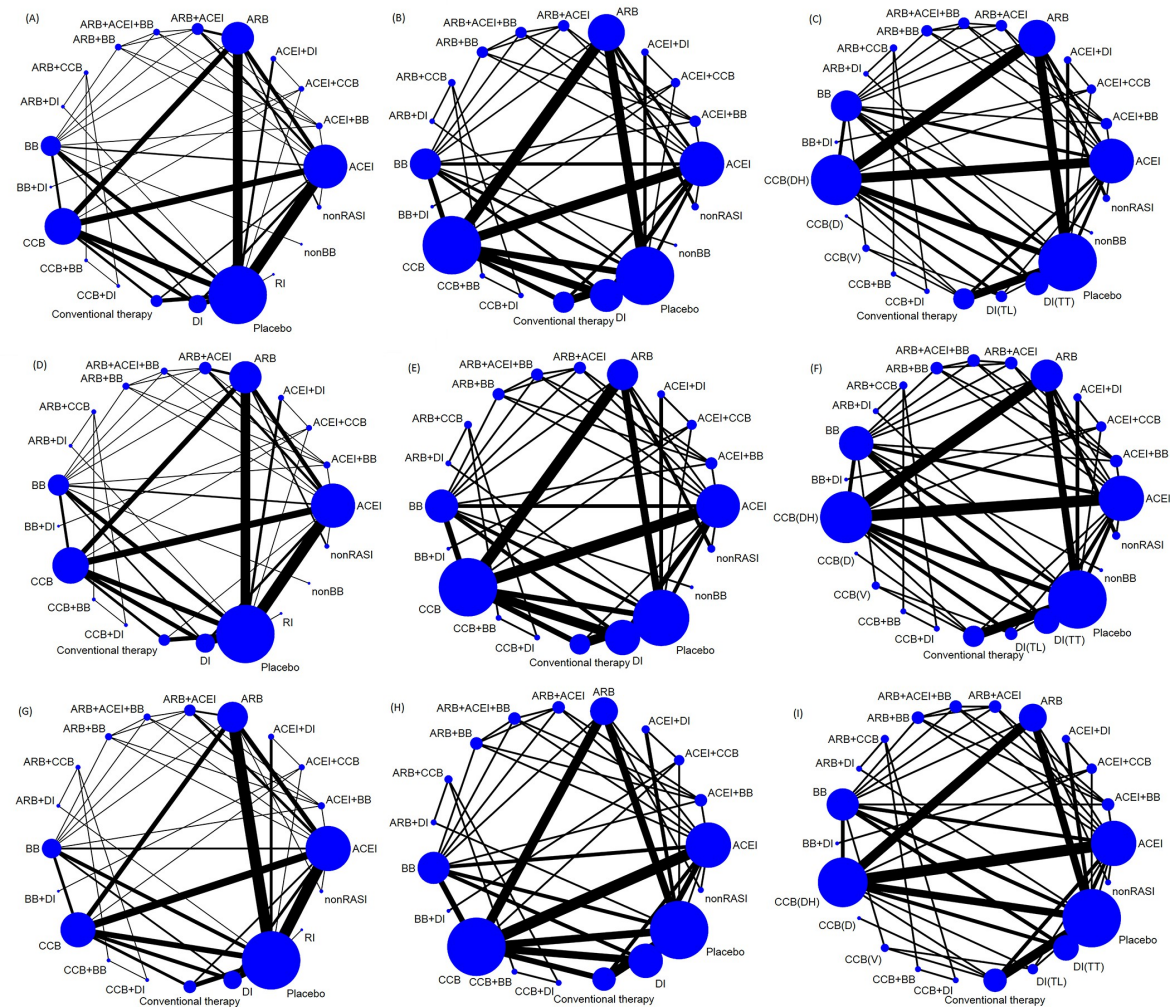
Network structure diagrams. (A) stroke in the overall population, (B) stroke in hypertensive patients, (C) subgroup analysis of stroke in hypertensive patients, (D) all-cause mortality in the overall population, (E) all-cause mortality in hypertensive patients, (F) subgroup analysis of all-cause mortality in hypertensive patients, (G) cardiovascular mortality in the overall population, (H) cardiovascular mortality in hypertensive patients, (I) subgroup analysis of cardiovascular mortality in hypertensive patients. Abbreviations: ARB, angiotensin receptor blockers; DI, Diuretics; DI(TL), thiazide-like diuretics; DI(TT), thiazide-type diuretics; CCB, calcium channel blockers; CCB(DH), dihydropyridine calcium channel blockers; CCB(D), calcium channel blockers (diltiazem); CCB(V), calcium channel blockers (verapamil); ACEI, angiotensin-converting enzyme inhibitors; BB, βadrenergic receptor blockers; nonRASI, non-renin-angiotensin system (RAS) inhibitors; RI, renin inhibitors.

The selection process is shown in Fig 1. The main characteristics of the included studies are shown in Table 1. The quality appraisals of the 88 RCTs are shown in S1 and S2 Figs. The network structure diagram is shown in Fig 2. Network structure diagrams are applied to display the direct association between different treatment regimens and the thicknesses of the lines provide a measure of the number of direct comparisons between two regimens.

**Table 1:** Characteristics of the trials included in the meta-analysis.

Author	Study	Disease	Treatment class	Treatment drugs	Duration	Age	N	All-cause mortality		CV mortality		stroke	
								n	%	n	%	n	%
Ruggenenti 2021 [12]	ARCADIA NCT00985322	Hemodialysis +HTN+/or LVH	ACEI non-RASI	Ramipril non-RAS inhibition	33m	64±12 62±14	140	26	18.6	11	7.9	4	2.9
							129	30	23.3	17	13.2	2	1.6
Lonn 2016 [13]	HOPE-3 NCT00468923	HTN at intermediate risk without CVD	ARB+DI Placebo	Candesartan+HCTZ Placebo	5.6y	65.7±6.4 65.8±6.4	6359	342	5.4	155	2.4	75	1.2
							6349	349	5.5	170	2.7	94	1.5
Sakata 2015 [14]	SUPPORT NCT00417222	HTN+CHF	ARB+ACEI ACEI ARB+BB BB ARB+ACEI+BB ACEI+BB	Olmesartan+ACEI ACEI Olmesartan+BB BB Olmesartan+ACEI+BB ACEI+BB	4.4y	66	170	30	17.6	14	8.2	14	8.2
							148	19	12.8	7	4.7	10	6.8
							106	10	9.4	7	6.6	7	6.6
							104	23	22.1	9	8.7	4	3.8
							299	58	19.4	27	9.0	13	4.3
							312	42	13.5	22	7.1	12	3.8
Ogihara 2014 [15]	COLM	Elderly HTN (65-85y)	ARB+ CCB(DH) ARB+DI	Olmesartan/Amlodipine or Azelnidipine Olmesartan/ Diuretics	3.3y	73.6±5.3 73.6±5.4	2568	64	2.5	13	0.5	63	2.5
							2573	76	3.0	18	0.7	66	2.6
Fried 2013 [16]	VA EPHRON-D NCT00555217	T2D+Nephropathy	ARB+ACEI ARB	Losartan+Lisinopril Losartan+Placebo	2.2y	64.5±7.9 64.7±7.7	724	63	8.7			18	2.5
							724	60	8.3			18	2.5
Parving 2012 [17]	ALTITUDE NCT00549757	T2D+CKD+/or CVD	RI Placebo	Aliskiren placebo	32.9m	64.6±9.6 64.4±9.9	4274	376	8.8	246	5.8	147	3.4
							4287	358	8.4	215	5.0	122	2.8
Asayama 2012 [18]	HOMED-BP UMIN C000000137	mild-to-moderate HTN >40y	ACEI ARB CCB(DH)	ACEIs ARBs CCBs	5.3y	59.8±10.0 59.5±10.1 59.5±10.1	1172	17	1.5	2	0.2	11	0.9
							1175	16	1.4	2	0.2	9	0.8
							1171	25	2.1	4	0.3	16	1.4
Muramatsu 2012 [19]	NHS NCT00129233	HTN+ T2D/IGT	ARB CCB(DH)	Valsartan Amlodipine	3.2y	63±8 63±8	575	22	3.8	4	0.7	13	2.3
							575	16	2.8	4	0.7	16	2.8
Tillin 2011 [20] Sjølie2008 [21]	DIRECT-Protect 2 NCT00252694	T2D+Retinopathy	ARB Placebo	Candesartan Placebo	4.7y	57±8 57±8	951	37	3.9	18	1.9	16	1.7
							954	35	3.7	25	2.6	15	1.6
		T2D+Retinopathy+HTN	ARB Placebo	Candesartan Placebo	4.7y	56.9±7.6 56.8±7.9	588	22	3.7	12	2.0	10	1.7
							592	33	5.6	21	3.5	12	2.0
Sandset 2011 [22]	SCAST NCT00120003	Acute stroke +HTN	ARB Placebo	Candesartan Placebo	6m	70.8±11.2 71.0±11.0	1017	84	8.3	63	6.2	69	6.8
							1012	78	7.7	60	5.9	59	5.8
Matsuzaki 2011 [23]	COPE NCT00135551 UMIN000001152	HTN (40-85y)	ARB+ CCB(DH) CCB(DH)+BB CCB(DH)+DI	Benidipine+ARB Benidipine+BB Benidipine+thiazide	3.6y	63.0±10.6 63.2±10.8 63.1±10.8	1110	25	2.3	21	1.9	17	1.5
							1089	23	2.1	19	1.7	27	2.5
							1094	23	2.1	17	1.6	12	1.1
ACTIVE I 2011 [24]	ACTIVE I NCT00249795	Atrial fibrillation	ARB Placebo	Irbesartan Placebo	4.1y	69.5±7.9 64.7±7.7	4518	949	21.0	666	14.7	379	8.4
							4498	929	20.7	646	14.4	411	9.1
Haller 2011 [25] Menne 2012 [26]	ROADMAP NCT00185159	T2D	ARB Placebo	Olmesartan Placebo	3.2y	57.7±8.8 57.8±8.6	2232	26	1.2	15	0.7	7	0.3
							2215	15	0.7	3	0.1	19	0.9
		HTN+ T2D	ARB Placebo	Olmesartan Placebo	3.2y	58.0±8.7 58.2±8.5	2043	25	1.2	14	0.7	7	0.3
							1977	14	0.7	3	0.2	19	1.0
Brouwers 2011 [27]	PREVEND IT	Microalbuminuria	ACEI Placebo	Fosinopril Placebo	9.5y	51.1±12.2 51.5±11.4	431	35	8.1	9	2.1	12	2.8
							433	32	7.4	12	2.8	19	4.4
Thijs 2010-a [28] Thijs 2010-b	Syst-Eur NCT00122811	HTN >60y	CCB(DH)+ACEI Placebo	Nitrendipine+Enalapril Placebo	2y	≥60	515	22	4.3	14	2.7	10	1.9
							559	31	5.5	19	3.4	20	3.6
		HTN >60y	CCB(DH)+ACEI CCB(DH)	Nitrendipine+Enalapril Nitrendipine	2y	≥60	871	49	5.6	32	3.7	18	2.1
							1552	141	9.1	77	5.0	52	3.4
Narumi 2010 [29]	VART UMINC000000074	HTN	ARB CCB(DH)	Valsartan Amlodipine	3.4y	60±12 60±11	510	2	0.4			10	2.0
							511	3	0.6			10	2.0
NAVIGATOR 2010 [30]	NAVIGATOR NCT00097786	Impaired glucose tolerance	ARB Placebo	Valsartan Placebo	6.5y	63.7±6.8 63.8±6.8	4631	295	6.4	128	2.8	105	2.3
							4675	327	7.0	116	2.5	132	2.8
Kasanuki 2009 [31]	HIJ-CREATE UMIN000000790	HTN+ CAD	ARB non-RASI	Candesartan non-ARB	4.2y	64.5±9.4 65.0±8.9	1024	69	6.7	28	2.7	45	4.4
							1025	59	5.8	25	2.4	49	4.8
Liu 2009 [32]	PATS	History of stroke or TIA±HTN	DI(TL) Placebo	Indapamide Placebo	2y	60.1±8.3 60.4±8.5	2840	145	5.1	86	3.0	159	5.6
							2825	161	5.7	102	3.6	219	7.8
		History of stroke or TIA+HTN	DI(TL) Placebo	Indapamide Placebo	2y	60.1±8.3 60.4±8.5	2397					146	6.1
							2355					191	8.1
Yusuf 2008 [33]	PRoFESS NCT00153062	Ischemic stroke	ARB Placebo	Telmisartan Placebo	2.5y	66.1±8.6 66.2±8.6	10146	755	7.4	223	2.2	880	8.7
							10186	740	7.3	263	2.6	934	9.2
		Ischemic stroke+HTN	ARB Placebo	Telmisartan Placebo	2.5y	66.1±8.6 66.2±8.6	3322					334	10.1
							3375					345	10.2
Yusuf 2008 [34]	TRANSCEND NCT00153101	CVD/ diabetes ACEI intolerance	ARB Placebo	Telmisartan Placebo	56m	66.9±7.3 66.9±7.4	2954	364	12.3	227	7.7	112	3.8
							2972	349	11.7	223	7.5	136	4.6
Ogihara 2008 [35]	CASE-J NCT00125463	HTN	ARB CCB(DH)	Candesartan Amlodipine	3.2y	63.8±10.5 63.9±10.6	2354	73	3.1			60	2.5
							2349	86	3.7			47	2.0
Massie 2008 [36]	I-PRESERVE NCT00095238	HF >60y	ARB Placebo	Irbesartan Placebo	49.5m	72±7 72±7	2067	445	21.5	311	15.0	68	3.3
							2061	436	21.2	302	14.7	79	3.8
Lüders 2008 [37]	PHARAO	high-normal office blood pressure	ACEI Placebo	Ramipril Placebo	3y	62.2±8.2 62.3±7.9	505	5	1.0	1	0.2	2	0.4
							503	2	0.4	1	0.2	1	0.2
Jamerson 2008 [38]	ACCOMPLISH NCT00170950	HTN	ACEI+ CCB(DH) ACEI+DI	Benazepril+amlodipine Benazepril+hydrochlorothiazide	36m	68.4±6.86 68.3±6.86	5744	236	4.1	107	1.9	112	1.9
							5762	262	4.5	134	2.3	133	2.3
ONTARGET 2008 [39]	ONTARGET NCT00153101	vascular disease or high-risk diabetes	ACEI ARB ACEI+ARB	Ramipril Telmisartan Ramipril+Telmisartan	56m	66.4±7.2 66.4±7.1 66.5±7.3	8576	1014	11.8	603	7.0	405	4.7
							8542	989	11.6	598	7.0	369	4.3 4.4
							8502	1065	12.5	620	7.3	373	
Beckett 2008 [40]	HYVET NCT00122811	HTN >80y	ACEI+DI Placebo	Indapamide ±Perindopril Placebo	2.1y	83.6±3.2 83.5±3.1	1933	196	10.1	99	5.1	51	2.6
							1912	235	12.3	121	6.3	69	3.6
ADVANCE 2007 [41]	ADVANCE NCT00145925	T2D+at least 1 risk factor for CVD	ACEI+DI Placebo	Indapamide+Perindopril Placebo	4.3y	66±6 66±7	5569	408	7.3	211	3.8	215	3.9
							5571	471	8.5	257	4.6	218	3.9
Norris 2006 [42]	AASK	hypertensive nephrosclerosis	ACEI BB CCB(DH)	Ramipril Metoprolol Amlodipine	4.1	54.4±10.9 54.9±10.4 54.5±10.7	168	34	20.2	12	7.1	23	13.7
							170	49	28.8	12	7.1	23	13.5
							86	22	25.6	7	8.1	9	10.5
Julius 2006 [43]	VALUE	high-risk HTN	ARB CCB(DH)	Valsartan Amlodipine	3.2y	66.9±8.3 66.8±8.2	3263	297	9.1	78	2.4	108	3.3
							3817	349	9.1	78	2.0	127	3.3
DREAM 2006 [44]	DREAM NCT00095654	impaired fasting glucose levels or impaired glucose tolerance	ACEI Placebo	Ramipril Placebo	3y	54.7±10.9 54.7±10.9	2623	31	1.2	12	0.5	4	0.2
							2646	32	1.2	10	0.4	8	0.3
Suzuki 2005 [45]	E-COST	HTN (35-79y)	ARB non-RASI	Candesartan non-RAS inhibition	3.1y	35–79	1053	4	0.4			47	4.5
							995	4	0.4			77	7.7
Schrader 2005 [46]	MOSES	HTN+Cerebral event	ARB CCB(DH)	Eprosartan Nitrendipine	2.5y	67.7±10.4 68.1±9.5	681	57	8.4	30	4.4	102	15.0
							671	52	7.7	32	4.8	134	20.0
Liu 2005 [47]	FEVER	HTN (50-79y)	CCB(DH)Placebo	Felodipine+ HCTZ Placebo+ HCTZ	40m	61.5±7.1 61.5±7.2	4841	112	2.3	73	1.5	177	3.7
							4870	151	3.1	101	2.1	251	5.2
Dahlöf 2005 [48]	ASCOT-BPLA	HTN (40-79y)	CCB(DH)+ACEI BB+DI	Amlodipine+perindopril Atenolol+bendroflumethiazide	5.5y	40–79	9639	738	7.7	263	2.7	327	3.4
							9618	820	8.5	342	3.6	422	4.4
Poole-Wilson 2004 [49]	ACTION	treated stable symptomatic coronary disease	CCB(DH) Placebo	Nifedipine Placebo	4.9y	63.5±9.3 63.4±9.3	3825	310	8.1	178	4.7	82	2.1
							3840	291	7.6	177	4.6	108	2.8
Nissen 2004 [50]	CAMELOT	CAD+normal blood pressure	CCB(DH) Placebo ACEI	Amlodipine Placebo Enalapril	2y	57.3±9.7 57.2±9.5 58.5±9.9	663	7	1.1	5	0.8	6	0.9
							655	6	0.9	2	0.3	12	1.8
							673	8	1.2	5	0.7	8	1.2
Marre 2004 [51]	DIABHYCAR	T2D+ microalbuminuria+ proteinuria	ACEI Placebo	Ramipril Placebo	4y	65.2±8.4 65.0±8.3	2443	334	13.7	141	5.8	118	4.8
							2469	324	13.1	133	5.4	116	4.7
Lithell 2004 [52]	SCOPE	Elderly HTN	ARB Placebo	Candesartan placebo	3-5y	76.2 ± 4.4 76.5 ± 4.6	1253	123	9.8	69	5.5	31	2.5
							845	109	12.9	63	7.5	27	3.2
PEACE 2004 [53]	PEACE	CAD	ACEI Placebo	Trandolapril Placebo	4.8y	64±8 64±8	4158	299	7.2	146	3.5	71	1.7
							4132	334	8.1	153	3.7	92	2.2
Yusuf 2003 [54]	CHARM-Preserved	CHF+LVEF>40%	ARB placebo	candesartan placebo	36.6m	67.2±11.1 67.1±11.1	1514	244	16.1	170	11.2	58	3.8
							1509	237	15.7	170	11.3	63	4.2
Wing 2003 [55]	ANBP2	HTN (65-84y)	ACEI DI(TT)	Enalapril HCTZ	4.1y	72.0 71.9	3044	195	6.4	84	2.8	120	3.9
							3039	210	6.9	82	2.7	109	3.6
Pfeffer 2003 [56]	VALIANT	MI+LVD/HF	ARB ARB+ACEI ACEI	Valsartan Valsartan+ Captopril Captopril	24.7m	65.0±11.8 64.6±11.9 64.9±11.8	4909	979	19.9	827	16.8	180	3.7
							4885	941	19.3	827	16.9	183	3.7
							4909	958	19.5	830	16.9	211	4.3
Pepine 2003 [57]	INVEST	HTN+CAD	CCB(V) BB	Verapamil Atenolol	2.7y	66.0±9.7 66.1±9.8	11267	873	7.7	431	3.8	176	1.6
							11309	893	7.9	431	3.8	201	1.8
McMurray 2003 [58]	CHARM-Added	CHF+LVEF ≤40%	ARB Placebo	Candesartan+ACEI Placebo+ACEI	41m	64.0±10.7 64.1±11.3	1276	377	29.5	302	23.7	47	3.7
							1272	412	32.4	347	27.3	41	3.2
Malacco 2003 [59]	SHELL	HTN ≥60y	DI(TL) CCB(DH)	Chlorthalidone Lacidipine	32m	72.4± 7.6 72.3± 7.5	940	122	13.0			38	4.0
							942	145	15.4			37	3.9
EURopean 2003 [60]	EUROPA	CAD	ACEI Placebo	Perindopril Placebo	4.2y	60±9 60±9	6110	375	6.1	215	3.5	98	1.6
							6108	420	6.9	249	4.1	102	1.7
Granger 2003 [61]	CHARM-Alternative	CHF+LVEF ≤40%+ intolerance to ACEI	ARB placebo	Candesartan Placebo	33.7m	66.3±11.0 66.8±10.5	1013	265	26.2	219	21.6	36	3.6
							1015	296	29.2	252	24.8	42	4.1
Bulpitt 2003 [62]	HYVET-Pilot	HTN ≥80y	DI(TT) ACEI Placebo	Bendroflumethiazide Lisinopril No treatment	13m	83.8 ±3.3 83.7 ±3.0 83.8 ± 2.9	426	30	7.0	23	5.4	6	1.4
							431	27	6.3	22	5.1	12	2.8
							426	22	5.2	19	4.5	18	4.2
Black 2003 [63]	CONVINCE	HTN	CCB(V) Conventional therapy	Verapamil Atenolol or HCTZ	3y	65.6±7.4 65.6±7.4	8179	337	4.1	153	1.9	133	1.6
							8297	319	3.8	143	1.7	118	1.4
Berl 2003 [64]	IDNT	T2D+HTN	ARB CCB(DH) Placebo	Irbesartan Amlodipine Placebo	2.6y	59.3± 7.1 59.1 ±7.9 58.3 ±8.2	579		0.0	52	9.0	28	4.8
							567		0.0	37	6.5	15	2.6
							569		0.0	46	8.1	26	4.6
Zanchetti 2002 [65]	ELSA	HTN	BB CCB(DH)	Atenolol Lacidipine	3.75y	55.9± 7.5 56.1 ±7.5	1157	17	1.5	8	0.7	14	1.2
							1177	13	1.1	4	0.3	9	0.8
Schrier 2002 [66]	ABCE-N	normotensive T2D	CCB(DH) ACEI	Nisoldipine Enalapril	5.3y	59.1± 0.5 59.4± 0.5	234	19	8.1	8	3.4	11	4.7
							246	19	7.7	14	5.7	6	2.4
ALLHAT 2002 [67]	ALLHAT NCT00000542	HTN ≥55y	DI(TL) CCB(DH) ACEI	Chlorthalidone Amlodipine Lisinopril	4.9y	66.9± 7.7 66.9± 7.7 66.9± 7.7	15255	2187	14.3	992	6.5	675	4.4
							9048	1237	13.7	592	6.5	377	4.2
							9054	1303	14.4	609	6.7	457	5.0
Dickstein 2002 [68]	OPTIMAAL	AMI+HF	ARB ACEI	Losartan Captopril	2.7y	67.6± 9.9 67.2± 9.8	2744	499	18.2	420	15.3	140	5.1
							2733	447	16.4	363	13.3	132	4.8
Dahlöf 2002 [69]	LIFE	HTN(55-80y)+LVH	ARB BB	Losartan+HCTZ Atenolol+HCTZ	4.8y	66.9± 7.0 66.9± 7.0	4605	383	8.3	204	4.4	232	5.0
							4588	431	9.4	234	5.1	309	6.7
Hedblad 2001 [70]	BCAPS	Carotid plaque	BB Placebo	Metoprolol Placebo	36m	61.6± 5.4 61.9±5.3	396	4	1.0			1	0.3
							397	7	1.8			7	1.8
PROGRESS 2001 [71]	PROGRESS-a	Stroke/TIA±HTN	ACEI+DI Placebo	Perindopril+indapamide Placebo	4y	64± 10 64± 10	1770	156	8.8	88	5.0	150	8.5
							1774	187	10.5	121	6.8	255	14.4
	PROGRESS-b	Stroke/TIA±HTN	ACEI Placebo	Perindopril Placebo	4y	64± 10 64± 10	1281					157	12.3
							1280					165	12.9
Pitt 2000 [72]	ELITE II	elderly heart-failure (≥60y)	ARB ACEI	Losartan Captopril	1.5y	71.4±6.7 71.5±6.9	1578	280	17.7	230	14.6	18	1.1
							1574	250	15.9	199	12.6	11	0.7
Pitt 2000 [73]	PREVENT	CAD	CCB(DH) Placebo	Amlodipine Placebo	3y	56.8 57.0	417	6	1.4			5	1.2
							408	8	2.0			5	1.2
Ogihara 2000 [74]	PATE	HTN ≥60y	ACEI CCB(DH)	Delapril Manidipine	3y	70±7 69±7	699	11	1.6	7	1.0	14	2.0
							1049	18	1.7	10	1.0	23	2.2
MacMahon 2000 [75]	PART-2	coronary,cerebrovascular or peripheral vascular disease	ACEI Placebo	Ramipril Placebo	4y	60±8 61±8	308	16	5.2	8	2.6	7	2.3
							309	25	8.1	18	5.8	4	1.3
Hope 2000 [76]	HOPE	high risk for cardiovascular events	ACEI Placebo	Ramipril Placebo	5y	66±7 66±7	4645	482	10.4	282	6.1	156	3.4
							4652	569	12.2	377	8.1	226	4.9
Hansson 2000 [77]	NORDIL	HTN(50-74y)	CCB(Dil) Conventional therapy	Diltiazem BB±DI	4.5y	60.5±6.5 60.3±6.5	5410	231	4.3	131	2.4	159	2.9
							5471	228	4.2	115	2.1	196	3.6
Brown 2000 [78]	INSIGHT	HTN(55-80y)	CCB(DH) DI(TT)	Nifedipine HCTZ+ Amiloride	4y	65±6.5	3157	153	4.8	60	1.9	67	2.1
							3164	152	4.8	52	1.6	74	2.3
Hansson 1999 [79]	CAPPP	HTN	ACEI Conventional therapy	Captopril BB± DI	6.1y	54.2±8.3 52.7±8.4	5492			76	1.4	193	3.5
							5493			95	1.7	199	3.6
Hansson 1999 [80]	STOP-2	HTN(70-84y)	CCB(DH) ACEI Conventional therapy	Felodipine or isradipine Enalapril or lisinopril BB±DI	4y	75.9 76.1 76.0	2196	362	16.5	212	9.7	207	9.4
							2205	380	17.2	226	10.2	215	9.8
							2213	369	16.7	221	10.0	237	10.7
NICS-EH 1999 [81]	NICS-EH	HTN≥60y	CCB(DH) DI(TT)	Nicardipine Trichlormethiazide	5y	≥60	204	2	1.0	2	1.0	1	0.5
							210	2	1.0	0	0.0	0	0.0
Liu 1998 [82]	Syst-China	HTN >60y	CCB±ACEI Placebo	Nitrendipine±Captopril/HCTZ Placebo	2y	≥60	1253	61	4.9	33	2.6	45	3.6
							1141	82	7.2	44	3.9	59	5.2
Estacio 1998 [83]	ABCD	T2D+HTN	CCB(DH) ACEI	Nisoldipine Enalapril	5y	57.2±8.2 57.7±8.4	235	17	7.2	10	4.3	11	4.7
							235	13	5.5	5	2.1	7	3.0
Sun 1997 [84]		HTN	CCB(DH) Placebo	Nitrendipine Placebo	5y	51.8±0.11	1040	48	4.6	11	1.1	37	3.6
							1040	62	6.0	24	2.3	79	7.6
Rosei 1997 [85]	VHAS	HTN	CCB(V) DI(TL)	Verapamil Chlorthalidone	2y	54.5±6.9 53.9±7.0	707	5	0.7	5	0.7	3	0.4
							707	4	0.6	4	0.6	4	0.6
Gong 1996 [86]	STONE	HTN(60-79y)	CCB(DH) Placebo	Nifedipine Placebo	3y	66.6±5.1 67.1±5.6	817	15	1.8	11	1.3	16	2.0
							815	26	3.2	14	1.7	36	4.4
AIRE 1993 [87]	AIRE	HF+AMI	ACEI Placebo	Ramipril Placebo	1.3y	64.9±10.8 65.1±10.8	1004	170	16.9			25	2.5
							982	222	22.6			17	1.7
Dutch TIA 1993 [88]	Dutch TIA	TIA/nondisabling ischemic stroke	BB Placebo	Atenolol Placebo	2.6y		732	64	8.7	41	5.6	52	7.1
							741	58	7.8	33	4.5	62	8.4
MRC 1992 [89]	MRC-2	Elderly HTN (65-74y)	BB DI(TT) Placebo Conventional therapy	Atenolol HCTZ or amiloride Placebo Total active treatment(BB or DI)	5.8y	70.3±5.6 70.2±5.6 70.2±5.6	1102	167	15.2	95	8.6	56	5.1
							1081	134	12.4	66	6.1	45	4.2
							2213	315	14.2	180	8.1	134	6.1
							2183	301	13.8	161	7.4	101	4.6
SOLVD 1992 [90]	SOLVD- Prevention	LVD	ACEI Placebo	Enalapril Placebo	37.4m	59.1 59.1	2111	313	14.8	265	12.6	10	0.5
							2117	334	15.8	298	14.1	13	0.6
SOLVD 1991 [91]	SOLVD	CHF+EF ≤0.35	ACEI Placebo	Enalapril Placebo	41.4m	60.7 61.0	1285	452	35.2	399	31.1	10	0.8
							1284	510	39.7	461	35.9	11	0.9
SHEP 1991 [92]	SHEP	HTN	Conventional therapy Placebo	Chlorthalidone±Atenolol or Reserpine Placebo	4.5y	≥60	2365	213	9.0	90	3.8	106	4.5
							2371	242	10.2	112	4.7	163	6.9
Dahlof 1991 [93]	STOP-Hypertension	Elderly HTN (70-84y)	Conventional therapy Placebo	Atenolol±HCTZ /amiloride Placebo	65m	70–84	812	36	4.4	17	2.1	29	3.6
							815	63	7.7	41	5.0	53	6.5
Perry 1989 [94]	SHEP-Pilot	Elderly HTN (70-84y)	Conventional therapy Placebo	Chlorthalidone±Atenolol or Reserpine Placebo		≥60	443	32	7.2	14	3.2	11	2.5
							108	7	6.5	5	4.6	6	5.6
Australian trial 1987 [95]	Australian trial	HTN(35-65y)	DI(TT) Placebo	Chlorothiazide Placebo	6y	35–65	1721	25	1.5	8	0.5	17	1.0
							1706	35	2.1	18	1.1	31	1.8
Cooper 1986 [96]	HEP	Elderly HTN (60-79y)	Conventional therapy Placebo	Atenolol±bendrofluazide +Methyldopa Placebo	4.4		419	60	14.3	35	8.4	23	5.5
							465	69	14.8	50	10.8	44	9.5
MRC 1985 [97]	MRC-1	HTN(35-64y)	DI(TT) BB Placebo	Bendrofluazide Propranolol Placebo	5.8	51 51 51	4297	128	3.0	59	1.4	18	0.4
							4403	120	2.7	65	1.5	42	1.0
							8654	253	2.9	139	1.6	109	1.3
IPPPSH [98]	IPPPSH	HTN (40-64y)	BB non-BB	Oxprenolo Non-BB	3-5y		3185	108	3.4			45	1.4
							3172	114	3.6			46	1.5
Amery 1985 [99]	EWPHE	HTN≥60y	DI(TT) Placebo	HCTZ +Triamterene Placebo	4.6y	72±8	416	73	17.5	42	10.1	12	2.9
							424	89	21.0	61	14.4	19	4.5
BHAT 1983 [100]	BHAT	AMI	BB Placebo	Propranolol Placebo	2y		1916	138	7.2	127	6.6	29	1.5
							1921	188	9.8	171	8.9	30	1.6
Helgeland 1980 [101]	Oslo	HTN	Conventional therapy Placebo	HCTZ±Alphamethyldopa /Propranolol. Placebo	66m	45.3±2.9 45.2±2.8	406	10	2.5	7	1.7	0	0.0
							379	9	2.4	6	1.6	5	1.3

Abbreviations: NCT,Clinical Trials.gov number; ARB, angiotensin receptor blockers; DI, Diuretics; CCB, calcium channel blockers; ACEI, angiotensin-converting enzyme inhibitor; BB, βadrenergic receptor blockers; HCTZ, Hydrochlorothiazide; RI, renin inhibitor; CV mortality, cardiovascular mortality; CVD, cardiovascular disease; CKD, chronic kidney disease; HTN, hypertension; CAD,coronary artery disease; CHF, chronic heart failure; HF, heart failure; LVEF, left-ventricular ejection fraction; MI, myocardial infarction; LVD, left ventricular systolic dysfunction; AMI, acute myocardial infarction; LVH, Left ventricular hypertrophy; CHD, coronary heart disease; AMI, acute myocardial infarction; EF, ejection fraction; TIA, transient ischemic attack.

### Synthesis of results

The deviance information criteria (DIC) statistics showed that the random effects model provided a better fit to the data than the fixed-effect model. Additionally, for global inconsistency assessment, the consistency model was a better fit for the data than the inconsistency model (S2 Table). Node-splitting method and its relative Bayesian P value were utilized to report the local inconsistency assessment (S3–S11 Tables). All of the P values between direct and indirect comparisons were above 0.05, indicating that our results were reliable.

### Network meta-analysis results

Fig 2 presents the network plot that represents eligible comparisons among the overall population and hypertensive patients. The network meta-analysis evaluated the effect of 21 interventions on stroke and all-cause mortality rates for the overall population: including ACEI, ARB, BB, CCB, conventional therapy, DI, non-BB, non-renin-angiotensin system inhibitors (non-RASI), renin inhibitors (RI), ACEI+BB, ACEI+CCB, ACEI+DI, ARB+ACEI, ARB+ACEI+BB, ARB+BB, ARB+CCB, ARB+DI, BB+DI, CCB+BB, CCB+DI, and placebo. The same interventions, except for RI, were evaluated among hypertensive patients. A total of 20 interventions were utilized to evaluate the cardiovascular mortality in the overall population (ACEI, ARB, BB, CCB, conventional therapy, DI, non-RASI, RI, ACEI+BB, ACEI+CCB, ACEI+DI, ARB+ACEI, ARB+ACEI+BB, ARB+BB, ARB+CCB, ARB+DI, BB+DI, CCB+BB, CCB+DI, and placebo). For hypertensive patients, 19 interventions (excluding RI) were utilized for cardiovascular mortality assessment.

For the overall population (Table 2-1, S19–S27 Tables)

**Table 2:** 2–1 Network meta-analysis results for strokes in the overall population. 2–2 Network meta-analysis results for strokes in hypertensive patients.

**ACEI**																																								
1.78 (0.90, 3.82)	**ACEI+BB**	
**1.43 (1.04, 1.98)**	0.80 (0.36, 1.72)		**ACEI+** **CCB**	
1.12 (0.91, 1.39)	0.63 (0.29, 1.28)		0.79 (0.58, 1.05)		**ACEI+DI**	
1.03 (0.92, 1.14)	0.58 (0.27, 1.15)		**0.72 (0.52, 0.98)**		0.91 (0.74, 1.13)		**ARB**	
1.04 (0.86, 1.25)	0.58 (0.27, 1.16)		0.72 (0.50, 1.04)		0.92 (0.70, 1.20)		1.01 (0.84, 1.21)		**ARB+** **ACEI**	
1.59 (0.81, 3.24)	0.89 (0.38, 2.00)		1.11 (0.53, 2.41)		1.42 (0.70, 2.96)		1.55 (0.78, 3.18)		1.54 (0.77, 3.14)		**ARB+** **ACEI+BB**	
1.07 (0.49, 2.73)	0.60 (0.24, 1.65)		0.75 (0.32, 1.99)		0.95 (0.42, 2.46)		1.04 (0.47, 2.66)		1.03 (0.47, 2.63)		0.67 (0.27, 1.85)		**ARB+BB**	
1.07 (0.60, 1.93)	0.60 (0.23, 1.48)		0.75 (0.39, 1.43)		0.95 (0.52, 1.75)		1.04 (0.58, 1.86)		1.03 (0.56, 1.91)		0.67 (0.27, 1.64)		0.99 (0.34, 2.69)		**ARB+** **CCB**	
1.04 (0.69, 1.57)	0.58 (0.25, 1.28)		0.73 (0.44, 1.20)		0.92 (0.59, 1.44)		1.01 (0.67, 1.52)		1.00 (0.65, 1.57)		0.65 (0.29, 1.43)		0.96 (0.35, 2.36)		0.97 (0.64, 1.49)		**ARB+DI**	
0.93 (0.79, 1.10)	0.52 (0.24, 1.05)		**0.65 (0.46, 0.91)**		0.83 (0.65, 1.06)		0.91 (0.78, 1.07)		0.90 (0.72, 1.14)		0.59 (0.29, 1.17)		0.87 (0.34, 1.93)		0.87 (0.48, 1.59)		0.90 (0.59, 1.37)		**BB**	
1.11 (0.72, 1.72)	0.62 (0.26, 1.41)		0.78 (0.58, 1.05)		0.99 (0.65, 1.50)		1.08 (0.70, 1.68)		1.07 (0.68, 1.73)		0.70 (0.31, 1.55)		1.04 (0.37, 2.54)		1.04 (0.51, 2.15)		1.07 (0.60, 1.93)		1.19 (0.76, 1.87)		**BB+** **DI**	
**1.14 (1.02, 1.28)**	0.64 (0.30, 1.28)		0.80 (0.58, 1.09)		1.02 (0.82, 1.26)		**1.12 (1.00, 1.25)**		1.10 (0.90, 1.36)		0.72 (0.35, 1.42)		1.07 (0.42, 2.36)		1.07 (0.59, 1.92)		1.10 (0.73, 1.66)		**1.23 (1.05, 1.44)**		1.03 (0.67, 1.58)		**CCB**	
0.69 (0.29, 1.67)	0.39 (0.12, 1.20)		0.49 (0.19, 1.22)		0.62 (0.25, 1.50)		0.68 (0.28, 1.61)		0.67 (0.27, 1.63)		0.43 (0.14, 1.34)		0.64 (0.18, 2.12)		0.65 (0.34, 1.23)		0.67 (0.31, 1.45)		0.75 (0.30, 1.80)		0.63 (0.23, 1.64)		0.61 (0.25, 1.45)		**CCB+BB**	
1.59 (0.61, 4.27)	0.89 (0.26, 2.98)		1.11 (0.41, 3.13)		1.41 (0.54, 3.85)		1.55 (0.60, 4.15)		1.53 (0.58, 4.19)		0.99 (0.31, 3.34)		1.47 (0.39, 5.31)		1.48 (0.70, 3.28)		1.53 (0.64, 3.77)		1.70 (0.65, 4.64)		1.43 (0.50, 4.16)		1.39 (0.53, 3.74)		**2.29 (1.13, 4.86)**		**CCB+DI**	
1.12 (0.97, 1.31)	0.63 (0.29, 1.26)		0.78 (0.56, 1.09)		0.99 (0.79, 1.27)		1.09 (0.93, 1.29)		1.08 (0.86, 1.37)		0.70 (0.34, 1.40)		1.04 (0.41, 2.33)		1.05 (0.58, 1.90)		1.08 (0.70, 1.65)		1.20 (0.99, 1.46)		1.01 (0.64, 1.58)		0.98 (0.85, 1.13)		1.61 (0.67, 3.95)		0.70 (0.26, 1.84)		**CT**	
**1.22 (1.06, 1.42)**	0.69 (0.32, 1.39)		0.86 (0.61, 1.20)		1.09 (0.86, 1.38)		**1.19 (1.03, 1.40)**		1.18 (0.95, 1.49)		0.77 (0.37, 1.53)		1.14 (0.45, 2.55)		1.14 (0.63, 2.09)		1.18 (0.77, 1.80)		**1.31 (1.09, 1.59)**		1.10 (0.71, 1.72)		1.07 (0.93, 1.24)		1.76 (0.73, 4.30)		0.77 (0.28, 2.02)		1.09 (0.91, 1.32)		**DI**	
0.93 (0.56, 1.54)	0.52 (0.21, 1.21)		0.65 (0.36, 1.17)		0.82 (0.49, 1.41)		0.90 (0.55, 1.50)		0.89 (0.53, 1.54)		0.58 (0.24, 1.35)		0.86 (0.30, 2.19)		0.87 (0.40, 1.88)		0.89 (0.47, 1.70)		0.99 (0.62, 1.61)		0.84 (0.43, 1.62)		0.81 (0.49, 1.35)		1.34 (0.49, 3.69)		0.58 (0.19, 1.72)		0.83 (0.5, 1.4)		0.76 (0.45, 1.27)		**non** **BB**	
0.78 (0.56, 1.09)	**0.44 (0.19, 0.93)**		**0.55 (0.35, 0.85)**		0.70 (0.48, 1.01)		0.76 (0.56, 1.04)		0.75 (0.53, 1.09)		0.49 (0.22, 1.03)		0.73 (0.28, 1.70)		0.73 (0.38, 1.42)		0.75 (0.45, 1.26)		0.84 (0.59, 1.19)		0.70 (0.41, 1.20)		**0.68 (0.49, 0.95)**		1.12 (0.45, 2.90)		0.49 (0.17, 1.35)		**0.70 (0.49, 0.99)**		**0.64 (0.45, 0.90)**		0.84 (0.46, 1.53)		**non** **RASI**	
**0.84 (0.76, 0.92)**	**0.47 (0.22, 0.94)**		**0.59 (0.43, 0.79)**		**0.75 (0.62, 0.90)**		**0.82 (0.74, 0.90)**		**0.81 (0.67, 0.98)**		0.53 (0.26, 1.04)		0.78 (0.31, 1.72)		0.79 (0.44, 1.40)		0.81 (0.54, 1.20)		0.90 (0.77, 1.04)		0.76 (0.49, 1.15)		**0.73 (0.66, 0.81)**		1.21 (0.51, 2.92)		0.53 (0.20, 1.37)		**0.75 (0.64, 0.87)**		**0.69 (0.59, 0.79)**		0.90 (0.55, 1.49)		1.07 (0.78, 1.49)		**Placebo**			
0.70 (0.49, 1.01)	**0.39 (0.17, 0.85)**		**0.49 (0.31, 0.78)**		**0.62 (0.42, 0.93)**		**0.68 (0.47, 0.98)**		0.67 (0.45, 1.01)		**0.44 (0.20, 0.95)**		0.65 (0.24, 1.55)		0.65 (0.33, 1.29)		0.67 (0.39, 1.15)		0.75 (0.51, 1.10)		0.63 (0.36, 1.09)		**0.61 (0.42, 0.88)**		1.01 (0.39, 2.59)		0.44 (0.15, 1.21)		**0.63 (0.43, 0.91)**		**0.57 (0.39, 0.83)**		0.76 (0.41, 1.38)		0.90 (0.56, 1.45)		0.83 (0.59, 1.19)		**RI**	
**ACEI**																																							
1.42 (0.65, 3.20)		**ACEI+BB**																																					
**1.47 (1.02, 2.11)**		1.03 (0.43, 2.42)		**ACEI+** **CCB**	
1.21 (0.89, 1.62)		0.85 (0.36, 1.93)		0.82 (0.60, 1.13)		**ACEI+DI**	
1.02 (0.85, 1.23)		0.72 (0.31, 1.58)		**0.69 (0.48, 0.99)**		0.84 (0.63, 1.14)		**ARB**	
0.67 (0.31, 1.43)		0.47 (0.21, 1.05)		0.46 (0.20, 1.06)		0.56 (0.25, 1.25)		0.66 (0.30, 1.43)		**ARB+** **ACEI**	
1.27 (0.58, 2.83)		0.89 (0.39, 2.05)		0.87 (0.37, 2.04)		1.06 (0.46, 2.44)		1.25 (0.56, 2.82)		1.89 (0.85, 4.30)		**ARB+** **ACEI+BB**	
0.85 (0.35, 2.27)		0.60 (0.24, 1.61)		0.58 (0.23, 1.65)		0.71 (0.28, 1.96)		0.84 (0.34, 2.26)		1.27 (0.52, 3.39)		0.67 (0.27, 1.79)		**ARB+BB**	
0.97 (0.51, 1.83)		0.68 (0.25, 1.84)		0.66 (0.33, 1.34)		0.80 (0.41, 1.57)		0.95 (0.50, 1.79)		1.43 (0.54, 3.86)		0.76 (0.28, 2.07)		1.13 (0.36, 3.35)		**ARB+** **CCB**	
0.94 (0.60, 1.48)		0.66 (0.26, 1.62)		0.64 (0.37, 1.10)		0.78 (0.48, 1.28)		0.92 (0.59, 1.45)		1.39 (0.59, 3.40)		0.74 (0.30, 1.81)		1.10 (0.38, 2.97)		0.97 (0.62, 1.53)		**ARB+DI**	
0.88 (0.71, 1.09)		0.62 (0.27, 1.36)		**0.60 (0.41, 0.88)**		0.73 (0.54, 1.01)		0.86 (0.71, 1.06)		1.31 (0.61, 2.85)		0.69 (0.31, 1.53)		1.03 (0.38, 2.50)		0.91 (0.48, 1.74)		0.94 (0.59, 1.49)		**BB**	
1.14 (0.69, 1.86)		0.80 (0.31, 1.99)		0.78 (0.56, 1.08)		0.94 (0.60, 1.50)		1.12 (0.68, 1.82)		1.69 (0.69, 4.18)		0.89 (0.36, 2.24)		1.33 (0.45, 3.61)		1.18 (0.54, 2.57)		1.21 (0.64, 2.29)		1.30 (0.78, 2.14)		**BB+** **DI**	
1.11 (0.96, 1.29)		0.78 (0.35, 1.72)		0.76 (0.54, 1.07)		0.92 (0.70, 1.23)		1.09 (0.94, 1.27)		1.66 (0.77, 3.60)		0.88 (0.39, 1.92)		1.31 (0.49, 3.19)		1.15 (0.61, 2.17)		1.19 (0.76, 1.85)		**1.27 (1.05, 1.52)**		0.98 (0.61, 1.58)		**CCB**	
0.62 (0.25, 1.57)		0.44 (0.13, 1.45)		0.43 (0.16, 1.12)		0.52 (0.20, 1.33)		0.61 (0.24, 1.54)		0.93 (0.28, 3.07)		0.49 (0.15, 1.62)		0.73 (0.19, 2.62)		0.65 (0.33, 1.28)		0.66 (0.30, 1.49)		0.71 (0.28, 1.80)		0.55 (0.20, 1.53)		0.56 (0.22, 1.4)		**CCB+BB**	
1.44 (0.52, 4.05)		1.01 (0.28, 3.71)		0.98 (0.34, 2.90)		1.19 (0.43, 3.44)		1.41 (0.51, 3.97)		2.14 (0.61, 7.78)		1.12 (0.32, 4.13)		1.68 (0.42, 6.49)		1.48 (0.68, 3.39)		1.53 (0.62, 3.89)		1.64 (0.59, 4.64)		1.26 (0.42, 3.93)		1.29 (0.47, 3.64)		**2.29 (1.12, 5.02)**		**CCB+DI**	
1.08 (0.91, 1.29)		0.76 (0.34, 1.68)		0.73 (0.51, 1.07)		0.89 (0.67, 1.22)		1.06 (0.87, 1.30)		1.60 (0.75, 3.51)		0.85 (0.38, 1.88)		1.27 (0.47, 3.10)		1.11 (0.59, 2.12)		1.15 (0.73, 1.82)		1.23 (0.98, 1.54)		0.95 (0.58, 1.57)		0.97 (0.83, 1.14)		1.73 (0.69, 4.42)		0.75 (0.27, 2.08)		**CT**	
1.17 (0.99, 1.40)		0.83 (0.37, 1.82)		0.80 (0.56, 1.16)		0.97 (0.73, 1.33)		1.15 (0.95, 1.41)		1.75 (0.81, 3.82)		0.93 (0.41, 2.06)		1.38 (0.51, 3.38)		1.21 (0.65, 2.31)		1.25 (0.80, 1.97)		**1.34 (1.08, 1.66)**		1.03 (0.63, 1.70)		1.05 (0.90, 1.24)		1.88 (0.75, 4.79)		0.82 (0.29, 2.26)		1.09 (0.89, 1.33)		**DI**	
0.88 (0.51, 1.51)		0.62 (0.24, 1.58)		0.60 (0.32, 1.12)		0.73 (0.41, 1.32)		0.86 (0.50, 1.48)		1.31 (0.53, 3.29)		0.69 (0.27, 1.77)		1.03 (0.34, 2.84)		0.91 (0.40, 2.06)		0.94 (0.48, 1.86)		1.00 (0.60, 1.65)		0.77 (0.38, 1.57)		0.79 (0.46, 1.34)		1.41 (0.49, 4.10)		0.61 (0.19, 1.91)		0.82 (0.47, 1.41)		0.75 (0.43, 1.29)		**non** **BB**	
0.78 (0.54, 1.13)		0.55 (0.23, 1.29)		**0.53 (0.33, 0.87)**		0.65 (0.42, 1.01)		0.77 (0.56, 1.07)		1.16 (0.50, 2.72)		0.62 (0.26, 1.44)		0.92 (0.32, 2.36)		0.81 (0.40, 1.66)		0.83 (0.48, 1.45)		0.89 (0.61, 1.30)		0.68 (0.39, 1.24)		**0.70 (0.49, 1.00)**		1.25 (0.47, 3.38)		0.54 (0.18, 1.56)		0.73 (0.50, 1.06)		**0.66 (0.46, 0.97)**		0.89 (0.47, 1.67)		**non** **RASI**			
**0.76 (0.64, 0.89)**		0.53 (0.24, 1.17)		**0.52 (0.37, 0.72)**		**0.63 (0.49, 0.81)**		**0.75 (0.64, 0.87)**		1.13 (0.52, 2.46)		0.60 (0.27, 1.32)		0.89 (0.33, 2.16)		0.78 (0.42, 1.45)		0.81 (0.53, 1.23)		0.86 (0.71, 1.04)		0.67 (0.41, 1.06)		**0.68 (0.59, 0.77)**		1.22 (0.49, 3.05)		0.53 (0.19, 1.44)		**0.71 (0.59, 0.83)**		**0.65 (0.55, 0.75)**		0.86 (0.50, 1.47)		0.97 (0.67, 1.38)		**Placebo**	

Abbreviations: CrI, credible interval; ARB, angiotensin receptor blockers; DI, Diuretics; DI(TL), thiazide-like diuretics; DI(TT), thiazide-type diuretics; CCB, calcium channel blockers; CCB(DH), dihydropyridine calcium channel blockers; CCB(D), calcium channel blockers (diltiazem); CCB(V), calcium channel blockers (verapamil); ACEI, angiotensin-converting enzyme inhibitor; BB, β adrenergic receptor blockers; nonRASI, non-renin-angiotensin system (RAS) inhibitors; RI, renin inhibitors. Effect sizes represent summary relative risk and 95% credible intervals. Bold values indicate significant results. Values greater than 1 favor the treatment in the corresponding column, whereas values less than 1 favor the treatment in the corresponding row.

The study included a total of 88 trials (n = 487,076) to analyze stroke incidence, 85 trials (n = 471,815) to assess all-cause mortality rates, and 78 trials (n = 463,452) to examine cardiovascular mortality rates in the overall population.

ACEIs, ARBs, CCBs, and DIs exhibited superior efficacy in reducing stroke, all-cause mortality, and cardiovascular mortality compared to placebo. Conversely, BBs demonstrated no efficacy in preventing stroke, all-cause mortality, and cardiovascular mortality when compared to placebo. Furthermore, CCBs were significantly more effective than BBs, ACEIs, and ARBs in reducing stroke, and more effective than ARBs in reducing cardiovascular mortality. DIs were also significantly more effective than BBs, ACEIs, and ARBs in reducing stroke, and more effective than both ARBs and BBs in reducing cardiovascular mortality. Moreover, ACEIs were significantly more effective than BBs and ARBs in reducing all-cause mortality, and more effective than ARBs in reducing cardiovascular mortality. The combination of ACEIs and CCBs was more effective in reducing stroke than monotherapy with ACEIs or ARBs, more effective in reducing all-cause mortality than monotherapy with ACEIs, ARBs, CCBs, or DIs, and more effective in reducing cardiovascular mortality than monotherapy with ACEIs, ARBs, or CCBs.

For hypertensive patients (Table 2-2, S19–S27 Tables)

The analysis for hypertensive patients included 58 trials (n = 305,171) investigating stroke incidence, 54 trials (n = 289,358) assessing all-cause mortality rates, and 49 trials (n = 272,622) examining cardiovascular mortality rates.

ACEIs, CCBs, and DIs were significantly more effective than placebo in reducing stroke, all-cause mortality, and cardiovascular mortality. ARBs were significantly more effective than placebo in reducing stroke and all-cause mortality but were ineffective in reducing cardiovascular mortality. BBs showed no efficacy compared to placebo in preventing stroke, all-cause mortality, and cardiovascular mortality. Furthermore, CCBs were significantly more effective than BBs in reducing stroke, all-cause mortality, and cardiovascular mortality. DIs were significantly more effective than BBs in reducing stroke, and cardiovascular mortality. ACEIs were significantly more effective than BBs in reducing all-cause mortality. Additionally, the combination of ACEIs and CCBs was significantly more effective in reducing stroke than monotherapy with ACEIs or ARBs, more effective in reducing all-cause mortality than monotherapy with ACEIs, ARBs, CCBs, or DIs, and more effective than ARBs in reducing cardiovascular mortality.

In subgroup analyses within the diuretic class, the subclass of thiazide-type diuretics showed no efficacy compared to placebo in preventing all-cause mortality. Within the calcium channel blocker class, the effects of preventing stroke, all-cause mortality, and cardiovascular mortality were limited to the dihydropyridine subclass. Additionally, the dihydropyridine subclass was more effective than the verapamil subclass in preventing cardiovascular mortality.

### Publication bias

Comparison-adjusted funnel plots are provided in S3–S11 Figs, and all of them were visually symmetrical, indicating no dominant publication bias.

### Quality of the evidence

The results of Confidence in Network Meta-analysis (CINeMA) evaluations are presented in S12–S17 Tables. The overall quality of evidence for which we could assess ranged from very low to high. The main reasons for downgrading the certainty of evidence were within-study bias, imprecision, incoherence, and heterogeneity. The evidence of within-study bias was due to inadequate reporting of randomization and blinding. The evidence for imprecision, heterogeneity, and incoherence was identified because of the limited number of trials available for analysis (S18 Table).

## Discussion

The present meta-analysis employed a refined classification of antihypertensive medications to assess the effectiveness of all available antihypertensive agents in preventing strokes. This study provided a thorough systematic review and network meta-analysis to enrich the current knowledge on the optimal selection of antihypertensive medications for hypertensive patients. In the overall population, we found that ACEIs, ARBs, CCBs, and diuretics demonstrated superiority over placebo in reducing stroke, all-cause mortality, and cardiovascular mortality. CCBs and diuretics outperformed BBs, ACEIs, and ARBs in reducing the risk of stroke. However, when focusing on hypertensive patients, ACEIs, CCBs, and diuretics were found to be superior to placebo in reducing stroke, all-cause mortality, and cardiovascular mortality. ARBs reduced stroke and all-cause mortality but lacked efficacy in reducing cardiovascular mortality. Among the various CCB subclasses, only the dihydropyridines showed efficacy in preventing stroke, all-cause mortality, and cardiovascular mortality. Among diuretic subclasses, thiazide-type diuretics exhibited no efficacy in preventing all-cause mortality. The combination of ACEIs with CCBs proved more effective than monotherapy with ACEIs or ARBs in reducing stroke, more effective than monotherapy with ACEIs, ARBs, CCBs, or diuretics in reducing all-cause mortality, and more effective than ARBs in reducing cardiovascular mortality.

The Renin-Angiotensin System (RAS) is a well-known mechanism in the development of hypertension. As a result, antihypertensive agents that inhibit the RAS have been widely utilized for managing hypertension [102]. In 2018, Chen et al. conducted a meta-analysis to explore the efficacy and safety of RAS inhibitors in comparison to other antihypertensive drug classes among hypertensive patients. The authors reported that first-line thiazides and CCBs led to a lower incidence of strokes compared to first-line RAS inhibitors. Moreover, first-line RAS inhibitors were superior to first-line BBs for reducing strokes, but the study did not distinguish between ACEIs and ARBs [103]. Dimou et al. conducted a network meta-analysis that examined the relative effectiveness of ACEIs and ARBs in essential hypertension. The study showed that ACEIs and ARBs had a similar effect on all-cause mortality, cardiovascular mortality, and stroke [104]. Another meta-analysis by Thomopoulos et al. examined 50 trials involving 247,006 participants with hypertension prevalence of over 40%. The study indicated that RAS inhibitors were more effective than placebos and BBs but less effective than diuretics and CCBs in preventing strokes. ACEIs were superior to placebo, but inferior to CCBs and other classes, whereas ARBs were superior to placebo and BBs [105]. Similarly, our study found that ARBs and ACEIs were superior to placebos but inferior to diuretics and CCBs in the prevention of strokes in the overall population. However, when specifically analyzing hypertensive patients, we discovered that ARBs and ACEIs exhibited a comparable effectiveness to diuretics and CCBs in reducing strokes and all-cause mortality. Nonetheless, ARBs did not show any impact on cardiovascular mortality.

Calcium ions contribute to tissue damage in the heart and other organs, leading to stroke and myocardial infarction. CCBs are widely used in the treatment of angina and hypertension. Several meta-analyses have evaluated the effects of CCBs on both cardiovascular and cerebrovascular outcomes. Costanzo et al. (2009) conducted a meta-analysis to investigate the impacts of CCBs on cardiovascular outcomes in comparison with other drugs. Their analysis confirmed that the use of CCBs reduced the risk of stroke compared to ACEIs, without increasing the risk of cardiovascular death, myocardial infarction, or major cardiovascular events [106]. Chen et al. (2010) revealed in a Cochrane systematic review that CCBs reduced the risk of stroke compared to ACEIs, the risk of stroke and myocardial infarction compared to ARBs, and the risk of stroke and cardiovascular mortality compared to BBs [107]. However, Chen et al.’s (2013) meta-analysis showed that although CCBs reduces stroke risk in hypertensive patients when compared to placebo and BBs, it shows no significant difference when compared to ACEIs and diuretics [108]. The meta-analysis conducted by Thomopoulos et al., involving 247,006 participants with hypertension prevalence over 40%, reported that CCBs were more effective than placebos, BBs, ACEIs, RAS blockers, and all other classes together in preventing stroke [105]. Wei et al. (2020) conducted a network meta-analysis and reported that ACEIs, dihydropyridine CCBs, and diuretics had similar effects on cardiovascular mortality and stroke [109]. A meta-analysis, synthesizing data from 13 clinical studies involving 103,793 participants, indicated a significant reduction in stroke risk for individuals treated with dihydropyridine CCBs compared to those receiving non-dihydropyridine CCBs or other antihypertensive drugs. The associated meta-regression analysis revealed that the decrease in stroke risk attributed to dihydropyridine CCBs is independent of the extent of systolic blood pressure reduction. This decrease might be partially due to the potential neuroprotective and preventive effects of CCBs on the progression of carotid atherosclerosis. In addition, dihydropyridine CCBs, such as benidipine, are also known to inhibit the production of reactive oxygen species derived from polymorphonuclear leukocytes in high-salt-loaded spontaneously hypertensive rats. This inhibition is facilitated by their antioxidant capacity and the ability to suppress the Ca^2^⁺/protein kinase C/nicotinamide adenine dinucleotide phosphate (NADPH) oxidase signaling pathway [110]. Our study also verified that CCBs were more effective than placebos, BBs, ACEIs, and ARBs in preventing strokes in the overall population. Conversely, when specifically examining hypertensive patients, CCBs demonstrated comparable effects to diuretics, ACEIs, and ARBs in terms of both all-cause mortality and strokes. Moreover, our findings indicate that, among the different subclasses of CCBs, only the dihydropyridine subclass showed effectiveness in preventing stroke, all-cause mortality, and cardiovascular mortality compared to both placebos and BBs. Additionally, the dihydropyridine subclass demonstrated superior efficacy in preventing cardiovascular mortality compared to the verapamil subclass.

Thiazide diuretics, including thiazide-type (chlorothiazide, hydrochlorothiazide, bendroflumethiazide, trichlormethiazide, and bendrofluazide) and thiazide-like diuretics (indapamide and chlorthalidone), have been used to treat hypertension for over five decades. Chen et al. conducted a 2015 meta-analysis to investigate the cardioprotective effects of thiazide diuretics, including thiazide-type and thiazide-like diuretics, in hypertensive patients. The authors reported that diuretics were associated with reduced risks of cardiovascular diseases and heart failure but showed no significant differences in stroke risk when compared to the control group [111]. However, Wright et al.’s (2018) meta-analysis showed that first-line low-dose thiazides were effective in reducing the risk of mortality, stroke, and heart attack compared to control group [112]. Thomopoulos et al.’s meta-analysis revealed that diuretics were more effective than placebos and RAS blockers in preventing strokes [105]. Our study also confirmed that diuretics exhibited superior efficacy compared to placebos, BBs, ACEIs, and ARBs in preventing strokes in the overall population. However, in the specific examination of hypertensive patients, diuretics demonstrated similar effects to CCBs, ACEIs, and ARBs in terms of both all-cause mortality and strokes. Moreover, in subgroup analyses conducted within the diuretic class, the subclass of thiazide-type diuretics exhibited no efficacy in preventing all-cause mortality.

For more than four decades, beta blockers (BBs) have served as the standard hypertension treatment. However, recent evidence suggests that their effectiveness as a first-line therapy is debatable, as they have not demonstrated sufficient cardiovascular protection in randomized placebo-controlled trials. According to Wiysonge et al.’s (2017) Cochrane systematic review, first-line BBs modestly reduce stroke in hypertensive individuals, but are not associated with significant effects on total mortality or coronary heart disease. Additionally, their efficacy at stroke prevention is less notable than CCBs and renin-angiotensin system inhibitors [113]. Furthermore, Thomopoulos et al. (2020) conducted a meta-analysis which demonstrated that BBs are less effective than other antihypertensive drugs in preventing stroke and all-cause mortality across all trials, as well as trials conducted exclusively among hypertensive patients [114]. Our study also presents evidence indicating that BBs were ineffective in preventing stroke, all-cause mortality, and cardiovascular mortality among both the overall population and hypertensive patients.

Drug combinations are recommended in hypertension guidelines for most patients due to their superior blood pressure lowering effect compared to monotherapy. Thomopoulos et al.’s meta-analysis revealed that antihypertensive treatments based on two or more drugs are associated with a reduction in the risk of all or most hypertension-related outcomes compared with simpler treatment regimens [115]. Zhong et al. concluded that diuretics, CCBs, and ARBs, either alone or in combination, can be considered as first-line treatments for stroke prevention [6]. Our study has confirmed that combining ACEI and CCB can provide additional benefits for stroke prevention, reducing the risk of all-cause mortality more effectively than other antihypertensive medications used alone.

### Strengths and limitations

Our analysis was different in many aspects from other meta-analyses. First, we employed a refined classification of antihypertensive medications to assess the effectiveness of all available antihypertensive agents. For example, we clearly distinguished conventional therapy, nonRASI treatment, and nonBB treatment, instead of grouping these treatments together as a control. Second, we further analysed differences in the effectiveness between subgroups of calcium channel blockers and between subgroups of thiazide diuretics. Third, we not only compared the effects of monotherapy with different classes of antihypertensive drugs on preventing stroke events but also examined the effects of combination therapy with different classes of antihypertensive drugs on preventing stroke events.

There are several limitations in this network meta-analysis. First, the approach to antihypertensive treatment has evolved in response to changing perspectives on hypertension over the last three decades. Differences have been noted in the types of antihypertensive medications, their dosages, and the therapeutic goals for stroke prevention in older versus more recent trials. However, the scarcity of information on this particular aspect in clinical trials limits the comparative analysis of older and newer trials. Second, most of the comparison arms in our network had only one trial connecting them; more studies in the future would lead to more precise and accurate estimates. Third, due to the limited information, we did not perform further analysis to compared the effects of antihypertensive drugs according to gender, race. Forth, no comparison between patients with and without diabetes or with and without hyperlipidemia was possible for the different drug classes since insufficient data were available in each drug class for patients with diabetes or hyperlipidemia.

## Conclusions

In conclusion, the current evidence demonstrated that ACEIs, dihydropyridine CCBs, and thiazide-like diuretics may provide superior prevention against stroke, all-cause mortality, and cardiovascular mortality in patients with hypertension. The combination of ACEIs and CCBs may provide enhanced protection of stroke than ACEIs or ARBs monotherapy. The results of this study may help physicians determine the best treatment options for their hypertensive patients for stroke prevention.

## Supporting information

S1 PRISMA NMA checklist Search strategy. (DOC)

S1 Table Search strategy. (DOCX)

S2 Table Model fit statistics. (DOCX)

S3 Table Node-splitting results for stroke in the overall population. (DOCX)

S4 Table Node-splitting results for all-cause mortality in the overall population. (DOCX)

S5 Table Node-splitting results for cardiovascular mortality in the overall population. (DOCX)

S6 Table Node-splitting results for stroke in hypertensive patients. (DOCX)

S7 Table Node-splitting results for all-cause mortality in hypertensive patients. (DOCX)

S8 Table Node-splitting results for cardiovascular mortality in hypertensive patients. (DOCX)

S9 Table Node-splitting results for subgroup analysis of stroke in hypertensive patients. (DOCX)

S10 Table Node-splitting results for subgroup analysis of all-cause mortality in hypertensive patients. (DOCX)

S11 Table Node-splitting results for subgroup analysis of cardiovascular mortality in hypertensive patients. (DOCX)

S12 Table Outcomes of CINeMA evaluation regarding the outcome of stroke in the overall population. (DOCX)

S13 Table Outcomes of CINeMA evaluation regarding the outcome of all-cause mortality in the overall population. (DOCX)

S14 Table Outcomes of CINeMA evaluation regarding the outcome of cardiovascular mortality in the overall population. (DOCX)

S15 Table Outcomes of CINeMA evaluation regarding the outcome of stroke in hypertensive patients. (DOCX)

S16 Table Outcomes of CINeMA evaluation regarding the outcome of all-cause mortality in hypertensive patients. (DOCX)

S17 Table Outcomes of CINeMA evaluation regarding the outcome of cardiovascular mortality in hypertensive patients. (DOCX)

S18 Table Reasons for downgrading the confidence in the results. (DOCX)

S19 Table Relative risk [RR] with 95% CrI for stroke of the overall population. (DOCX)

S20 Table Relative risk [RR] with 95% CrI for stroke of hypertensive patients. (DOCX)

S21 Table Relative risk [RR] with 95% CrI for subgroup analysis of stroke among hypertensive patients. (DOCX)

S22 Table Relative risk [RR] with 95% CrI for all-cause mortality of the overall population. (DOCX)

S23 Table Relative risk [RR] with 95% CrI for all-cause mortality of hypertensive patients. (DOCX)

S24 Table Relative risk [RR] with 95% CrI for subgroup analysis of all-cause mortality among hypertensive patients. (DOCX)

S25 Table Relative risk [RR] with 95% CrI for cardiovascular mortality of the overall population. (DOCX)

S26 Table Relative risk [RR] with 95% CrI for cardiovascular mortality of hypertensive patients. (DOCX)

S27 Table Relative risk [RR] with 95% CrI for subgroup analysis of cardiovascular mortality among hypertensive patients. (DOCX)

S28 Table Studies included and excluded. (XLSX)

S29 Table Risk of bias for each study. (XLS)

S30 Table Raw data used in current meta-analysis. (XLS)

S1 Fig Risk of bias summary for RCTs. (DOCX)

S2 Fig Risk of bias graph for RCTs. (DOCX)

S3 Fig Comparison-adjusted funnel plots regarding the outcome of stroke in the overall population. (DOCX)

S4 Fig Comparison-adjusted funnel plots regarding the outcome of stroke in hypertensive patients. (DOCX)

S5 Fig Comparison-adjusted funnel plots regarding the outcome of subgroup analysis of stroke in hypertensive patients. (DOCX)

S6 Fig Comparison-adjusted funnel plots regarding the outcome of all-cause mortality in the overall population. (DOCX)

S7 Fig Comparison-adjusted funnel plots regarding the outcome of all-cause mortality in hypertensive patients. (DOCX)

S8 Fig Comparison-adjusted funnel plots regarding the outcome of subgroup analysis of all-cause mortality in hypertensive patients. (DOCX)

S9 Fig Comparison-adjusted funnel plots regarding the outcome of cardiovascular mortality in the overall population. (DOCX)

S10 Fig Comparison-adjusted funnel plots regarding the outcome of cardiovascular mortality in hypertensive patients. (DOCX)

S11 Fig Comparison-adjusted funnel plots regarding the outcome of subgroup analysis of cardiovascular mortality in hypertensive patients. (DOCX)

## Abbreviations

ARBs: angiotensin receptor blockers
DIs: Diuretics
CCBs: calcium channel blockers
ACEIs: angiotensin-converting enzyme inhibito
BBs: β adrenergic receptor blockers
RCT: randomized controlled trial
