# Expression of Concern: Comparative efficacy of different antihypertensive drug classes for stroke prevention: A network meta-analysis of randomized controlled trials

**DOI:** 10.1371/journal.pone.0343104

**Published:** 2026-02-17

**Authors:** The PLOS One Editors

After this article [1] was published, concerns were noted that the articles cited as References 19 and 29 respectively were retracted before [1] was published.

In addition, the *PLOS One* Editors have concerns about the article’s compliance with the PLOS Authorship policy.

In light of the cumulative issues, the *PLOS One* Editors issue this Expression of Concern. Readers are advised to interpret the article [1] with caution.
