# Supplementary material for: Comparative efficacy of different antihypertensive drug classes for stroke prevention: A network meta-analysis of randomized controlled trials
Source: PLoS One. 2025 Feb 21;20(2):e0313309. doi: 10.1371/journal.pone.0313309 (PMC11845040; doi:10.1371/journal.pone.0313309)
Supplement: S1 Table — (DOCX) [file pone.0313309.s002.docx]

**S1 Table. Search strategy**

**PUBMED:**

(

("stroke"[MeSH Terms] OR "strokes"[Title/Abstract] OR "cerebrovascular accident"[Title/Abstract] OR "cerebrovascular accidents"[Title/Abstract] OR "CVA"[Title/Abstract] OR "CVAs"[Title/Abstract] OR "cerebrovascular apoplexy"[Title/Abstract] OR "apoplexy"[Title/Abstract] OR "cerebrovascular"[Title/Abstract] OR "vascular accident"[Title/Abstract] OR "brain vascular accident"[Title/Abstract] OR "brain vascular accidents"[Title/Abstract] OR "vascular accidents"[Title/Abstract] OR "cerebrovascular stroke"[Title/Abstract] OR "cerebrovascular strokes"[Title/Abstract] OR "cerebrovascular"[Title/Abstract] OR "cerebral stroke"[Title/Abstract] OR "cerebral strokes"[Title/Abstract] OR "acute stroke"[Title/Abstract] OR "acute cerebrovascular accident"[Title/Abstract] OR "acute cerebrovascular accidents"[Title/Abstract])

AND

(

"Antihypertensive"[Title/Abstract] OR "Anti-Hypertensive"[Title/Abstract] OR "blood pressure lowering"[Title/Abstract] OR "blood pressure lowering"[Title/Abstract] OR "blood pressure lowering"[Title/Abstract] OR "antihypertensive agents"[Title/Abstract] OR "antihypertensive agents"[MeSH Terms]

OR

"Diuretic"[Title/Abstract] OR "Diuretics"[Title/Abstract]

OR

"beta block"[Title/Abstract] OR "beta adrenergic antagonist"[Title/Abstract] OR "beta adrenergic receptor antagonist"[Title/Abstract] OR "beta adrenergic block"[Title/Abstract] OR "beta adrenergic receptor block"[Title/Abstract] OR "beta adrenoreceptor antagonist"[Title/Abstract] OR "adrenergic beta antagonist"[Title/Abstract] OR "beta receptor block"[Title/Abstract]

OR

"calcium channel block"[Title/Abstract] OR "calcium channel block"[Title/Abstract] OR "calcium channel antagonist"[Title/Abstract]

OR "Angiotensin-Converting Enzyme Inhibitor"[Title/Abstract] OR "Angiotensin I-Converting Enzyme Inhibitor"[Title/Abstract]

OR

"Angiotensin receptor antagonist"[Title/Abstract] OR "Angiotensin II Receptor Antagonist"[Title/Abstract]

OR

"antihypertensive agents"[Pharmacological Action] OR "Diuretics"[Pharmacological Action] OR "Diuretics"[MeSH Terms] OR "adrenergic beta antagonists"[Pharmacological Action] OR "adrenergic beta antagonists"[MeSH Terms] OR "calcium channel blockers"[Pharmacological Action] OR "calcium channel blockers"[MeSH Terms] OR "angiotensin converting enzyme inhibitors"[Pharmacological Action] OR "angiotensin converting enzyme inhibitors"[MeSH Terms] OR "angiotensin receptor antagonists"[Pharmacological Action] OR "angiotensin receptor antagonists"[MeSH Terms]

)

)

AND

(randomizedcontrolledtrial[Filter])

**EMBASE:**

| **#** | **Searches** |
| --- | --- |
| 1 | ‘Antihypertensive’:ab,ti OR ‘Anti-Hypertensive’ :ab,ti |
| 2 | ‘Diuretic’ :ab,ti OR ‘Diuretics’ :ab,ti |
| 3 | ‘Adrenergic beta-Antagonists’ :ab,ti |
| 4 | ‘Calcium Channel Blockers’ :ab,ti |
| 5 | ‘Angiotensin-Converting Enzyme Inhibitors’ :ab,ti |
| 6 | ‘Angiotensin Receptor Antagonists’ :ab,ti |
| 7 | 'strokes' :ab,ti OR 'stroke' :ab,ti OR 'cerebrovascular accidents' :ab,ti OR 'cerebrovascular apoplexy:ab,ti ' OR 'apoplexy, cerebrovascular' :ab,ti OR 'vascular accident, brain' :ab,ti OR 'cerebrovascular stroke' :ab,ti OR 'cerebrovascular strokes' :ab,ti OR 'stroke, cerebrovascular' :ab,ti OR 'cerebrovascular accident':ab,ti |
| 8 | 1 OR 2 OR 3 OR 4 OR 5 OR 6 |
| 9 | 7 AND 8 |

**Cochrane Library Central Register of Controlled Trials**

#1(strokes):ti,ab,kw OR (stroke):ti,ab,kw

#2 ("anti-hypertensive"):ti,ab,kw OR ("antihypertensive"):ti,ab,kw

#3("angiotensin-converting enzyme inhibitors"):ti,ab,kw

#4 ("angiotensin receptor blocker"):ti,ab,kw OR (Angiotensin Receptor Antagonists):ti,ab,kw

#5 ("calcium-channel blockers"):ti,ab,kw

#6 ("adrenergic beta blocker"):ti,ab,kw

#7 ("diuretic"):ti,ab,kw

#8 #2 OR #3 OR #4 OR #5 OR #6 OR #7

#8 #1 AND #8
