# Supplementary material for: Comparative efficacy of different antihypertensive drug classes for stroke prevention: A network meta-analysis of randomized controlled trials
Source: PLoS One. 2025 Feb 21;20(2):e0313309. doi: 10.1371/journal.pone.0313309 (PMC11845040; doi:10.1371/journal.pone.0313309)
Supplement: S2 Table — (DOCX) [file pone.0313309.s003.docx]

**S2 Table. Model fit statistics.**

|  | DIC | | |
| --- | --- | --- | --- |
|  | Fixed effect model(consistency) | Random effects model  (consistency) | Random effects model  (inconsistency) |
| **For the overall population** |  |  |  |
| Stroke | 366.4738 | 333.6836 | 341.0748 |
| All-cause mortality | 306.4542 | 301.8931 | 315.3689 |
| Cardiovascular mortality | 289.4715 | 287.648 | 302.8951 |
| **For hypertensive patients** |  |  |  |
| Stroke | 250.2323 | 227.5676 | 238.2336 |
| All-cause mortality | 199.3686 | 199.2334 | 206.48 |
| Cardiovascular mortality | 184.8394 | 185.217 | 199.1637 |
| **Subgroup analyses** |  |  |  |
| Stroke | 248.7779 | 228.2949 | 243.832 |
| All-cause mortality | 199.9572 | 200.0895 | 216.1458 |
| Cardiovascular mortality | 183.3051 | 184.822 | 206.8942 |

Abbreviations: DIC, Deviance information criterion ( lower values preferred).
