# Supplementary material for: Comparative efficacy of different antihypertensive drug classes for stroke prevention: A network meta-analysis of randomized controlled trials
Source: PLoS One. 2025 Feb 21;20(2):e0313309. doi: 10.1371/journal.pone.0313309 (PMC11845040; doi:10.1371/journal.pone.0313309)
Supplement: S3 Table — (DOCX) [file pone.0313309.s004.docx]

**S3 Table. Node-splitting results for stroke in the overall population.**

| **Comparison** | **NMA  mean difference** | **Direct  mean difference** | **Indirect  mean difference** | ***p-value*** |
| --- | --- | --- | --- | --- |
| ACEI vs.ARB | -0.047 (-0.23, 0.14) | 0.0099 (-0.14, 0.16) | -0.013 (-0.13, 0.099) | 0.63265 |
| ACEI vs.ARB+ACEI | -0.082 (-0.30, 0.14) | 0.022 (-0.69, 0.75) | -0.020 (-0.21, 0.17) | 0.7887 |
| ACEI vs.BB | -0.14 (-0.68, 0.40) | 0.11 (-0.072, 0.29) | 0.082 (-0.091, 0.25) | 0.392025 |
| ACEI vs.CCB | -0.040 (-0.23, 0.16) | -0.17 (-0.33, -0.021) | -0.12 (-0.24, -0.0047) | 0.2798 |
| ACEI vs.Conventional therapy | 0.062 (-0.17, 0.29) | -0.23 (-0.44, -0.036) | -0.10 (-0.26, 0.047) | 0.051775 |
| ACEI vs.DI | -0.15 (-0.38, 0.078) | -0.22 (-0.43, -0.018) | -0.18 (-0.33, -0.035) | 0.631325 |
| ACEI vs.nonRASI | -0.72 (-2.7, 1.0) | 0.33 (-0.023, 0.68) | 0.29 (-0.046, 0.63) | 0.2444 |
| ACEI vs.Placebo | 0.14 (-0.0036, 0.28) | 0.27 (0.13, 0.42) | 0.21 (0.11, 0.31) | 0.201775 |
| ACEI+CCB vs.ACEI+DI | 0.17 (-0.19, 0.54) | 0.48 (-0.046, 1.0) | 0.27 (-0.035, 0.57) | 0.340425 |
| ACEI+CCB vs.CCB | 0.49 (-0.091, 1.1) | 0.15 (-0.24, 0.53) | 0.25 (-0.074, 0.58) | 0.337125 |
| ACEI+CCB vs.Placebo | 0.63 (-0.15, 1.5) | 0.57 (0.24, 0.92) | 0.58 (0.26, 0.90) | 0.9003 |
| ACEI+DI vs.Placebo | 0.28 (0.070, 0.48) | 0.58 (-0.011, 1.2) | 0.31 (0.11, 0.51) | 0.347325 |
| ARB vs.ARB+ACEI | 0.015 (-0.20, 0.24) | 0.44 (-0.35, 1.2) | -0.0066 (-0.20, 0.19) | 0.309 |
| ARB vs.BB | 0.29 (-0.024, 0.61) | 0.020 (-0.17, 0.21) | 0.095 (-0.071, 0.26) | 0.138125 |
| ARB vs.CCB | 0.040 (-0.15, 0.23) | -0.19 (-0.33, -0.043) | -0.11 (-0.22, 0.0099) | 0.063325 |
| ARB vs.nonRASI | 0.34 (0.011, 0.66) | -0.68 (-2.7, 1.0) | 0.31 (-0.016, 0.63) | 0.25985 |
| ARB+ACEI vs.BB | -0.83 (-2.2, 0.23) | 0.15 (-0.099, 0.39) | 0.10 (-0.14, 0.34) | 0.08175 |
| BB vs.CCB | -0.19 (-0.49, 0.10) | -0.21 (-0.42, -0.0021) | -0.20 (-0.36, -0.034) | 0.895225 |
| BB vs.DI | -0.44 (-0.81, -0.069) | -0.22 (-0.44, 0.0070) | -0.26 (-0.45, -0.071) | 0.314575 |
| BB vs.Placebo | 0.21 (-0.0079, 0.43) | 0.078 (-0.13, 0.29) | 0.13 (-0.025, 0.29) | 0.38735 |
| CCB vs.Conventional therapy | 0.076 (-0.12, 0.27) | -0.092 (-0.33, 0.13) | 0.017 (-0.14, 0.16) | 0.26485 |
| CCB vs.DI | 0.065 (-0.16, 0.28) | -0.18 (-0.39, 0.026) | -0.062 (-0.21, 0.084) | 0.111775 |
| CCB vs.Placebo | 0.46 (0.27, 0.66) | 0.26 (0.14, 0.40) | 0.33 (0.22, 0.44) | 0.089925 |
| DI vs.Placebo | 0.53 (0.33, 0.75) | 0.26 (0.067, 0.46) | 0.39 (0.25, 0.54) | 0.06005 |

Abbreviations: ARB, angiotensin receptor blockers; DI, Diuretics; CCB, calcium channel blockers; ACEI, angiotensin-converting enzyme inhibitor; BB, βadrenergic receptor blockers; nonRASI, non-renin-angiotensin system (RAS) inhibitors.
