# Supplementary material for: Comparative efficacy of different antihypertensive drug classes for stroke prevention: A network meta-analysis of randomized controlled trials
Source: PLoS One. 2025 Feb 21;20(2):e0313309. doi: 10.1371/journal.pone.0313309 (PMC11845040; doi:10.1371/journal.pone.0313309)
Supplement: S4 Table — (DOCX) [file pone.0313309.s005.docx]

**S4 Table. Node-splitting results for all-cause mortality in the overall population.**

| \| **Comparison** \| **NMA  mean difference** \| **Direct  mean difference** \| **Indirect  mean difference** \| ***p-value*** \| \| --- \| --- \| --- \| --- \| --- \| \| ACEI vs.ARB \| 0.033 (-0.030, 0.11) \| 0.072 (0.0049, 0.13) \| 0.053 (0.0063, 0.10) \| 0.430425 \| \| ACEI vs.ARB+ACEI \| 0.029 (-0.055, 0.11) \| 0.12 (-0.23, 0.48) \| 0.051 (-0.025, 0.13) \| 0.626925 \| \| ACEI vs.BB \| 0.40 (0.11, 0.73) \| 0.061 (-0.019, 0.15) \| 0.082 (0.0035, 0.17) \| 0.031075 \| \| ACEI vs.CCB \| -0.024 (-0.11, 0.073) \| 0.056 (-0.026, 0.14) \| 0.022 (-0.036, 0.086) \| 0.1991 \| \| ACEI vs.Conventional therapy \| -0.029 (-0.20, 0.13) \| -0.032 (-0.14, 0.081) \| -0.026 (-0.12, 0.068) \| 0.96725 \| \| ACEI vs.DI \| 0.014 (-0.077, 0.11) \| -0.024 (-0.13, 0.084) \| 0.012 (-0.056, 0.082) \| 0.57955 \| \| ACEI vs.nonRASI \| 0.21 (-0.25, 0.70) \| -0.085 (-0.42, 0.26) \| -0.0018 (-0.28, 0.29) \| 0.300075 \| \| ACEI vs.Placebo \| 0.11 (0.045, 0.17) \| 0.087 (0.020, 0.16) \| 0.097 (0.054, 0.14) \| 0.697775 \| \| ACEI+CCB vs.ACEI+DI \| 0.10 (-0.092, 0.30) \| 0.31 (0.022, 0.61) \| 0.17 (0.0043, 0.33) \| 0.237375 \| \| ACEI+CCB vs.CCB \| 0.49 (0.17, 0.82) \| 0.19 (-0.025, 0.40) \| 0.28 (0.10, 0.45) \| 0.1179 \| \| ACEI+CCB vs.Placebo \| 0.25 (-0.27, 0.84) \| 0.36 (0.18, 0.55) \| 0.35 (0.18, 0.53) \| 0.70375 \| \| ACEI+DI vs.Placebo \| 0.17 (0.057, 0.27) \| 0.39 (0.040, 0.74) \| 0.19 (0.083, 0.29) \| 0.231325 \| \| ARB vs.ARB+ACEI \| 0.024 (-0.062, 0.11) \| 0.019 (-0.44, 0.49) \| -0.0012 (-0.079, 0.076) \| 0.98595 \| \| ARB vs.BB \| 0.12 (-0.041, 0.28) \| -0.0014 (-0.091, 0.094) \| 0.030 (-0.050, 0.11) \| 0.19785 \| \| ARB vs.CCB \| 0.028 (-0.10, 0.16) \| -0.049 (-0.12, 0.021) \| -0.031 (-0.092, 0.033) \| 0.3111 \| \| ARB vs.nonRASI \| -0.15 (-0.50, 0.19) \| 0.19 (-0.31, 0.67) \| -0.055 (-0.33, 0.24) \| 0.285525 \| \| ARB vs.Placebo \| 0.018 (-0.030, 0.069) \| 0.089 (0.020, 0.16) \| 0.044 (0.0010, 0.088) \| 0.104575 \| \| ARB+ACEI vs.BB \| 0.25 (-0.28, 0.71) \| 0.025 (-0.079, 0.13) \| 0.030 (-0.075, 0.14) \| 0.4136 \| \| BB vs.CCB \| -0.033 (-0.16, 0.088) \| -0.078 (-0.18, 0.036) \| -0.060 (-0.14, 0.022) \| 0.595975 \| \| BB vs.DI \| -0.080 (-0.25, 0.097) \| -0.071 (-0.19, 0.040) \| -0.070 (-0.17, 0.020) \| 0.92255 \| \| BB vs.Placebo \| 0.072 (-0.049, 0.19) \| -0.031 (-0.15, 0.071) \| 0.014 (-0.066, 0.091) \| 0.1999 \| \| CCB vs.Conventional therapy \| -0.025 (-0.13, 0.081) \| -0.088 (-0.26, 0.070) \| -0.048 (-0.13, 0.038) \| 0.511625 \| \| CCB vs.DI \| 0.0092 (-0.098, 0.099) \| -0.022 (-0.14, 0.093) \| -0.010 (-0.083, 0.058) \| 0.683025 \| \| CCB vs.Placebo \| 0.10 (-0.030, 0.23) \| 0.071 (0.0011, 0.13) \| 0.075 (0.016, 0.13) \| 0.65885 \| \| Conventional therapy vs.Placebo \| 0.15 (0.0074, 0.30) \| 0.11 (-0.0026, 0.23) \| 0.12 (0.030, 0.21) \| 0.642525 \| \| DI vs.Placebo \| 0.090 (-0.028, 0.20) \| 0.079 (-0.010, 0.18) \| 0.084 (0.017, 0.16) \| 0.885375 \| |  |  |  |  |
| --- | --- | --- | --- | --- | --- | --- | --- | --- | --- | --- | --- | --- | --- | --- | --- | --- | --- | --- | --- | --- | --- | --- | --- | --- | --- | --- | --- | --- | --- | --- | --- | --- | --- | --- | --- | --- | --- | --- | --- | --- | --- | --- | --- | --- | --- | --- | --- | --- | --- | --- | --- | --- | --- | --- | --- | --- | --- | --- | --- | --- | --- | --- | --- | --- | --- | --- | --- | --- | --- | --- | --- | --- | --- | --- | --- | --- | --- | --- | --- | --- | --- | --- | --- | --- | --- | --- | --- | --- | --- | --- | --- | --- | --- | --- | --- | --- | --- | --- | --- | --- | --- | --- | --- | --- | --- | --- | --- | --- | --- | --- | --- | --- | --- | --- | --- | --- | --- | --- | --- | --- | --- | --- | --- | --- | --- | --- | --- | --- | --- | --- | --- | --- | --- | --- | --- | --- | --- | --- | --- |

Abbreviations: ARB, angiotensin receptor blockers; DI, Diuretics; CCB, calcium channel blockers; ACEI, angiotensin-converting enzyme inhibitor; BB, βadrenergic receptor blockers; nonRASI, non-renin-angiotensin system (RAS) inhibitors.
