# Supplementary material for: Comparative efficacy of different antihypertensive drug classes for stroke prevention: A network meta-analysis of randomized controlled trials
Source: PLoS One. 2025 Feb 21;20(2):e0313309. doi: 10.1371/journal.pone.0313309 (PMC11845040; doi:10.1371/journal.pone.0313309)
Supplement: S5 Table — (DOCX) [file pone.0313309.s006.docx]

**S5 Table. Node-splitting results for cardiovascular mortality** **in the overall population.**

| **Comparison** | **NMA  mean difference** | **Direct  mean difference** | **Indirect  mean difference** | ***p-value*** |
| --- | --- | --- | --- | --- |
| ACEI vs.ARB | 0.050 (-0.030, 0.14) | 0.11 (0.021, 0.20) | 0.075 (0.018, 0.14) | 0.33175 |
| ACEI vs.BB | 0.26 (-0.38, 0.79) | 0.072 (-0.033, 0.19) | 0.077 (-0.025, 0.19) | 0.5903 |
| ACEI vs.CCB | -0.032 (-0.15, 0.087) | 0.024 (-0.086, 0.13) | -0.0016 (-0.078, 0.076) | 0.503025 |
| ACEI vs.Conventional therapy | 0.049 (-0.12, 0.22) | -0.15 (-0.30, 0.00022) | -0.053 (-0.17, 0.056) | 0.0892 |
| ACEI vs.DI | -0.027 (-0.15, 0.11) | -0.14 (-0.29, 0.011) | -0.056 (-0.15, 0.034) | 0.246475 |
| ACEI vs.nonRASI | 0.52 (-0.19, 1.3) | -0.043 (-0.58, 0.50) | 0.17 (-0.25, 0.62) | 0.2226 |
| ACEI vs.Placebo | 0.14 (0.053, 0.21) | 0.14 (0.067, 0.24) | 0.14 (0.086, 0.20) | 0.9032 |
| ACEI+CCB vs.ACEI+DI | 0.22 (-0.050, 0.50) | 0.17 (-0.20, 0.58) | 0.19 (-0.019, 0.42) | 0.829675 |
| ACEI+CCB vs.CCB | 0.30 (-0.11, 0.75) | 0.27 (-0.0096, 0.56) | 0.27 (0.047, 0.52) | 0.92 |
| ACEI+CCB vs.Placebo | 0.24 (-0.49, 0.98) | 0.44 (0.19, 0.69) | 0.42 (0.19, 0.65) | 0.6125 |
| ACEI+DI vs.Placebo | 0.23 (0.083, 0.37) | 0.18 (-0.27, 0.62) | 0.23 (0.085, 0.37) | 0.83 |
| ARB vs.ARB+ACEI | 0.021 (-0.072, 0.11) | 0.13 (-0.53, 0.85) | -0.016 (-0.11, 0.077) | 0.768925 |
| ARB vs.BB | 0.14 (-0.077, 0.35) | -0.044 (-0.16, 0.075) | 0.0029 (-0.10, 0.11) | 0.146275 |
| ARB vs.CCB | -0.13 (-0.35, 0.087) | -0.069 (-0.16, 0.023) | -0.076 (-0.16, 0.0044) | 0.6126 |
| ARB vs.nonRASI | -0.11 (-0.65, 0.42) | 0.46 (-0.27, 1.2) | 0.099 (-0.33, 0.54) | 0.194375 |
| ARB vs.Placebo | 0.041 (-0.023, 0.10) | 0.12 (0.026, 0.20) | 0.066 (0.012, 0.12) | 0.16325 |
| ARB+ACEI vs.BB | 0.0094 (-0.80, 0.82) | 0.018 (-0.11, 0.15) | 0.018 (-0.11, 0.15) | 0.9788 |
| BB vs.CCB | -0.0061 (-0.17, 0.15) | -0.14 (-0.28, -0.0053) | -0.078 (-0.19, 0.026) | 0.199675 |
| BB vs.DI | -0.23 (-0.48, 0.014) | -0.10 (-0.26, 0.040) | -0.13 (-0.27, -0.010) | 0.38515 |
| BB vs.Placebo | 0.088 (-0.065, 0.24) | 0.045 (-0.092, 0.19) | 0.064 (-0.038, 0.17) | 0.699575 |
| CCB vs.Conventional therapy | -0.045 (-0.19, 0.091) | -0.055 (-0.24, 0.14) | -0.051 (-0.16, 0.054) | 0.9486 |
| CCB vs.DI | -0.029 (-0.17, 0.11) | -0.099 (-0.25, 0.062) | -0.055 (-0.16, 0.041) | 0.5029 |
| CCB vs.Placebo | 0.15 (-0.014, 0.32) | 0.14 (0.042, 0.23) | 0.14 (0.065, 0.22) | 0.875475 |
| Conventional therapy vs.Placebo | 0.32 (0.12, 0.53) | 0.14 (0.013, 0.28) | 0.19 (0.086, 0.31) | 0.132675 |
| DI vs.Placebo | 0.23 (0.077, 0.38) | 0.17 (0.053, 0.30) | 0.20 (0.11, 0.30) | 0.5723 |

Abbreviations: ARB, angiotensin receptor blockers; DI, Diuretics; CCB, calcium channel blockers; ACEI, angiotensin-converting enzyme inhibitor; BB, βadrenergic receptor blockers; nonRASI, non-renin-angiotensin system (RAS) inhibitors.
