# Supplementary material for: Comparative efficacy of different antihypertensive drug classes for stroke prevention: A network meta-analysis of randomized controlled trials
Source: PLoS One. 2025 Feb 21;20(2):e0313309. doi: 10.1371/journal.pone.0313309 (PMC11845040; doi:10.1371/journal.pone.0313309)
Supplement: S6 Table — (DOCX) [file pone.0313309.s007.docx]

**S6 Table. Node-splitting results for stroke in hypertensive patients.**

| **Comparison** | **NMA  mean difference** | **Direct  mean difference** | **Indirect  mean difference** | ***p-value*** |
| --- | --- | --- | --- | --- |
| ACEI vs.ARB | 0.21 (-0.38, 0.83) | -0.027 (-0.24, 0.18) | -0.0083 (-0.20, 0.19) | 0.4603 |
| ACEI vs.BB | -0.14 (-0.70, 0.40) | 0.20 (-0.039, 0.45) | 0.14 (-0.077, 0.36) | 0.259375 |
| ACEI vs.CCB | -0.049 (-0.27, 0.19) | -0.13 (-0.39, 0.11) | -0.090 (-0.24, 0.065) | 0.609725 |
| ACEI vs.Conventional therapy | 0.064 (-0.20, 0.33) | -0.20 (-0.46, 0.060) | -0.065 (-0.25, 0.11) | 0.152275 |
| ACEI vs.DI | -0.15 (-0.42, 0.099) | -0.13 (-0.39, 0.13) | -0.14 (-0.32, 0.034) | 0.904375 |
| ACEI vs.nonRASI | -0.72 (-2.8, 0.99) | 0.34 (-0.052, 0.74) | 0.29 (-0.094, 0.67) | 0.2263 |
| ACEI vs.Placebo | 0.12 (-0.21, 0.45) | 0.37 (0.19, 0.58) | 0.31 (0.15, 0.49) | 0.170325 |
| ACEI+CCB vs.ACEI+DI | 0.17 (-0.24, 0.58) | 0.36 (-0.22, 0.96) | 0.23 (-0.097, 0.56) | 0.600575 |
| ACEI+CCB vs.CCB | 0.50 (-0.12, 1.1) | 0.21 (-0.24, 0.65) | 0.30 (-0.056, 0.66) | 0.4582 |
| ACEI+CCB vs.Placebo | 0.62 (-0.16, 1.5) | 0.73 (0.35, 1.1) | 0.71 (0.36, 1.1) | 0.822625 |
| ACEI+DI vs.Placebo | 0.45 (0.15, 0.74) | 0.64 (-0.0067, 1.3) | 0.48 (0.21, 0.75) | 0.6072 |
| ARB vs.BB | 0.29 (-0.069, 0.65) | 0.071 (-0.18, 0.33) | 0.15 (-0.057, 0.35) | 0.3101 |
| ARB vs.CCB | 0.034 (-0.17, 0.23) | -0.21 (-0.44, 0.011) | -0.081 (-0.24, 0.071) | 0.1089 |
| ARB vs.nonRASI | 0.34 (-0.0035, 0.68) | -0.73 (-2.8, 1.1) | 0.30 (-0.044, 0.63) | 0.261325 |
| BB vs.CCB | -0.19 (-0.52, 0.12) | -0.27 (-0.53, -0.011) | -0.23 (-0.42, -0.041) | 0.6935 |
| BB vs.DI | -0.44 (-0.83, -0.059) | -0.23 (-0.51, 0.049) | -0.28 (-0.50, -0.065) | 0.38455 |
| BB vs.Placebo | 0.23 (-0.076, 0.54) | 0.15 (-0.077, 0.42) | 0.17 (-0.023, 0.38) | 0.707525 |
| CCB vs.Conventional therapy | 0.074 (-0.15, 0.30) | -0.062 (-0.33, 0.19) | 0.025 (-0.15, 0.19) | 0.422375 |
| CCB vs.DI | 0.062 (-0.18, 0.30) | -0.14 (-0.38, 0.086) | -0.048 (-0.21, 0.11) | 0.216225 |
| CCB vs.Placebo | 0.54 (0.30, 0.80) | 0.35 (0.19, 0.51) | 0.40 (0.27, 0.55) | 0.186775 |
| Conventional therapy vs.Placebo | 0.55 (0.29, 0.82) | 0.25 (0.040, 0.48) | 0.38 (0.21, 0.57) | 0.0902 |
| DI vs.Placebo | 0.55 (0.33, 0.79) | 0.35 (0.099, 0.61) | 0.45 (0.29, 0.63) | 0.24265 |

Abbreviations: ARB, angiotensin receptor blockers; DI, Diuretics; CCB, calcium channel blockers; ACEI, angiotensin-converting enzyme inhibitor; BB, βadrenergic receptor blockers; nonRASI, non-renin-angiotensin system (RAS) inhibitors.
