# Supplementary material for: Comparative efficacy of different antihypertensive drug classes for stroke prevention: A network meta-analysis of randomized controlled trials
Source: PLoS One. 2025 Feb 21;20(2):e0313309. doi: 10.1371/journal.pone.0313309 (PMC11845040; doi:10.1371/journal.pone.0313309)
Supplement: S7 Table — (DOCX) [file pone.0313309.s008.docx]

**S7 Table. Node-splitting results for all-cause mortality in hypertensive patients***.*

| \| **Comparison** \| **NMA  mean difference** \| **Direct  mean difference** \| **Indirect  mean difference** \| ***p-value*** \| \| --- \| --- \| --- \| --- \| --- \| \| ACEI vs.ARB \| -0.082 (-0.76, 0.60) \| 0.045 (-0.060, 0.17) \| 0.039 (-0.070, 0.15) \| 0.722075 \| \| ACEI vs.BB \| 0.43 (0.099, 0.75) \| 0.080 (-0.025, 0.20) \| 0.12 (0.018, 0.24) \| 0.05615 \| \| ACEI vs.CCB \| -0.027 (-0.11, 0.083) \| 0.12 (-0.021, 0.26) \| 0.021 (-0.051, 0.11) \| 0.1061 \| \| ACEI vs.Conventional therapy \| -0.032 (-0.22, 0.15) \| -0.0052 (-0.14, 0.15) \| -0.017 (-0.11, 0.093) \| 0.81635 \| \| ACEI vs.DI \| 0.014 (-0.083, 0.13) \| 0.029 (-0.095, 0.17) \| 0.024 (-0.053, 0.11) \| 0.85835 \| \| ACEI vs.nonRASI \| 0.24 (-0.24, 0.71) \| -0.13 (-0.49, 0.24) \| 0.0063 (-0.28, 0.29) \| 0.234825 \| \| ACEI vs.Placebo \| 0.14 (-0.033, 0.29) \| 0.13 (0.029, 0.27) \| 0.13 (0.049, 0.22) \| 0.93335 \| \| ACEI+CCB vs.ACEI+DI \| 0.10 (-0.10, 0.31) \| 0.33 (0.0076, 0.65) \| 0.16 (-0.0041, 0.33) \| 0.23545 \| \| ACEI+CCB vs.CCB \| 0.47 (0.15, 0.81) \| 0.17 (-0.078, 0.40) \| 0.27 (0.091, 0.46) \| 0.130125 \| \| ACEI+CCB vs.Placebo \| 0.26 (-0.26, 0.78) \| 0.40 (0.20, 0.60) \| 0.38 (0.20, 0.57) \| 0.6324 \| \| ACEI+DI vs.Placebo \| 0.19 (0.028, 0.35) \| 0.41 (0.053, 0.78) \| 0.22 (0.084, 0.36) \| 0.244975 \| \| ARB vs.BB \| 0.12 (-0.052, 0.29) \| 0.055 (-0.074, 0.21) \| 0.080 (-0.024, 0.19) \| 0.56715 \| \| ARB vs.CCB \| 0.033 (-0.11, 0.17) \| -0.049 (-0.18, 0.075) \| -0.017 (-0.11, 0.072) \| 0.3692 \| \| ARB vs.nonRASI \| -0.17 (-0.51, 0.19) \| 0.21 (-0.30, 0.73) \| -0.032 (-0.30, 0.24) \| 0.232725 \| \| ARB vs.Placebo \| 0.047 (-0.074, 0.18) \| 0.14 (0.011, 0.27) \| 0.092 (-0.0017, 0.18) \| 0.282325 \| \| BB vs.CCB \| -0.033 (-0.16, 0.080) \| -0.15 (-0.26, -0.029) \| -0.097 (-0.19, -0.0091) \| 0.192975 \| \| BB vs.DI \| -0.073 (-0.26, 0.12) \| -0.11 (-0.28, 0.022) \| -0.094 (-0.21, 0.0055) \| 0.745825 \| \| BB vs.Placebo \| -0.0072 (-0.17, 0.16) \| 0.020 (-0.13, 0.15) \| 0.011 (-0.088, 0.10) \| 0.8017 \| \| CCB vs.Conventional therapy \| -0.026 (-0.14, 0.090) \| -0.059 (-0.24, 0.13) \| -0.039 (-0.13, 0.048) \| 0.76085 \| \| CCB vs.DI \| 0.0079 (-0.11, 0.10) \| 0.0055 (-0.12, 0.13) \| 0.0025 (-0.075, 0.078) \| 0.9852 \| \| Conventional therapy vs.Placebo \| 0.16 (0.0040, 0.31) \| 0.15 (0.015, 0.28) \| 0.15 (0.053, 0.24) \| 0.920675 \| \| DI vs.Placebo \| 0.091 (-0.030, 0.21) \| 0.13 (0.0087, 0.28) \| 0.10 (0.030, 0.19) \| 0.6273 \| |
| --- | --- | --- | --- | --- | --- | --- | --- | --- | --- | --- | --- | --- | --- | --- | --- | --- | --- | --- | --- | --- | --- | --- | --- | --- | --- | --- | --- | --- | --- | --- | --- | --- | --- | --- | --- | --- | --- | --- | --- | --- | --- | --- | --- | --- | --- | --- | --- | --- | --- | --- | --- | --- | --- | --- | --- | --- | --- | --- | --- | --- | --- | --- | --- | --- | --- | --- | --- | --- | --- | --- | --- | --- | --- | --- | --- | --- | --- | --- | --- | --- | --- | --- | --- | --- | --- | --- | --- | --- | --- | --- | --- | --- | --- | --- | --- | --- | --- | --- | --- | --- | --- | --- | --- | --- | --- | --- | --- | --- | --- | --- | --- | --- | --- | --- | --- |

Abbreviations: ARB, angiotensin receptor blockers; DI, Diuretics; CCB, calcium channel blockers; ACEI, angiotensin-converting enzyme inhibitor; BB, βadrenergic receptor blockers; nonRASI, non-renin-angiotensin system (RAS) inhibitors.
