# Supplementary material for: Comparative efficacy of different antihypertensive drug classes for stroke prevention: A network meta-analysis of randomized controlled trials
Source: PLoS One. 2025 Feb 21;20(2):e0313309. doi: 10.1371/journal.pone.0313309 (PMC11845040; doi:10.1371/journal.pone.0313309)
Supplement: S8 Table — (DOCX) [file pone.0313309.s009.docx]

**S8 Table. Node-splitting results for cardiovascular mortality** **in hypertensive patients.**

| **Comparison** | **NMA  mean difference** | **Direct  mean difference** | **Indirect  mean difference** | ***p-value*** |
| --- | --- | --- | --- | --- |
| ACEI vs.ARB | 0.040 (-1.8, 2.9) | 0.11 (-0.069, 0.30) | 0.099 (-0.070, 0.29) | 0.95155 |
| ACEI vs.BB | 0.24 (-0.39, 0.87) | 0.15 (-0.0063, 0.34) | 0.15 (0.0056, 0.33) | 0.78725 |
| ACEI vs.CCB | -0.022 (-0.15, 0.12) | 0.20 (-0.047, 0.43) | 0.027 (-0.077, 0.15) | 0.11665 |
| ACEI vs.Conventional therapy | 0.046 (-0.13, 0.24) | -0.10 (-0.29, 0.093) | -0.017 (-0.15, 0.12) | 0.242675 |
| ACEI vs.DI | -0.028 (-0.18, 0.13) | 0.027 (-0.20, 0.27) | -0.019 (-0.13, 0.11) | 0.711675 |
| ACEI vs.nonRASI | 0.48 (-0.23, 1.2) | -0.018 (-0.61, 0.53) | 0.19 (-0.22, 0.63) | 0.29785 |
| ACEI vs.Placebo | -0.16 (-0.80, 0.46) | 0.25 (0.13, 0.42) | 0.24 (0.11, 0.38) | 0.214675 |
| ACEI+CCB vs.ACEI+DI | 0.23 (-0.069, 0.51) | 0.18 (-0.24, 0.61) | 0.21 (-0.016, 0.44) | 0.85805 |
| ACEI+CCB vs.CCB | 0.31 (-0.096, 0.75) | 0.21 (-0.13, 0.54) | 0.25 (-0.0010, 0.52) | 0.69595 |
| ACEI+CCB vs.Placebo | 0.24 (-0.43, 0.98) | 0.50 (0.22, 0.78) | 0.46 (0.22, 0.72) | 0.52775 |
| ACEI+DI vs.Placebo | 0.27 (0.053, 0.47) | 0.22 (-0.23, 0.68) | 0.25 (0.065, 0.44) | 0.862125 |
| ARB vs.BB | 0.14 (-0.075, 0.36) | -0.040 (-0.23, 0.18) | 0.053 (-0.10, 0.21) | 0.23665 |
| ARB vs.CCB | -0.15 (-0.38, 0.085) | 0.0029 (-0.21, 0.20) | -0.072 (-0.23, 0.072) | 0.35315 |
| ARB vs.nonRASI | -0.12 (-0.68, 0.47) | 0.44 (-0.32, 1.2) | 0.091 (-0.32, 0.54) | 0.277425 |
| ARB vs.Placebo | 0.091 (-0.15, 0.34) | 0.19 (-0.025, 0.40) | 0.14 (-0.023, 0.30) | 0.5596 |
| BB vs.DI | -0.22 (-0.48, 0.034) | -0.13 (-0.35, 0.045) | -0.17 (-0.33, -0.025) | 0.55325 |
| BB vs.Placebo | -0.0025 (-0.20, 0.22) | 0.15 (-0.045, 0.33) | 0.083 (-0.057, 0.22) | 0.277725 |
| CCB vs.Conventional therapy | -0.052 (-0.20, 0.10) | -0.025 (-0.24, 0.19) | -0.044 (-0.17, 0.069) | 0.826525 |
| CCB vs.DI | -0.033 (-0.22, 0.13) | -0.058 (-0.23, 0.13) | -0.045 (-0.16, 0.062) | 0.849375 |
| CCB vs.Placebo | 0.33 (0.10, 0.57) | 0.18 (0.050, 0.31) | 0.21 (0.10, 0.33) | 0.2424 |
| Conventional therapy vs.Placebo | 0.32 (0.11, 0.54) | 0.22 (0.039, 0.40) | 0.25 (0.13, 0.40) | 0.446575 |
| DI vs.Placebo | 0.23 (0.080, 0.38) | 0.32 (0.13, 0.54) | 0.26 (0.14, 0.38) | 0.506175 |

Abbreviations: ARB, angiotensin receptor blockers; DI, Diuretics; CCB, calcium channel blockers; ACEI, angiotensin-converting enzyme inhibitor; BB, βadrenergic receptor blockers; nonRASI, non-renin-angiotensin system (RAS) inhibitors.
