# Supplementary material for: Comparative efficacy of different antihypertensive drug classes for stroke prevention: A network meta-analysis of randomized controlled trials
Source: PLoS One. 2025 Feb 21;20(2):e0313309. doi: 10.1371/journal.pone.0313309 (PMC11845040; doi:10.1371/journal.pone.0313309)
Supplement: S9 Table — (DOCX) [file pone.0313309.s010.docx]

**S9 Table. Node-splitting results for subgroup analysis of stroke in hypertensive patients.**

| **Comparison** | **NMA  mean difference** | **Direct  mean difference** | **Indirect  mean difference** | ***p-value*** |
| --- | --- | --- | --- | --- |
| ACEI vs.ARB | 0.20 (-0.42, 0.82) | -0.025 (-0.23, 0.19) | -0.0022 (-0.20, 0.20) | 0.48815 |
| ACEI vs.BB | -0.15 (-0.71, 0.39) | 0.23 (-0.027, 0.50) | 0.16 (-0.073, 0.40) | 0.212925 |
| ACEI vs.CCB | -0.048 (-0.27, 0.19) | -0.10 (-0.39, 0.17) | -0.086 (-0.25, 0.083) | 0.7457 |
| ACEI vs.Conventional therapy | 0.063 (-0.19, 0.32) | -0.29 (-0.59, 0.011) | -0.079 (-0.28, 0.11) | 0.07805 |
| ACEI vs. DI(TL) | -0.13 (-0.50, 0.24) | 0.024 (-0.34, 0.40) | -0.048 (-0.28, 0.20) | 0.53795 |
| ACEI vs. DI(TT) | -0.18 (-0.58, 0.21) | -0.23 (-0.52, 0.062) | -0.21 (-0.45, 0.011) | 0.820725 |
| ACEI vs.nonRASI | -0.71 (-2.9, 0.99) | 0.35 (-0.048, 0.76) | 0.29 (-0.094, 0.67) | 0.24295 |
| ACEI vs.Placebo | 0.11 (-0.22, 0.45) | 0.38 (0.20, 0.58) | 0.32 (0.15, 0.49) | 0.168 |
| ACEI+CCB vs.ACEI+DI | 0.17 (-0.25, 0.59) | 0.36 (-0.22, 0.96) | 0.23 (-0.10, 0.57) | 0.60415 |
| ACEI+CCB vs.CCB | 0.49 (-0.12, 1.1) | 0.20 (-0.25, 0.66) | 0.31 (-0.056, 0.67) | 0.4567 |
| ACEI+CCB vs.Placebo | 0.62 (-0.17, 1.5) | 0.73 (0.35, 1.1) | 0.71 (0.36, 1.1) | 0.815875 |
| ACEI+DI vs.Placebo | 0.45 (0.15, 0.75) | 0.63 (-0.0080, 1.3) | 0.48 (0.21, 0.75) | 0.611425 |
| ARB vs.BB | 0.29 (-0.078, 0.66) | 0.085 (-0.20, 0.37) | 0.17 (-0.063, 0.38) | 0.361875 |
| ARB vs.CCB | 0.038 (-0.17, 0.24) | -0.25 (-0.48, -0.00016) | -0.084 (-0.24, 0.076) | 0.08405 |
| ARB vs.nonRASI | 0.34 (-0.0069, 0.69) | -0.69 (-2.9, 0.99) | 0.29 (-0.047, 0.63) | 0.237425 |
| BB vs.CCB | -0.36 (-0.99, 0.23) | -0.25 (-0.49, 0.0062) | -0.25 (-0.47, -0.021) | 0.715875 |
| BB vs. CCB(V) | -0.13 (-0.52, 0.26) | -0.13 (-0.64, 0.35) | -0.13 (-0.43, 0.16) | 0.981325 |
| BB vs. DI(TT) | -0.44 (-0.84, -0.060) | -0.35 (-0.70, 0.017) | -0.38 (-0.64, -0.12) | 0.712625 |
| ACEI vs.Conventional therapy | 0.063 (-0.19, 0.32) | -0.29 (-0.59, 0.011) | -0.079 (-0.28, 0.11) | 0.07805 |
| ACEI vs. DI(TL) | -0.13 (-0.50, 0.24) | 0.024 (-0.34, 0.40) | -0.048 (-0.28, 0.20) | 0.53795 |
| ACEI vs. DI(TT) | -0.18 (-0.58, 0.21) | -0.23 (-0.52, 0.062) | -0.21 (-0.45, 0.011) | 0.820725 |

Abbreviations: ARB, angiotensin receptor blockers; DI, Diuretics; DI(TL), thiazide-like diuretics; DI(TT), thiazide-type diuretics; CCB, calcium channel blockers; CCB(V), calcium channel blockers (verapamil); ACEI, angiotensin-converting enzyme inhibitor; BB, βadrenergic receptor blockers; nonRASI, non-renin-angiotensin system (RAS) inhibitors.
