# Supplementary material for: Comparative efficacy of different antihypertensive drug classes for stroke prevention: A network meta-analysis of randomized controlled trials
Source: PLoS One. 2025 Feb 21;20(2):e0313309. doi: 10.1371/journal.pone.0313309 (PMC11845040; doi:10.1371/journal.pone.0313309)
Supplement: S10 Table — (DOCX) [file pone.0313309.s011.docx]

**S10 Table. Node-splitting results for subgroup analysis of all-cause mortality in hypertensive patients***.*

| \| **Comparison** \| **NMA  mean difference** \| **Direct  mean difference** \| **Indirect  mean difference** \| ***p-value*** \| \| --- \| --- \| --- \| --- \| --- \| \| ACEI vs.ARB \| -0.071 (-0.76, 0.62) \| 0.053 (-0.052, 0.17) \| 0.049 (-0.054, 0.16) \| 0.725725 \| \| ACEI vs.BB \| 0.41 (0.085, 0.74) \| 0.13 (0.0080, 0.25) \| 0.17 (0.057, 0.29) \| 0.111175 \| \| ACEI vs.CCB \| -0.025 (-0.11, 0.091) \| 0.086 (-0.073, 0.23) \| 0.0022 (-0.070, 0.097) \| 0.251075 \| \| ACEI vs.Conventional therapy \| -0.035 (-0.23, 0.16) \| 0.037 (-0.13, 0.21) \| 0.0075 (-0.10, 0.12) \| 0.5678 \| \| ACEI vs. DI(TL) \| -0.0027 (-0.14, 0.13) \| -0.037 (-0.23, 0.16) \| 0.0036 (-0.096, 0.10) \| 0.7702 \| \| ACEI vs. DI(TT) \| 0.078 (-0.12, 0.28) \| 0.032 (-0.10, 0.19) \| 0.049 (-0.062, 0.17) \| 0.71745 \| \| ACEI vs.nonRASI \| 0.23 (-0.25, 0.78) \| -0.12 (-0.47, 0.24) \| 0.0091 (-0.28, 0.29) \| 0.270275 \| \| ACEI vs.Placebo \| 0.14 (-0.040, 0.29) \| 0.15 (0.044, 0.29) \| 0.14 (0.061, 0.24) \| 0.881425 \| \| ACEI+CCB vs.ACEIandDI \| 0.10 (-0.099, 0.31) \| 0.35 (0.024, 0.67) \| 0.17 (-0.00099, 0.34) \| 0.1797 \| \| ACEI+CCB vs.CCB \| 0.48 (0.17, 0.83) \| 0.12 (-0.10, 0.37) \| 0.25 (0.062, 0.45) \| 0.0852 \| \| ACEI+CCB vs.Placebo \| 0.24 (-0.27, 0.82) \| 0.42 (0.22, 0.63) \| 0.40 (0.20, 0.58) \| 0.580125 \| \| ACEI+DI vs.Placebo \| 0.19 (0.031, 0.34) \| 0.44 (0.097, 0.81) \| 0.23 (0.085, 0.36) \| 0.177225 \| \| ARB vs.BB \| 0.13 (-0.054, 0.29) \| 0.12 (-0.034, 0.27) \| 0.12 (0.0084, 0.23) \| 0.930175 \| \| ARB vs.CCB \| 0.033 (-0.10, 0.17) \| -0.12 (-0.25, 0.022) \| -0.047 (-0.15, 0.055) \| 0.122375 \| \| ARB vs.nonRASI \| -0.15 (-0.50, 0.20) \| 0.17 (-0.31, 0.69) \| -0.043 (-0.33, 0.24) \| 0.2674 \| \| ARB vs.Placebo \| 0.046 (-0.072, 0.18) \| 0.16 (0.015, 0.31) \| 0.096 (0.0073, 0.19) \| 0.2454 \| \| BB vs.CCB \| -0.17 (-0.52, 0.19) \| -0.15 (-0.27, -0.025) \| -0.16 (-0.27, -0.050) \| 0.92625 \| \| BB vs. CCB(V) \| -0.020 (-0.17, 0.13) \| -0.12 (-0.36, 0.11) \| -0.041 (-0.16, 0.072) \| 0.4459 \| \| BB vs. DI(TT) \| -0.075 (-0.26, 0.12) \| -0.16 (-0.37, 0.045) \| -0.12 (-0.24, 0.0063) \| 0.55255 \| \| BB vs.Placebo \| 0.00033 (-0.16, 0.16) \| -0.044 (-0.20, 0.10) \| -0.021 (-0.12, 0.081) \| 0.673475 \| \| CCB vs.Conventional therapy \| 0.010 (-0.17, 0.20) \| 0.0094 (-0.16, 0.17) \| 0.0054 (-0.11, 0.12) \| 0.9758 \| \| CCB vs. DI(TL) \| 0.0098 (-0.14, 0.12) \| 0.0079 (-0.26, 0.28) \| 0.0033 (-0.11, 0.085) \| 0.978375 \| \| CCB vs. DI(TT) \| -0.0070 (-0.25, 0.23) \| 0.062 (-0.069, 0.19) \| 0.046 (-0.068, 0.16) \| 0.5818 \| \| CCB vs.Placebo \| 0.31 (0.11, 0.51) \| 0.11 (0.0028, 0.20) \| 0.14 (0.057, 0.23) \| 0.059275 \| \| CCB(V) vs.Conventional therapy \| -0.075 (-0.26, 0.12) \| -0.17 (-0.38, 0.040) \| -0.11 (-0.25, 0.019) \| 0.4654 \| \| CCB(V) vs. DI(TL) \| -0.19 (-1.8, 1.1) \| -0.11 (-0.29, 0.049) \| -0.12 (-0.29, 0.036) \| 0.9017 \| \| Conventional therapy vs.Placebo \| 0.15 (0.0063, 0.32) \| 0.12 (-0.025, 0.28) \| 0.14 (0.035, 0.25) \| 0.74155 \| \| DI(TL) vs.Placebo \| 0.11 (-0.14, 0.36) \| 0.15 (0.031, 0.31) \| 0.14 (0.037, 0.26) \| 0.7606 \| \| DI(TT) vs.Placebo \| 0.088 (-0.051, 0.22) \| 0.11 (-0.082, 0.32) \| 0.096 (-0.0063, 0.20) \| 0.822125 \| |
| --- | --- | --- | --- | --- | --- | --- | --- | --- | --- | --- | --- | --- | --- | --- | --- | --- | --- | --- | --- | --- | --- | --- | --- | --- | --- | --- | --- | --- | --- | --- | --- | --- | --- | --- | --- | --- | --- | --- | --- | --- | --- | --- | --- | --- | --- | --- | --- | --- | --- | --- | --- | --- | --- | --- | --- | --- | --- | --- | --- | --- | --- | --- | --- | --- | --- | --- | --- | --- | --- | --- | --- | --- | --- | --- | --- | --- | --- | --- | --- | --- | --- | --- | --- | --- | --- | --- | --- | --- | --- | --- | --- | --- | --- | --- | --- | --- | --- | --- | --- | --- | --- | --- | --- | --- | --- | --- | --- | --- | --- | --- | --- | --- | --- | --- | --- | --- | --- | --- | --- | --- | --- | --- | --- | --- | --- | --- | --- | --- | --- | --- | --- | --- | --- | --- | --- | --- | --- | --- | --- | --- | --- | --- | --- | --- | --- | --- | --- | --- | --- | --- |

Abbreviations: ARB, angiotensin receptor blockers; DI, Diuretics; DI(TL), thiazide-like diuretics; DI(TT), thiazide-type diuretics; CCB, calcium channel blockers; CCB(V), calcium channel blockers (verapamil); ACEI, angiotensin-converting enzyme inhibitor; BB, βadrenergic receptor blockers; nonRASI, non-renin-angiotensin system (RAS) inhibitors.
