# Supplementary material for: Comparative efficacy of different antihypertensive drug classes for stroke prevention: A network meta-analysis of randomized controlled trials
Source: PLoS One. 2025 Feb 21;20(2):e0313309. doi: 10.1371/journal.pone.0313309 (PMC11845040; doi:10.1371/journal.pone.0313309)
Supplement: S11 Table — (DOCX) [file pone.0313309.s012.docx]

**S11 Table. Node-splitting results for subgroup analysis of cardiovascular mortality** **in hypertensive patients.**

| **Comparison** | **NMA  mean difference** | **Direct  mean difference** | **Indirect  mean difference** | ***p-value*** |
| --- | --- | --- | --- | --- |
| ACEI vs.ARB | 0.099 (-2.3, 2.2) | 0.12 (-0.065, 0.30) | 0.12 (-0.047, 0.31) | 0.981925 |
| ACEI vs.BB | 0.26 (-0.34, 0.79) | 0.23 (0.051, 0.40) | 0.23 (0.056, 0.41) | 0.938725 |
| ACEI vs.CCB | -0.022 (-0.14, 0.11) | 0.098 (-0.18, 0.35) | -0.0083 (-0.12, 0.11) | 0.44855 |
| ACEI vs.Conventional therapy | 0.047 (-0.13, 0.24) | -0.0089 (-0.25, 0.21) | 0.029 (-0.10, 0.17) | 0.706425 |
| ACEI vs.DI(TL) | -0.036 (-0.19, 0.12) | 0.091 (-0.19, 0.44) | -0.013 (-0.13, 0.13) | 0.478725 |
| ACEI vs.DI(TT) | -0.00040 (-0.29, 0.28) | -0.055 (-0.27, 0.16) | -0.027 (-0.19, 0.13) | 0.742225 |
| ACEI vs.nonRASI | 0.56 (-0.11, 1.3) | -0.022 (-0.54, 0.55) | 0.20 (-0.24, 0.62) | 0.1930 |
| ACEI vs.Placebo | -0.14 (-0.72, 0.42) | 0.28 (0.14, 0.42) | 0.26 (0.12, 0.39) | 0.1564 |
| ACEI+CCB vs.ACEI+DI | 0.22 (-0.045, 0.50) | 0.20 (-0.19, 0.65) | 0.22 (-0.035, 0.46) | 0.94365 |
| ACEI+CCB vs.CCB | 0.33 (-0.10, 0.74) | 0.17 (-0.16, 0.53) | 0.21 (-0.077, 0.47) | 0.580475 |
| ACEI+CCB vs.Placebo | 0.21 (-0.45, 0.88) | 0.51 (0.21, 0.78) | 0.48 (0.22, 0.74) | 0.43505 |
| ACEI+DI vs.Placebo | 0.26 (0.062, 0.47) | 0.25 (-0.20, 0.70) | 0.26 (0.076, 0.45) | 0.94925 |
| ARB vs.BB | 0.14 (-0.067, 0.36) | 0.059 (-0.19, 0.29) | 0.11 (-0.050, 0.26) | 0.57565 |
| ARB vs.CCB | -0.14 (-0.36, 0.082) | -0.091 (-0.32, 0.12) | -0.13 (-0.29, 0.025) | 0.77975 |
| ARB vs.nonRASI | -0.11 (-0.64, 0.41) | 0.45 (-0.27, 1.3) | 0.076 (-0.36, 0.48) | 0.2541 |
| ARB vs.Placebo | 0.077 (-0.15, 0.32) | 0.17 (-0.047, 0.38) | 0.14 (-0.029, 0.28) | 0.59955 |
| BB vs.CCB | -0.16 (-0.92, 0.53) | -0.23 (-0.40, -0.073) | -0.24 (-0.40, -0.081) | 0.845575 |
| BB vs.CCB(V) | 0.0058 (-0.17, 0.18) | -0.15 (-0.47, 0.18) | -0.033 (-0.19, 0.12) | 0.411175 |
| BB vs.DI(TT) | -0.22 (-0.46, 0.027) | -0.29 (-0.57, -0.022) | -0.26 (-0.43, -0.080) | 0.697275 |
| BB vs.Placebo | 0.0094 (-0.20, 0.21) | 0.069 (-0.15, 0.28) | 0.027 (-0.11, 0.17) | 0.641225 |
| CCB vs.Conventional therapy | 0.036 (-0.18, 0.25) | 0.057 (-0.13, 0.25) | 0.039 (-0.10, 0.17) | 0.83925 |
| CCB vs.DI(TL) | -0.0078 (-0.17, 0.16) | 0.10 (-0.24, 0.42) | -0.0041 (-0.13, 0.12) | 0.56625 |
| CCB vs.DI(TT) | -0.16 (-0.58, 0.21) | 0.0099 (-0.18, 0.18) | -0.016 (-0.18, 0.13) | 0.387075 |
| CCB vs.Placebo | 0.35 (0.11, 0.56) | 0.24 (0.094, 0.38) | 0.26 (0.15, 0.38) | 0.42085 |
| CCB(V) vs.Conventional therapy | -0.072 (-0.33, 0.17) | -0.24 (-0.50, 0.020) | -0.17 (-0.34, 0.021) | 0.351725 |
| CCB(V) vs.DI(TL) | -0.34 (-2.1, 1.2) | -0.20 (-0.41, 0.026) | -0.20 (-0.42, 0.014) | 0.8659 |
| Conventional therapy vs.Placebo | 0.32 (0.10, 0.55) | 0.16 (-0.028, 0.33) | 0.23 (0.10, 0.36) | 0.22805 |
| DI(TL) vs.Placebo | 0.18 (-0.12, 0.47) | 0.29 (0.12, 0.48) | 0.27 (0.12, 0.41) | 0.51975 |
| DI(TT) vs.Placebo | 0.25 (0.067, 0.43) | 0.38 (0.094, 0.66) | 0.28 (0.14, 0.43) | 0.393125 |

Abbreviations: ARB, angiotensin receptor blockers; DI, Diuretics; DI(TL), thiazide-like diuretics; DI(TT), thiazide-type diuretics; CCB, calcium channel blockers; CCB(V), calcium channel blockers (verapamil); ACEI, angiotensin-converting enzyme inhibitor; BB, βadrenergic receptor blockers; nonRASI, non-renin-angiotensin system (RAS) inhibitors.
