# Supplementary material for: Comparative efficacy of different antihypertensive drug classes for stroke prevention: A network meta-analysis of randomized controlled trials
Source: PLoS One. 2025 Feb 21;20(2):e0313309. doi: 10.1371/journal.pone.0313309 (PMC11845040; doi:10.1371/journal.pone.0313309)
Supplement: S12 Table — (DOCX) [file pone.0313309.s013.docx]

**S12 Table. Outcomes of CINeMA evaluation regarding the outcome of stroke in the overall population.**

| **Comparison** | **Number of studies** | **Within-study bias** | **Reporting bias** | **Indirectness** | **Imprecision** | **Heterogeneity** | **Incoherence** | **Confidence rating** |  |
| --- | --- | --- | --- | --- | --- | --- | --- | --- | --- |
| Mixed evidence | | | | | | | |  |  |
| ACEI vs ACEIandBB | 1 | Some concerns | Low risk | No concerns | Major concerns | No concerns | No concerns | Low |  |
| ACEI vs ARB | 5 | No concerns | Low risk | No concerns | Major concerns | No concerns | No concerns | Low |  |
| ACEI vs ARBandACEI | 3 | No concerns | Low risk | No concerns | Major concerns | No concerns | No concerns | Low |  |
| ACEI vs ARBandACEIandBB | 1 | Some concerns | Low risk | No concerns | Major concerns | No concerns | No concerns | Low |  |
| ACEI vs ARBandBB | 1 | Some concerns | Low risk | No concerns | Major concerns | No concerns | No concerns | Low |  |
| ACEI vs BB | 2 | No concerns | Low risk | No concerns | Major concerns | No concerns | No concerns | Low |  |
| ACEI vs CCB | 8 | No concerns | Low risk | No concerns | No concerns | Major concerns | No concerns | Low |  |
| ACEI vs Conventionaltherapy | 2 | No concerns | Low risk | No concerns | Major concerns | No concerns | Major concerns | Very low |  |
| ACEI vs DI | 3 | No concerns | Low risk | No concerns | No concerns | Major concerns | No concerns | Low |  |
| ACEI vs Placebo | 14 | No concerns | Low risk | No concerns | No concerns | Major concerns | No concerns | Low |  |
| ACEI vs nonRASI | 1 | Some concerns | Low risk | No concerns | Major concerns | No concerns | No concerns | Low |  |
| ACEIandBB vs ARBandACEI | 1 | Some concerns | Low risk | No concerns | Major concerns | No concerns | No concerns | Low |  |
| ACEIandBB vs ARBandACEIandBB | 1 | Some concerns | Low risk | No concerns | Major concerns | No concerns | No concerns | Low |  |
| ACEIandBB vs ARBandBB | 1 | Some concerns | Low risk | No concerns | Major concerns | No concerns | No concerns | Low |  |
| ACEIandBB vs BB | 1 | Some concerns | Low risk | No concerns | Major concerns | No concerns | No concerns | Low |  |
| ACEIandCCB vs ACEIandDI | 1 | No concerns | Low risk | No concerns | Major concerns | No concerns | No concerns | Low |  |
| ACEIandCCB vs BBandDI | 1 | Some concerns | Low risk | No concerns | Major concerns | No concerns | No concerns | Low |  |
| ACEIandCCB vs CCB | 1 | No concerns | Low risk | No concerns | Major concerns | No concerns | No concerns | Low |  |
| ACEIandCCB vs Placebo | 1 | No concerns | Low risk | No concerns | No concerns | No concerns | No concerns | High |  |
| ACEIandDI vs Placebo | 3 | No concerns | Low risk | No concerns | No concerns | Major concerns | No concerns | Low |  |
| ARB vs ARBandACEI | 3 | No concerns | Low risk | No concerns | Major concerns | No concerns | No concerns | Low |  |
| ARB vs BB | 1 | No concerns | Low risk | No concerns | Major concerns | No concerns | No concerns | Low |  |
| ARB vs CCB | 7 | No concerns | Low risk | No concerns | Major concerns | No concerns | Major concerns | Very low |  |
| ARB vs Placebo | 12 | No concerns | Low risk | No concerns | No concerns | Major concerns | Major concerns | Very low |  |
| ARB vs nonRASI | 2 | Some concerns | Low risk | No concerns | Major concerns | No concerns | No concerns | Low |  |
| ARBandACEI vs ARBandACEIandBB | 1 | Some concerns | Low risk | No concerns | Major concerns | No concerns | No concerns | Low |  |
| ARBandACEI vs ARBandBB | 1 | Some concerns | Low risk | No concerns | Major concerns | No concerns | No concerns | Low |  |
| ARBandACEI vs BB | 1 | No concerns | Low risk | No concerns | Major concerns | No concerns | No concerns | Low |  |
| ARBandACEIandBB vs ARBandBB | 1 | Some concerns | Low risk | No concerns | Major concerns | No concerns | No concerns | Low |  |
| ARBandACEIandBB vs BB | 1 | Some concerns | Low risk | No concerns | Major concerns | No concerns | No concerns | Low |  |
| ARBandBB vs BB | 1 | Some concerns | Low risk | No concerns | Major concerns | No concerns | No concerns | Low |  |
| ARBandCCB vs ARBandDI | 1 | No concerns | Low risk | No concerns | Major concerns | No concerns | No concerns | Low |  |
| ARBandCCB vs CCBandBB | 1 | Some concerns | Low risk | No concerns | Major concerns | No concerns | No concerns | Low |  |
| ARBandCCB vs CCBandDI | 1 | Some concerns | Low risk | No concerns | Major concerns | No concerns | No concerns | Low |  |
| ARBandDI vs Placebo | 1 | No concerns | Low risk | No concerns | Major concerns | No concerns | No concerns | Low |  |
| BB vs CCB | 3 | Some concerns | Low risk | No concerns | No concerns | Major concerns | No concerns | Low |  |
| BB vs DI | 2 | Some concerns | Low risk | No concerns | No concerns | Major concerns | No concerns | Low |  |
| BB vs Placebo | 5 | Some concerns | Low risk | No concerns | Major concerns | No concerns | No concerns | Low |  |
| BB vs nonBB | 1 | Some concerns | Low risk | No concerns | Major concerns | No concerns | No concerns | Low |  |
| CCB vs Conventionaltherapy | 3 | Some concerns | Low risk | No concerns | Major concerns | No concerns | No concerns | Low |  |
| CCB vs DI | 5 | No concerns | Low risk | No concerns | Major concerns | No concerns | No concerns | Low |  |
| CCB vs Placebo | 7 | No concerns | Low risk | No concerns | No concerns | No concerns | No concerns | High |  |
| CCBandBB vs CCBandDI | 1 | Some concerns | Low risk | No concerns | No concerns | No concerns | No concerns | Moderate |  |
| Conventionaltherapy vs Placebo | 5 | No concerns | Low risk | No concerns | No concerns | No concerns | No concerns | High |  |
| DI vs Placebo | 6 | No concerns | Low risk | No concerns | No concerns | No concerns | No concerns | High |  |
| Placebo vs RI | 1 | No concerns | Low risk | No concerns | Major concerns | No concerns | No concerns | Low |  |
| Indirect evidence | | | | | | | |  |  |
| ACEI vs ACEIandCCB | -- | No concerns | Low risk | No concerns | No concerns | Major concerns | No concerns | Low |  |
| ACEI vs ACEIandDI | -- | No concerns | Low risk | No concerns | Major concerns | No concerns | No concerns | Low |  |
| ACEI vs ARBandCCB | -- | No concerns | Low risk | No concerns | Major concerns | No concerns | No concerns | Low |  |
| ACEI vs ARBandDI | -- | No concerns | Low risk | No concerns | Major concerns | No concerns | No concerns | Low |  |
| ACEI vs BBandDI | -- | No concerns | Low risk | No concerns | Major concerns | No concerns | No concerns | Low |  |
| ACEI vs CCBandBB | -- | No concerns | Low risk | No concerns | Major concerns | No concerns | No concerns | Low |  |
| ACEI vs CCBandDI | -- | No concerns | Low risk | No concerns | Major concerns | No concerns | No concerns | Low |  |
| ACEI vs RI | -- | No concerns | Low risk | No concerns | No concerns | Major concerns | No concerns | Low |  |
| ACEI vs nonBB | -- | Some concerns | Low risk | No concerns | Major concerns | No concerns | No concerns | Low |  |
| ACEIandBB vs ACEIandCCB | -- | No concerns | Low risk | No concerns | Major concerns | No concerns | No concerns | Low |  |
| ACEIandBB vs ACEIandDI | -- | Some concerns | Low risk | No concerns | Major concerns | No concerns | No concerns | Low |  |
| ACEIandBB vs ARB | -- | Some concerns | Low risk | No concerns | Major concerns | No concerns | No concerns | Low |  |
| ACEIandBB vs ARBandCCB | -- | No concerns | Low risk | No concerns | Major concerns | No concerns | No concerns | Low |  |
| ACEIandBB vs ARBandDI | -- | No concerns | Low risk | No concerns | Major concerns | No concerns | No concerns | Low |  |
| ACEIandBB vs BBandDI | -- | Some concerns | Low risk | No concerns | Major concerns | No concerns | No concerns | Low |  |
| ACEIandBB vs CCB | -- | Some concerns | Low risk | No concerns | Major concerns | No concerns | No concerns | Low |  |
| ACEIandBB vs CCBandBB | -- | No concerns | Low risk | No concerns | Major concerns | No concerns | No concerns | Low |  |
| ACEIandBB vs CCBandDI | -- | No concerns | Low risk | No concerns | Major concerns | No concerns | No concerns | Low |  |
| ACEIandBB vs Conventionaltherapy | -- | Some concerns | Low risk | No concerns | Major concerns | No concerns | No concerns | Low |  |
| ACEIandBB vs DI | -- | Some concerns | Low risk | No concerns | Major concerns | No concerns | No concerns | Low |  |
| ACEIandBB vs Placebo | -- | Some concerns | Low risk | No concerns | No concerns | No concerns | No concerns | Moderate |  |
| ACEIandBB vs RI | -- | No concerns | Low risk | No concerns | No concerns | No concerns | No concerns | High |  |
| ACEIandBB vs nonBB | -- | Some concerns | Low risk | No concerns | Major concerns | No concerns | No concerns | Low |  |
| ACEIandBB vs nonRASI | -- | Some concerns | Low risk | No concerns | No concerns | No concerns | No concerns | Moderate |  |
| ACEIandCCB vs ARB | -- | No concerns | Low risk | No concerns | No concerns | Major concerns | No concerns | Low |  |
| ACEIandCCB vs ARBandACEI | -- | No concerns | Low risk | No concerns | Major concerns | No concerns | No concerns | Low |  |
| ACEIandCCB vs ARBandACEIandBB | -- | No concerns | Low risk | No concerns | Major concerns | No concerns | No concerns | Low |  |
| ACEIandCCB vs ARBandBB | -- | No concerns | Low risk | No concerns | Major concerns | No concerns | No concerns | Low |  |
| ACEIandCCB vs ARBandCCB | -- | No concerns | Low risk | No concerns | Major concerns | No concerns | No concerns | Low |  |
| ACEIandCCB vs ARBandDI | -- | No concerns | Low risk | No concerns | Major concerns | No concerns | No concerns | Low |  |
| ACEIandCCB vs BB | -- | No concerns | Low risk | No concerns | No concerns | No concerns | No concerns | High |  |
| ACEIandCCB vs CCBandBB | -- | No concerns | Low risk | No concerns | Major concerns | No concerns | No concerns | Low |  |
| ACEIandCCB vs CCBandDI | -- | No concerns | Low risk | No concerns | Major concerns | No concerns | No concerns | Low |  |
| ACEIandCCB vs Conventionaltherapy | -- | No concerns | Low risk | No concerns | Major concerns | No concerns | No concerns | Low |  |
| ACEIandCCB vs DI | -- | No concerns | Low risk | No concerns | Major concerns | No concerns | No concerns | Low |  |
| ACEIandCCB vs RI | -- | No concerns | Low risk | No concerns | No concerns | No concerns | No concerns | High |  |
| ACEIandCCB vs nonBB | -- | Some concerns | Low risk | No concerns | Major concerns | No concerns | No concerns | Low |  |
| ACEIandCCB vs nonRASI | -- | No concerns | Low risk | No concerns | No concerns | No concerns | No concerns | High |  |
| ACEIandDI vs ARB | -- | No concerns | Low risk | No concerns | Major concerns | No concerns | No concerns | Low |  |
| ACEIandDI vs ARBandACEI | -- | No concerns | Low risk | No concerns | Major concerns | No concerns | No concerns | Low |  |
| ACEIandDI vs ARBandACEIandBB | -- | Some concerns | Low risk | No concerns | Major concerns | No concerns | No concerns | Low |  |
| ACEIandDI vs ARBandBB | -- | Some concerns | Low risk | No concerns | Major concerns | No concerns | No concerns | Low |  |
| ACEIandDI vs ARBandCCB | -- | No concerns | Low risk | No concerns | Major concerns | No concerns | No concerns | Low |  |
| ACEIandDI vs ARBandDI | -- | No concerns | Low risk | No concerns | Major concerns | No concerns | No concerns | Low |  |
| ACEIandDI vs BB | -- | No concerns | Low risk | No concerns | Major concerns | No concerns | No concerns | Low |  |
| ACEIandDI vs BBandDI | -- | No concerns | Low risk | No concerns | Major concerns | No concerns | No concerns | Low |  |
| ACEIandDI vs CCB | -- | No concerns | Low risk | No concerns | Major concerns | No concerns | No concerns | Low |  |
| ACEIandDI vs CCBandBB | -- | No concerns | Low risk | No concerns | Major concerns | No concerns | No concerns | Low |  |
| ACEIandDI vs CCBandDI | -- | No concerns | Low risk | No concerns | Major concerns | No concerns | No concerns | Low |  |
| ACEIandDI vs Conventionaltherapy | -- | No concerns | Low risk | No concerns | Major concerns | No concerns | No concerns | Low |  |
| ACEIandDI vs DI | -- | No concerns | Low risk | No concerns | Major concerns | No concerns | No concerns | Low |  |
| ACEIandDI vs RI | -- | No concerns | Low risk | No concerns | No concerns | No concerns | No concerns | High |  |
| ACEIandDI vs nonBB | -- | Some concerns | Low risk | No concerns | Major concerns | No concerns | No concerns | Low |  |
| ACEIandDI vs nonRASI | -- | Some concerns | Low risk | No concerns | No concerns | Major concerns | No concerns | Low |  |
| ARB vs ARBandACEIandBB | -- | Some concerns | Low risk | No concerns | Major concerns | No concerns | No concerns | Low |  |
| ARB vs ARBandBB | -- | Some concerns | Low risk | No concerns | Major concerns | No concerns | No concerns | Low |  |
| ARB vs ARBandCCB | -- | No concerns | Low risk | No concerns | Major concerns | No concerns | No concerns | Low |  |
| ARB vs ARBandDI | -- | No concerns | Low risk | No concerns | Major concerns | No concerns | No concerns | Low |  |
| ARB vs BBandDI | -- | No concerns | Low risk | No concerns | Major concerns | No concerns | No concerns | Low |  |
| ARB vs CCBandBB | -- | No concerns | Low risk | No concerns | Major concerns | No concerns | No concerns | Low |  |
| ARB vs CCBandDI | -- | No concerns | Low risk | No concerns | Major concerns | No concerns | No concerns | Low |  |
| ARB vs Conventionaltherapy | -- | No concerns | Low risk | No concerns | Major concerns | No concerns | No concerns | Low |  |
| ARB vs DI | -- | No concerns | Low risk | No concerns | No concerns | Major concerns | No concerns | Low |  |
| ARB vs RI | -- | No concerns | Low risk | No concerns | No concerns | Major concerns | No concerns | Low |  |
| ARB vs nonBB | -- | Some concerns | Low risk | No concerns | Major concerns | No concerns | No concerns | Low |  |
| ARBandACEI vs ARBandCCB | -- | No concerns | Low risk | No concerns | Major concerns | No concerns | No concerns | Low |  |
| ARBandACEI vs ARBandDI | -- | No concerns | Low risk | No concerns | Major concerns | No concerns | No concerns | Low |  |
| ARBandACEI vs BBandDI | -- | No concerns | Low risk | No concerns | Major concerns | No concerns | No concerns | Low |  |
| ARBandACEI vs CCB | -- | No concerns | Low risk | No concerns | Major concerns | No concerns | No concerns | Low |  |
| ARBandACEI vs CCBandBB | -- | No concerns | Low risk | No concerns | Major concerns | No concerns | No concerns | Low |  |
| ARBandACEI vs CCBandDI | -- | No concerns | Low risk | No concerns | Major concerns | No concerns | No concerns | Low |  |
| ARBandACEI vs Conventionaltherapy | -- | No concerns | Low risk | No concerns | Major concerns | No concerns | No concerns | Low |  |
| ARBandACEI vs DI | -- | No concerns | Low risk | No concerns | Major concerns | No concerns | No concerns | Low |  |
| ARBandACEI vs Placebo | -- | No concerns | Low risk | No concerns | No concerns | Major concerns | No concerns | Low |  |
| ARBandACEI vs RI | -- | No concerns | Low risk | No concerns | No concerns | Major concerns | No concerns | Low |  |
| ARBandACEI vs nonBB | -- | Some concerns | Low risk | No concerns | Major concerns | No concerns | No concerns | Low |  |
| ARBandACEI vs nonRASI | -- | No concerns | Low risk | No concerns | Major concerns | No concerns | No concerns | Low |  |
| ARBandACEIandBB vs ARBandCCB | -- | No concerns | Low risk | No concerns | Major concerns | No concerns | No concerns | Low |  |
| ARBandACEIandBB vs ARBandDI | -- | No concerns | Low risk | No concerns | Major concerns | No concerns | No concerns | Low |  |
| ARBandACEIandBB vs BBandDI | -- | Some concerns | Low risk | No concerns | Major concerns | No concerns | No concerns | Low |  |
| ARBandACEIandBB vs CCB | -- | Some concerns | Low risk | No concerns | Major concerns | No concerns | No concerns | Low |  |
| ARBandACEIandBB vs CCBandBB | -- | No concerns | Low risk | No concerns | Major concerns | No concerns | No concerns | Low |  |
| ARBandACEIandBB vs CCBandDI | -- | No concerns | Low risk | No concerns | Major concerns | No concerns | No concerns | Low |  |
| ARBandACEIandBB vs Conventionaltherapy | -- | Some concerns | Low risk | No concerns | Major concerns | No concerns | No concerns | Low |  |
| ARBandACEIandBB vs DI | -- | Some concerns | Low risk | No concerns | Major concerns | No concerns | No concerns | Low |  |
| ARBandACEIandBB vs Placebo | -- | Some concerns | Low risk | No concerns | Major concerns | No concerns | No concerns | Low |  |
| ARBandACEIandBB vs RI | -- | No concerns | Low risk | No concerns | No concerns | No concerns | No concerns | High |  |
| ARBandACEIandBB vs nonBB | -- | Some concerns | Low risk | No concerns | Major concerns | No concerns | No concerns | Low |  |
| ARBandACEIandBB vs nonRASI | -- | Some concerns | Low risk | No concerns | Major concerns | No concerns | No concerns | Low |  |
| ARBandBB vs ARBandCCB | -- | No concerns | Low risk | No concerns | Major concerns | No concerns | No concerns | Low |  |
| ARBandBB vs ARBandDI | -- | No concerns | Low risk | No concerns | Major concerns | No concerns | No concerns | Low |  |
| ARBandBB vs BBandDI | -- | Some concerns | Low risk | No concerns | Major concerns | No concerns | No concerns | Low |  |
| ARBandBB vs CCB | -- | Some concerns | Low risk | No concerns | Major concerns | No concerns | No concerns | Low |  |
| ARBandBB vs CCBandBB | -- | No concerns | Low risk | No concerns | Major concerns | No concerns | No concerns | Low |  |
| ARBandBB vs CCBandDI | -- | No concerns | Low risk | No concerns | Major concerns | No concerns | No concerns | Low |  |
| ARBandBB vs Conventionaltherapy | -- | Some concerns | Low risk | No concerns | Major concerns | No concerns | No concerns | Low |  |
| ARBandBB vs DI | -- | Some concerns | Low risk | No concerns | Major concerns | No concerns | No concerns | Low |  |
| ARBandBB vs Placebo | -- | Some concerns | Low risk | No concerns | Major concerns | No concerns | No concerns | Low |  |
| ARBandBB vs RI | -- | No concerns | Low risk | No concerns | Major concerns | No concerns | No concerns | Low |  |
| ARBandBB vs nonBB | -- | Some concerns | Low risk | No concerns | Major concerns | No concerns | No concerns | Low |  |
| ARBandBB vs nonRASI | -- | Some concerns | Low risk | No concerns | Major concerns | No concerns | No concerns | Low |  |
| ARBandCCB vs BB | -- | No concerns | Low risk | No concerns | Major concerns | No concerns | No concerns | Low |  |
| ARBandCCB vs BBandDI | -- | No concerns | Low risk | No concerns | Major concerns | No concerns | No concerns | Low |  |
| ARBandCCB vs CCB | -- | No concerns | Low risk | No concerns | Major concerns | No concerns | No concerns | Low |  |
| ARBandCCB vs Conventionaltherapy | -- | No concerns | Low risk | No concerns | Major concerns | No concerns | No concerns | Low |  |
| ARBandCCB vs DI | -- | No concerns | Low risk | No concerns | Major concerns | No concerns | No concerns | Low |  |
| ARBandCCB vs Placebo | -- | No concerns | Low risk | No concerns | Major concerns | No concerns | No concerns | Low |  |
| ARBandCCB vs RI | -- | No concerns | Low risk | No concerns | Major concerns | No concerns | No concerns | Low |  |
| ARBandCCB vs nonBB | -- | No concerns | Low risk | No concerns | Major concerns | No concerns | No concerns | Low |  |
| ARBandCCB vs nonRASI | -- | No concerns | Low risk | No concerns | Major concerns | No concerns | No concerns | Low |  |
| ARBandDI vs BB | -- | No concerns | Low risk | No concerns | Major concerns | No concerns | No concerns | Low |  |
| ARBandDI vs BBandDI | -- | No concerns | Low risk | No concerns | Major concerns | No concerns | No concerns | Low |  |
| ARBandDI vs CCB | -- | No concerns | Low risk | No concerns | Major concerns | No concerns | No concerns | Low |  |
| ARBandDI vs CCBandBB | -- | Some concerns | Low risk | No concerns | Major concerns | No concerns | No concerns | Low |  |
| ARBandDI vs CCBandDI | -- | Some concerns | Low risk | No concerns | Major concerns | No concerns | No concerns | Low |  |
| ARBandDI vs Conventionaltherapy | -- | No concerns | Low risk | No concerns | Major concerns | No concerns | No concerns | Low |  |
| ARBandDI vs DI | -- | No concerns | Low risk | No concerns | Major concerns | No concerns | No concerns | Low |  |
| ARBandDI vs RI | -- | No concerns | Low risk | No concerns | Major concerns | No concerns | No concerns | Low |  |
| ARBandDI vs nonBB | -- | No concerns | Low risk | No concerns | Major concerns | No concerns | No concerns | Low |  |
| ARBandDI vs nonRASI | -- | No concerns | Low risk | No concerns | Major concerns | No concerns | No concerns | Low |  |
| BB vs BBandDI | -- | Some concerns | Low risk | No concerns | Major concerns | No concerns | No concerns | Low |  |
| BB vs CCBandBB | -- | No concerns | Low risk | No concerns | Major concerns | No concerns | No concerns | Low |  |
| BB vs CCBandDI | -- | No concerns | Low risk | No concerns | Major concerns | No concerns | No concerns | Low |  |
| BB vs Conventionaltherapy | -- | Some concerns | Low risk | No concerns | Major concerns | No concerns | No concerns | Low |  |
| BB vs RI | -- | No concerns | Low risk | No concerns | Major concerns | No concerns | No concerns | Low |  |
| BB vs nonRASI | -- | Some concerns | Low risk | No concerns | Major concerns | No concerns | No concerns | Low |  |
| BBandDI vs CCB | -- | No concerns | Low risk | No concerns | Major concerns | No concerns | No concerns | Low |  |
| BBandDI vs CCBandBB | -- | No concerns | Low risk | No concerns | Major concerns | No concerns | No concerns | Low |  |
| BBandDI vs CCBandDI | -- | No concerns | Low risk | No concerns | Major concerns | No concerns | No concerns | Low |  |
| BBandDI vs Conventionaltherapy | -- | No concerns | Low risk | No concerns | Major concerns | No concerns | No concerns | Low |  |
| BBandDI vs DI | -- | No concerns | Low risk | No concerns | Major concerns | No concerns | No concerns | Low |  |
| BBandDI vs Placebo | -- | No concerns | Low risk | No concerns | Major concerns | No concerns | No concerns | Low |  |
| BBandDI vs RI | -- | No concerns | Low risk | No concerns | Major concerns | No concerns | No concerns | Low |  |
| BBandDI vs nonBB | -- | Some concerns | Low risk | No concerns | Major concerns | No concerns | No concerns | Low |  |
| BBandDI vs nonRASI | -- | Some concerns | Low risk | No concerns | Major concerns | No concerns | No concerns | Low |  |
| CCB vs CCBandBB | -- | No concerns | Low risk | No concerns | Major concerns | No concerns | No concerns | Low |  |
| CCB vs CCBandDI | -- | No concerns | Low risk | No concerns | Major concerns | No concerns | No concerns | Low |  |
| CCB vs RI | -- | No concerns | Low risk | No concerns | No concerns | No concerns | No concerns | High |  |
| CCB vs nonBB | -- | Some concerns | Low risk | No concerns | Major concerns | No concerns | No concerns | Low |  |
| CCB vs nonRASI | -- | Some concerns | Low risk | No concerns | No concerns | Major concerns | No concerns | Low |  |
| CCBandBB vs Conventionaltherapy | -- | No concerns | Low risk | No concerns | Major concerns | No concerns | No concerns | Low |  |
| CCBandBB vs DI | -- | No concerns | Low risk | No concerns | Major concerns | No concerns | No concerns | Low |  |
| CCBandBB vs Placebo | -- | No concerns | Low risk | No concerns | Major concerns | No concerns | No concerns | Low |  |
| CCBandBB vs RI | -- | No concerns | Low risk | No concerns | Major concerns | No concerns | No concerns | Low |  |
| CCBandBB vs nonBB | -- | Some concerns | Low risk | No concerns | Major concerns | No concerns | No concerns | Low |  |
| CCBandBB vs nonRASI | -- | No concerns | Low risk | No concerns | Major concerns | No concerns | No concerns | Low |  |
| CCBandDI vs Conventionaltherapy | -- | No concerns | Low risk | No concerns | Major concerns | No concerns | No concerns | Low |  |
| CCBandDI vs DI | -- | No concerns | Low risk | No concerns | Major concerns | No concerns | No concerns | Low |  |
| CCBandDI vs Placebo | -- | No concerns | Low risk | No concerns | Major concerns | No concerns | No concerns | Low |  |
| CCBandDI vs RI | -- | No concerns | Low risk | No concerns | Major concerns | No concerns | No concerns | Low |  |
| CCBandDI vs nonBB | -- | Some concerns | Low risk | No concerns | Major concerns | No concerns | No concerns | Low |  |
| CCBandDI vs nonRASI | -- | No concerns | Low risk | No concerns | Major concerns | No concerns | No concerns | Low |  |
| Conventionaltherapy vs DI | -- | No concerns | Low risk | No concerns | Major concerns | No concerns | No concerns | Low |  |
| Conventionaltherapy vs RI | -- | No concerns | Low risk | No concerns | No concerns | No concerns | No concerns | High |  |
| Conventionaltherapy vs nonBB | -- | Some concerns | Low risk | No concerns | Major concerns | No concerns | No concerns | Low |  |
| Conventionaltherapy vs nonRASI | -- | Some concerns | Low risk | No concerns | No concerns | Major concerns | No concerns | Low |  |
| DI vs RI | -- | No concerns | Low risk | No concerns | No concerns | No concerns | No concerns | High |  |
| DI vs nonBB | -- | Some concerns | Low risk | No concerns | Major concerns | No concerns | No concerns | Low |  |
| DI vs nonRASI | -- | Some concerns | Low risk | No concerns | No concerns | No concerns | No concerns | Moderate |  |
| Placebo vs nonBB | -- | Some concerns | Low risk | No concerns | Major concerns | No concerns | No concerns | Low |  |
| Placebo vs nonRASI | -- | Some concerns | Low risk | No concerns | Major concerns | No concerns | No concerns | Low |  |
| RI vs nonBB | -- | No concerns | Low risk | No concerns | Major concerns | No concerns | No concerns | Low |  |
| RI vs nonRASI | -- | No concerns | Low risk | No concerns | Major concerns | No concerns | No concerns | Low |  |
| nonBB vs nonRASI | -- | Some concerns | Low risk | No concerns | Major concerns | No concerns | No concerns | Low |  |

Abbreviations: ARB, angiotensin receptor blockers; DI, Diuretics; CCB, calcium channel blockers; ACEI, angiotensin-converting enzyme inhibitor; BB, βadrenergic receptor blockers; nonRASI, non-renin-angiotensin system (RAS) inhibitors; RI, renin inhibitors.
