# Supplementary material for: Comparative efficacy of different antihypertensive drug classes for stroke prevention: A network meta-analysis of randomized controlled trials
Source: PLoS One. 2025 Feb 21;20(2):e0313309. doi: 10.1371/journal.pone.0313309 (PMC11845040; doi:10.1371/journal.pone.0313309)
Supplement: S18 Table — (DOCX) [file pone.0313309.s019.docx]

**S18 Table. Reasons for downgrading the confidence in the results.**

| **Outcome domains** | **Direct comparisons**  **N** | **Within-study bias**  **N( %)** | **Reporting bias**  **N( %)** | **Intransitivity**  **N( %)** | **Imprecision**  **N( %)** | **Heterogeneity**  **N( %)** | **Incoherence**  **N( %)** |
| --- | --- | --- | --- | --- | --- | --- | --- |
| **For the overall population** |  |  |  |  |  |  |  |
| Stroke | 46 | 23(50%) | 0(0) | 0(0) | 34(74%) | 7(15%) | 3(7%) |
| All-cause mortality | 44 | 22(50%) | 0(0) | 0(0) | 32(73%) | 5(11%) | 1(2%) |
| Cardiovascular mortality | 43 | 20(47%) | 0(0) | 0(0) | 33(77%) | 2(5%) | 1(2%) |
| **For hypertensive patients** |  |  |  |  |  |  |  |
| Stroke | 41 | 27(66%) | 0(0) | 0(0) | 31(76%) | 4(10%) | 2(5%) |
| All-cause mortality | 42 | 29(69%) | 0(0) | 0(0) | 30(71%) | 5(12%) | 1(2%) |
| Cardiovascular mortality | 41 | 26(63%) | 0(0) | 0(0) | 30(73%) | 3(7%) | 1(2%) |
