# Supplementary material for: Comparative efficacy of different antihypertensive drug classes for stroke prevention: A network meta-analysis of randomized controlled trials
Source: PLoS One. 2025 Feb 21;20(2):e0313309. doi: 10.1371/journal.pone.0313309 (PMC11845040; doi:10.1371/journal.pone.0313309)
Supplement: S19 Table — (DOCX) [file pone.0313309.s020.docx]

**S19 Table. Relative risk [RR] with 95% CrI for stroke of the overall population.**

| **ACEI** | 0.56 (0.26, 1.12) | **0.70 (0.51, 0.96)** | 0.89 (0.72, 1.10) | 0.97 (0.87, 1.09) | 0.96 (0.80, 1.17) | 0.63 (0.31, 1.24) | 0.94 (0.37, 2.06) | 0.94 (0.52, 1.68) | 0.96 (0.64, 1.45) | 1.07 (0.91, 1.26) | 0.90 (0.58, 1.38) | **0.87 (0.78, 0.98)** | 1.44 (0.60, 3.50) | 0.63 (0.23, 1.64) | 0.89 (0.77, 1.04) | **0.82 (0.70, 0.94)** | 1.08 (0.65, 1.78) | 1.28 (0.92, 1.79) | **1.19 (1.08, 1.31)** | **1.43 (0.99, 2.06)** |
| --- | --- | --- | --- | --- | --- | --- | --- | --- | --- | --- | --- | --- | --- | --- | --- | --- | --- | --- | --- | --- |
| 1.78 (0.90, 3.82) | **ACEI+**  **BB** | 1.24 (0.58, 2.81) | 1.58 (0.78, 3.47) | 1.73 (0.87, 3.73) | 1.72 (0.87, 3.70) | 1.12 (0.50, 2.60) | 1.67 (0.61, 4.24) | 1.67 (0.68, 4.33) | 1.71 (0.78, 4.03) | 1.91 (0.95, 4.12) | 1.61 (0.71, 3.81) | 1.56 (0.78, 3.35) | 2.59 (0.83, 8.14) | 1.13 (0.34, 3.81) | 1.59 (0.79, 3.44) | 1.45 (0.72, 3.15) | 1.93 (0.82, 4.74) | **2.29 (1.07, 5.20)** | **2.12 (1.07, 4.55)** | **2.55 (1.17, 5.85)** |
| **1.43 (1.04, 1.98)** | 0.80 (0.36, 1.72) | **ACEI+**  **CCB** | 1.27 (0.95, 1.71) | **1.39 (1.02, 1.93)** | 1.38 (0.97, 2.00) | 0.90 (0.42, 1.89) | 1.34 (0.50, 3.12) | 1.34 (0.70, 2.59) | 1.38 (0.83, 2.28) | **1.54 (1.10, 2.15)** | 1.29 (0.96, 1.74) | 1.25 (0.92, 1.72) | 2.06 (0.82, 5.25) | 0.90 (0.32, 2.46) | 1.28 (0.92, 1.79) | 1.17 (0.84, 1.63) | 1.54 (0.86, 2.77) | **1.83 (1.17, 2.86)** | **1.70 (1.26, 2.33)** | **2.04 (1.29, 3.27)** |
| 1.12 (0.91, 1.39) | 0.63 (0.29, 1.28) | 0.79 (0.58, 1.05) | **ACEI+DI** | 1.09 (0.89, 1.35) | 1.08 (0.83, 1.43) | 0.71 (0.34, 1.42) | 1.05 (0.41, 2.36) | 1.05 (0.57, 1.94) | 1.08 (0.69, 1.68) | 1.21 (0.94, 1.53) | 1.01 (0.67, 1.53) | 0.98 (0.79, 1.21) | 1.62 (0.67, 3.97) | 0.71 (0.26, 1.85) | 1.01 (0.79, 1.27) | 0.92 (0.72, 1.16) | 1.21 (0.71, 2.06) | 1.44 (0.99, 2.09) | **1.34 (1.11, 1.62)** | **1.61 (1.08, 2.40)** |
| 1.03 (0.92, 1.14) | 0.58 (0.27, 1.15) | **0.72 (0.52, 0.98)** | 0.91 (0.74, 1.13) | **ARB** | 0.99 (0.82, 1.19) | 0.64 (0.31, 1.27) | 0.96 (0.38, 2.11) | 0.96 (0.54, 1.72) | 0.99 (0.66, 1.49) | 1.10 (0.94, 1.29) | 0.92 (0.60, 1.42) | **0.90 (0.80, 1.00)** | 1.48 (0.62, 3.59) | 0.65 (0.24, 1.68) | 0.92 (0.78, 1.08) | **0.84 (0.71, 0.98)** | 1.11 (0.67, 1.83) | 1.31 (0.96, 1.80) | **1.22 (1.11, 1.35)** | **1.47 (1.02, 2.11)** |
| 1.04 (0.86, 1.25) | 0.58 (0.27, 1.16) | 0.72 (0.50, 1.04) | 0.92 (0.70, 1.20) | 1.01 (0.84, 1.21) | **ARB+**  **ACEI** | 0.65 (0.32, 1.29) | 0.97 (0.38, 2.14) | 0.97 (0.52, 1.78) | 1.00 (0.64, 1.55) | 1.11 (0.88, 1.39) | 0.94 (0.58, 1.48) | 0.91 (0.74, 1.11) | 1.50 (0.61, 3.67) | 0.65 (0.24, 1.72) | 0.93 (0.73, 1.16) | 0.85 (0.67, 1.06) | 1.12 (0.65, 1.89) | 1.33 (0.92, 1.90) | **1.24 (1.02, 1.50)** | 1.48 (0.99, 2.21) |
| 1.59 (0.81, 3.24) | 0.89 (0.38, 2.00) | 1.11 (0.53, 2.41) | 1.42 (0.70, 2.96) | 1.55 (0.78, 3.18) | 1.54 (0.77, 3.14) | **ARB+**  **ACEI+BB** | 1.48 (0.54, 3.70) | 1.49 (0.61, 3.72) | 1.53 (0.70, 3.45) | 1.71 (0.86, 3.50) | 1.44 (0.65, 3.26) | 1.39 (0.70, 2.86) | 2.31 (0.75, 7.05) | 1.01 (0.30, 3.26) | 1.42 (0.71, 2.94) | 1.30 (0.65, 2.68) | 1.72 (0.74, 4.08) | 2.04 (0.97, 4.46) | 1.90 (0.96, 3.89) | **2.28 (1.05, 5.03)** |
| 1.07 (0.49, 2.73) | 0.60 (0.24, 1.65) | 0.75 (0.32, 1.99) | 0.95 (0.42, 2.46) | 1.04 (0.47, 2.66) | 1.03 (0.47, 2.63) | 0.67 (0.27, 1.85) | **ARB+BB** | 1.01 (0.37, 2.95) | 1.04 (0.42, 2.83) | 1.15 (0.52, 2.93) | 0.97 (0.39, 2.67) | 0.93 (0.42, 2.39) | 1.55 (0.47, 5.45) | 0.68 (0.19, 2.54) | 0.96 (0.43, 2.46) | 0.87 (0.39, 2.24) | 1.16 (0.46, 3.29) | 1.37 (0.59, 3.62) | 1.28 (0.58, 3.25) | 1.53 (0.64, 4.17) |
| 1.07 (0.60, 1.93) | 0.60 (0.23, 1.48) | 0.75 (0.39, 1.43) | 0.95 (0.52, 1.75) | 1.04 (0.58, 1.86) | 1.03 (0.56, 1.91) | 0.67 (0.27, 1.64) | 0.99 (0.34, 2.69) | **ARB+**  **CCB** | 1.03 (0.67, 1.57) | 1.15 (0.63, 2.08) | 0.96 (0.47, 1.98) | 0.93 (0.52, 1.68) | 1.54 (0.81, 2.94) | 0.67 (0.30, 1.43) | 0.96 (0.53, 1.74) | 0.87 (0.48, 1.58) | 1.15 (0.53, 2.48) | 1.37 (0.70, 2.65) | 1.27 (0.72, 2.27) | 1.53 (0.77, 3.01) |
| 1.04 (0.69, 1.57) | 0.58 (0.25, 1.28) | 0.73 (0.44, 1.20) | 0.92 (0.59, 1.44) | 1.01 (0.67, 1.52) | 1.00 (0.65, 1.57) | 0.65 (0.29, 1.43) | 0.96 (0.35, 2.36) | 0.97 (0.64, 1.49) | **ARB+DI** | 1.11 (0.73, 1.71) | 0.94 (0.52, 1.67) | 0.91 (0.60, 1.37) | 1.50 (0.69, 3.25) | 0.65 (0.27, 1.55) | 0.93 (0.61, 1.42) | 0.85 (0.55, 1.29) | 1.12 (0.59, 2.11) | 1.33 (0.79, 2.23) | 1.24 (0.83, 1.85) | 1.48 (0.87, 2.54) |
| 0.93 (0.79, 1.10) | 0.52 (0.24, 1.05) | **0.65 (0.46, 0.91)** | 0.83 (0.65, 1.06) | 0.91 (0.78, 1.07) | 0.90 (0.72, 1.14) | 0.59 (0.29, 1.17) | 0.87 (0.34, 1.93) | 0.87 (0.48, 1.59) | 0.90 (0.59, 1.37) | **BB** | 0.84 (0.53, 1.32) | **0.81 (0.70, 0.96)** | 1.34 (0.56, 3.28) | 0.59 (0.22, 1.54) | 0.83 (0.68, 1.01) | **0.76 (0.63, 0.92)** | 1.01 (0.62, 1.62) | 1.19 (0.84, 1.70) | 1.11 (0.96, 1.30) | 1.33 (0.91, 1.96) |
| 1.11 (0.72, 1.72) | 0.62 (0.26, 1.41) | 0.78 (0.58, 1.05) | 0.99 (0.65, 1.50) | 1.08 (0.70, 1.68) | 1.07 (0.68, 1.73) | 0.70 (0.31, 1.55) | 1.04 (0.37, 2.54) | 1.04 (0.51, 2.15) | 1.07 (0.60, 1.93) | 1.19 (0.76, 1.87) | **BB+**  **DI** | 0.97 (0.63, 1.50) | 1.60 (0.61, 4.28) | 0.70 (0.24, 1.99) | 0.99 (0.63, 1.55) | 0.91 (0.58, 1.42) | 1.20 (0.62, 2.31) | 1.42 (0.83, 2.42) | 1.32 (0.87, 2.04) | 1.59 (0.92, 2.76) |
| **1.14 (1.02, 1.28)** | 0.64 (0.30, 1.28) | 0.80 (0.58, 1.09) | 1.02 (0.82, 1.26) | **1.12 (1.00, 1.25)** | 1.10 (0.90, 1.36) | 0.72 (0.35, 1.42) | 1.07 (0.42, 2.36) | 1.07 (0.59, 1.92) | 1.10 (0.73, 1.66) | **1.23 (1.05, 1.44)** | 1.03 (0.67, 1.58) | **CCB** | 1.65 (0.69, 4.00) | 0.72 (0.27, 1.88) | 1.02 (0.88, 1.18) | 0.94 (0.81, 1.08) | 1.23 (0.74, 2.03) | **1.46 (1.05, 2.05)** | **1.36 (1.23, 1.52)** | **1.64 (1.14, 2.37)** |
| 0.69 (0.29, 1.67) | 0.39 (0.12, 1.20) | 0.49 (0.19, 1.22) | 0.62 (0.25, 1.50) | 0.68 (0.28, 1.61) | 0.67 (0.27, 1.63) | 0.43 (0.14, 1.34) | 0.64 (0.18, 2.12) | 0.65 (0.34, 1.23) | 0.67 (0.31, 1.45) | 0.75 (0.30, 1.80) | 0.63 (0.23, 1.64) | 0.61 (0.25, 1.45) | **CCB+BB** | 0.44 (0.21, 0.89) | 0.62 (0.25, 1.49) | 0.57 (0.23, 1.36) | 0.75 (0.27, 2.04) | 0.89 (0.34, 2.25) | 0.83 (0.34, 1.97) | 0.99 (0.39, 2.54) |
| 1.59 (0.61, 4.27) | 0.89 (0.26, 2.98) | 1.11 (0.41, 3.13) | 1.41 (0.54, 3.85) | 1.55 (0.60, 4.15) | 1.53 (0.58, 4.19) | 0.99 (0.31, 3.34) | 1.47 (0.39, 5.31) | 1.48 (0.70, 3.28) | 1.53 (0.64, 3.77) | 1.70 (0.65, 4.64) | 1.43 (0.50, 4.16) | 1.39 (0.53, 3.74) | **2.29 (1.13, 4.86)** | **CCB+DI** | 1.42 (0.54, 3.83) | 1.30 (0.49, 3.51) | 1.71 (0.58, 5.17) | 2.04 (0.74, 5.72) | 1.89 (0.73, 5.06) | 2.27 (0.82, 6.45) |
| 1.12 (0.97, 1.31) | 0.63 (0.29, 1.26) | 0.78 (0.56, 1.09) | 0.99 (0.79, 1.27) | 1.09 (0.93, 1.29) | 1.08 (0.86, 1.37) | 0.70 (0.34, 1.40) | 1.04 (0.41, 2.33) | 1.05 (0.58, 1.90) | 1.08 (0.70, 1.65) | 1.20 (0.99, 1.46) | 1.01 (0.64, 1.58) | 0.98 (0.85, 1.13) | 1.61 (0.67, 3.95) | 0.70 (0.26, 1.84) | **CT** | 0.91 (0.76, 1.10) | 1.21 (0.72, 2.01) | **1.43 (1.01, 2.04)** | **1.33 (1.15, 1.56)** | **1.60 (1.10, 2.35)** |
| **1.22 (1.06, 1.42)** | 0.69 (0.32, 1.39) | 0.86 (0.61, 1.20) | 1.09 (0.86, 1.38) | **1.19 (1.03, 1.40)** | 1.18 (0.95, 1.49) | 0.77 (0.37, 1.53) | 1.14 (0.45, 2.55) | 1.14 (0.63, 2.09) | 1.18 (0.77, 1.80) | **1.31 (1.09, 1.59)** | 1.10 (0.71, 1.72) | 1.07 (0.93, 1.24) | 1.76 (0.73, 4.30) | 0.77 (0.28, 2.02) | 1.09 (0.91, 1.32) | **DI** | 1.32 (0.79, 2.20) | **1.57 (1.11, 2.23)** | **1.46 (1.27, 1.69)** | **1.75 (1.21, 2.57)** |
| 0.93 (0.56, 1.54) | 0.52 (0.21, 1.21) | 0.65 (0.36, 1.17) | 0.82 (0.49, 1.41) | 0.90 (0.55, 1.50) | 0.89 (0.53, 1.54) | 0.58 (0.24, 1.35) | 0.86 (0.30, 2.19) | 0.87 (0.40, 1.88) | 0.89 (0.47, 1.70) | 0.99 (0.62, 1.61) | 0.84 (0.43, 1.62) | 0.81 (0.49, 1.35) | 1.34 (0.49, 3.69) | 0.58 (0.19, 1.72) | 0.83 (0.5, 1.4) | 0.76 (0.45, 1.27) | **non**  **BB** | 1.19 (0.66, 2.16) | 1.11 (0.67, 1.83) | 1.32 (0.72, 2.46) |
| 0.78 (0.56, 1.09) | **0.44 (0.19, 0.93)** | **0.55 (0.35, 0.85)** | 0.70 (0.48, 1.01) | 0.76 (0.56, 1.04) | 0.75 (0.53, 1.09) | 0.49 (0.22, 1.03) | 0.73 (0.28, 1.70) | 0.73 (0.38, 1.42) | 0.75 (0.45, 1.26) | 0.84 (0.59, 1.19) | 0.70 (0.41, 1.20) | **0.68 (0.49, 0.95)** | 1.12 (0.45, 2.90) | 0.49 (0.17, 1.35) | **0.70 (0.49, 0.99)** | **0.64 (0.45, 0.90)** | 0.84 (0.46, 1.53) | **non**  **RASI** | 0.93 (0.67, 1.29) | 1.12 (0.69, 1.80) |
| **0.84 (0.76, 0.92)** | **0.47 (0.22, 0.94)** | **0.59 (0.43, 0.79)** | **0.75 (0.62, 0.90)** | **0.82 (0.74, 0.90)** | **0.81 (0.67, 0.98)** | 0.53 (0.26, 1.04) | 0.78 (0.31, 1.72) | 0.79 (0.44, 1.40) | 0.81 (0.54, 1.20) | 0.90 (0.77, 1.04) | 0.76 (0.49, 1.15) | **0.73 (0.66, 0.81)** | 1.21 (0.51, 2.92) | 0.53 (0.20, 1.37) | **0.75 (0.64, 0.87)** | **0.69 (0.59, 0.79)** | 0.90 (0.55, 1.49) | 1.07 (0.78, 1.49) | **Placebo** | 1.20 (0.84, 1.70) |
| 0.70 (0.49, 1.01) | **0.39 (0.17, 0.85)** | **0.49 (0.31, 0.78)** | **0.62 (0.42, 0.93)** | **0.68 (0.47, 0.98)** | 0.67 (0.45, 1.01) | **0.44 (0.20, 0.95)** | 0.65 (0.24, 1.55) | 0.65 (0.33, 1.29) | 0.67 (0.39, 1.15) | 0.75 (0.51, 1.10) | 0.63 (0.36, 1.09) | **0.61 (0.42, 0.88)** | 1.01 (0.39, 2.59) | 0.44 (0.15, 1.21) | **0.63 (0.43, 0.91)** | **0.57 (0.39, 0.83)** | 0.76 (0.41, 1.38) | 0.90 (0.56, 1.45) | 0.83 (0.59, 1.19) | **RI** |

Abbreviations: CrI, credible interval; ARB, angiotensin receptor blockers; DI, Diuretics; DI(TL), thiazide-like diuretics; DI(TT), thiazide-type diuretics; CCB, calcium channel blockers; CCB(DH), dihydropyridine calcium channel blockers; CCB(D), calcium channel blockers (diltiazem); CCB(V), calcium channel blockers (verapamil); ACEI, angiotensin-converting enzyme inhibitor; BB, β adrenergic receptor blockers; nonRASI, non-renin-angiotensin system (RAS) inhibitors; RI, renin inhibitors.

Effect sizes represent summary relative risk and 95% credible intervals. Bold values indicate significant results. In the upper triangle, values greater than 1 favor the treatment in the corresponding row, whereas values less than 1 favor the treatment in the corresponding column. In the lower triangle, values greater than 1 favor the treatment in the corresponding column, whereas values less than 1 favor the treatment in the corresponding row.
