# Supplementary material for: Comparative efficacy of different antihypertensive drug classes for stroke prevention: A network meta-analysis of randomized controlled trials
Source: PLoS One. 2025 Feb 21;20(2):e0313309. doi: 10.1371/journal.pone.0313309 (PMC11845040; doi:10.1371/journal.pone.0313309)
Supplement: S21 Table — (DOCX) [file pone.0313309.s022.docx]

**S21 Table. Relative risk [RR] with 95% CrI for subgroup analysis of stroke among hypertensive patients.**

| **ACEI** | 0.70 (0.30, 1.56) | **0.68 (0.48, 0.99)** | 0.83 (0.62, 1.13) | 0.99 (0.82, 1.19) | 1.49 (0.70, 3.24) | 0.79 (0.36, 1.76) | 1.15 (0.43, 2.87) | 1.03 (0.54, 1.99) | 1.06 (0.67, 1.70) | 1.17 (0.93, 1.46) | 0.88 (0.54, 1.46) | 0.90 (0.77, 1.05) | 0.76 (0.50, 1.15) | 1.04 (0.76, 1.41) | 1.59 (0.63, 4.06) | 0.70 (0.25, 1.95) | 0.92 (0.76, 1.11) | 0.93 (0.74, 1.17) | **0.79 (0.63, 0.98)** | 1.17 (0.67, 2.04) | 1.28 (0.88, 1.86) | **1.32 (1.13, 1.56)** |
| --- | --- | --- | --- | --- | --- | --- | --- | --- | --- | --- | --- | --- | --- | --- | --- | --- | --- | --- | --- | --- | --- | --- |
| 1.43 (0.64, 3.28) | **ACEI+**  **BB** | 0.98 (0.41, 2.40) | 1.19 (0.51, 2.88) | 1.41 (0.63, 3.27) | 2.14 (0.96, 4.96) | 1.14 (0.49, 2.64) | 1.66 (0.59, 4.29) | 1.48 (0.54, 4.22) | 1.52 (0.61, 3.93) | 1.67 (0.75, 3.86) | 1.26 (0.50, 3.30) | 1.29 (0.57, 2.98) | 1.09 (0.44, 2.75) | 1.48 (0.64, 3.54) | 2.28 (0.68, 8.01) | 1.00 (0.28, 3.73) | 1.32 (0.58, 3.08) | 1.33 (0.58, 3.12) | 1.13 (0.50, 2.64) | 1.67 (0.64, 4.46) | 1.84 (0.77, 4.49) | 1.89 (0.84, 4.39) |
| **1.46 (1.01, 2.10)** | 1.02 (0.42, 2.42) | **ACEI+**  **CCB** | 1.22 (0.89, 1.69) | **1.44 (1.00, 2.08)** | 2.19 (0.95, 5.05) | 1.16 (0.49, 2.71) | 1.68 (0.59, 4.40) | 1.51 (0.74, 3.08) | 1.55 (0.90, 2.70) | **1.71 (1.15, 2.50)** | 1.29 (0.92, 1.81) | 1.31 (0.92, 1.85) | 1.11 (0.65, 1.87) | 1.52 (0.96, 2.35) | 2.33 (0.88, 6.18) | 1.02 (0.34, 2.94) | 1.35 (0.91, 1.95) | 1.35 (0.91, 2.01) | 1.16 (0.78, 1.70) | 1.71 (0.89, 3.21) | **1.88 (1.14, 3.04)** | **1.93 (1.38, 2.71)** |
| 1.20 (0.88, 1.61) | 0.84 (0.35, 1.95) | 0.82 (0.59, 1.13) | **ACEI+DI** | 1.18 (0.87, 1.59) | 1.80 (0.79, 4.06) | 0.95 (0.41, 2.19) | 1.38 (0.50, 3.53) | 1.24 (0.63, 2.44) | 1.28 (0.77, 2.12) | **1.40 (1.00, 1.93)** | 1.06 (0.66, 1.68) | 1.08 (0.80, 1.42) | 0.91 (0.55, 1.46) | 1.25 (0.83, 1.83) | 1.91 (0.74, 4.96) | 0.84 (0.29, 2.36) | 1.11 (0.80, 1.50) | 1.11 (0.79, 1.55) | 0.95 (0.67, 1.30) | 1.40 (0.75, 2.54) | 1.54 (0.98, 2.37) | **1.58 (1.22, 2.04)** |
| 1.01 (0.84, 1.22) | 0.71 (0.31, 1.59) | **0.69 (0.48, 1.00)** | 0.84 (0.63, 1.14) | **ARB** | 1.52 (0.70, 3.30) | 0.80 (0.36, 1.81) | 1.17 (0.43, 2.92) | 1.05 (0.55, 2.01) | 1.08 (0.68, 1.71) | 1.18 (0.95, 1.46) | 0.89 (0.54, 1.48) | 0.91 (0.78, 1.06) | 0.77 (0.50, 1.18) | 1.05 (0.76, 1.43) | 1.61 (0.64, 4.12) | 0.71 (0.25, 1.97) | 0.93 (0.75, 1.16) | 0.94 (0.73, 1.20) | 0.80 (0.63, 1.01) | 1.18 (0.67, 2.05) | 1.30 (0.93, 1.80) | **1.34 (1.15, 1.57)** |
| 0.67 (0.31, 1.44) | 0.47 (0.20, 1.04) | 0.46 (0.20, 1.05) | 0.56 (0.25, 1.26) | 0.66 (0.30, 1.44) | **ARB+**  **ACEI** | 0.53 (0.24, 1.19) | 0.77 (0.28, 1.93) | 0.69 (0.25, 1.89) | 0.71 (0.29, 1.75) | 0.78 (0.36, 1.69) | 0.59 (0.24, 1.46) | 0.60 (0.27, 1.3) | 0.51 (0.21, 1.21) | 0.69 (0.30, 1.56) | 1.07 (0.32, 3.56) | 0.47 (0.13, 1.64) | 0.61 (0.28, 1.35) | 0.62 (0.28, 1.37) | 0.53 (0.24, 1.17) | 0.78 (0.31, 1.99) | 0.86 (0.37, 1.98) | 0.88 (0.40, 1.93) |
| 1.26 (0.57, 2.80) | 0.88 (0.38, 2.05) | 0.86 (0.37, 2.05) | 1.05 (0.46, 2.45) | 1.25 (0.55, 2.80) | 1.89 (0.84, 4.25) | **ARB+**  **ACEI+BB** | 1.46 (0.53, 3.72) | 1.31 (0.47, 3.65) | 1.35 (0.54, 3.35) | 1.48 (0.66, 3.28) | 1.11 (0.45, 2.82) | 1.13 (0.50, 2.53) | 0.96 (0.39, 2.33) | 1.31 (0.57, 3.03) | 2.00 (0.59, 6.81) | 0.88 (0.24, 3.17) | 1.16 (0.52, 2.61) | 1.17 (0.51, 2.66) | 1.00 (0.44, 2.26) | 1.48 (0.57, 3.82) | 1.62 (0.67, 3.86) | 1.67 (0.75, 3.74) |
| 0.87 (0.35, 2.34) | 0.60 (0.23, 1.69) | 0.59 (0.23, 1.70) | 0.72 (0.28, 2.02) | 0.86 (0.34, 2.32) | 1.30 (0.52, 3.56) | 0.69 (0.27, 1.90) | **ARB+BB** | 0.90 (0.30, 2.95) | 0.93 (0.34, 2.74) | 1.01 (0.41, 2.74) | 0.77 (0.28, 2.31) | 0.78 (0.31, 2.11) | 0.66 (0.24, 1.91) | 0.90 (0.35, 2.51) | 1.38 (0.38, 5.41) | 0.61 (0.15, 2.48) | 0.80 (0.32, 2.18) | 0.80 (0.32, 2.21) | 0.69 (0.27, 1.87) | 1.01 (0.36, 3.09) | 1.11 (0.42, 3.13) | 1.15 (0.46, 3.11) |
| 0.97 (0.50, 1.84) | 0.68 (0.24, 1.85) | 0.66 (0.32, 1.34) | 0.81 (0.41, 1.60) | 0.96 (0.50, 1.81) | 1.44 (0.53, 3.93) | 0.76 (0.27, 2.11) | 1.11 (0.34, 3.35) | **ARB+**  **CCB** | 1.03 (0.65, 1.62) | 1.13 (0.58, 2.16) | 0.85 (0.39, 1.88) | 0.87 (0.45, 1.64) | 0.74 (0.34, 1.54) | 1.00 (0.49, 1.99) | 1.53 (0.79, 3.08) | 0.68 (0.30, 1.50) | 0.89 (0.46, 1.70) | 0.90 (0.46, 1.74) | 0.77 (0.39, 1.47) | 1.13 (0.48, 2.57) | 1.24 (0.59, 2.56) | 1.28 (0.68, 2.39) |
| 0.94 (0.59, 1.49) | 0.66 (0.25, 1.63) | 0.64 (0.37, 1.11) | 0.78 (0.47, 1.30) | 0.93 (0.58, 1.47) | 1.40 (0.57, 3.45) | 0.74 (0.30, 1.85) | 1.08 (0.37, 2.95) | 0.97 (0.62, 1.53) | **ARB+DI** | 1.10 (0.67, 1.76) | 0.83 (0.43, 1.58) | 0.84 (0.53, 1.33) | 0.72 (0.39, 1.29) | 0.98 (0.57, 1.64) | 1.49 (0.67, 3.40) | 0.65 (0.25, 1.64) | 0.87 (0.53, 1.38) | 0.87 (0.53, 1.41) | 0.74 (0.45, 1.20) | 1.10 (0.54, 2.20) | 1.21 (0.68, 2.10) | 1.24 (0.80, 1.91) |
| 0.86 (0.69, 1.07) | 0.60 (0.26, 1.33) | **0.59 (0.40, 0.87)** | **0.71 (0.52, 1.00)** | 0.84 (0.69, 1.05) | 1.28 (0.59, 2.81) | 0.68 (0.30, 1.52) | 0.99 (0.37, 2.45) | 0.88 (0.46, 1.74) | 0.91 (0.57, 1.48) | **BB** | 0.75 (0.45, 1.28) | **0.77 (0.62, 0.95)** | 0.65 (0.42, 1.01) | 0.89 (0.67, 1.18) | 1.36 (0.54, 3.51) | 0.60 (0.21, 1.69) | **0.79 (0.62, 1.00)** | 0.79 (0.60, 1.06) | **0.68 (0.53, 0.87)** | 1.00 (0.6, 1.66) | 1.10 (0.74, 1.61) | 1.13 (0.93, 1.40) |
| 1.13 (0.68, 1.86) | 0.79 (0.30, 2.00) | 0.78 (0.55, 1.09) | 0.94 (0.59, 1.52) | 1.12 (0.68, 1.84) | 1.70 (0.69, 4.17) | 0.90 (0.35, 2.24) | 1.31 (0.43, 3.60) | 1.17 (0.53, 2.60) | 1.20 (0.63, 2.30) | 1.32 (0.78, 2.21) | **BB**  **+DI** | 1.02 (0.62, 1.65) | 0.86 (0.45, 1.60) | 1.18 (0.66, 2.04) | 1.80 (0.64, 5.08) | 0.79 (0.25, 2.42) | 1.05 (0.62, 1.73) | 1.05 (0.62, 1.77) | 0.90 (0.53, 1.49) | 1.33 (0.63, 2.69) | 1.46 (0.79, 2.62) | 1.50 (0.93, 2.43) |
| 1.12 (0.95, 1.30) | 0.78 (0.34, 1.75) | 0.76 (0.54, 1.08) | 0.93 (0.70, 1.24) | 1.10 (0.94, 1.28) | 1.67 (0.77, 3.64) | 0.88 (0.40, 1.98) | 1.28 (0.47, 3.21) | 1.15 (0.61, 2.2) | 1.19 (0.75, 1.88) | **1.30 (1.05, 1.61)** | 0.98 (0.61, 1.61) | **CCB**  **(DH)** | 0.85 (0.55, 1.28) | 1.16 (0.85, 1.56) | 1.77 (0.71, 4.51) | 0.78 (0.28, 2.16) | 1.03 (0.84, 1.24) | 1.03 (0.83, 1.29) | 0.88 (0.71, 1.09) | 1.30 (0.74, 2.26) | 1.43 (0.99, 2.04) | **1.47 (1.28, 1.70)** |
| 1.31 (0.87, 2.01) | 0.92 (0.36, 2.25) | 0.90 (0.53, 1.55) | 1.09 (0.68, 1.81) | 1.30 (0.85, 2.02) | 1.97 (0.83, 4.75) | 1.04 (0.43, 2.53) | 1.52 (0.52, 4.10) | 1.36 (0.65, 2.92) | 1.40 (0.78, 2.58) | 1.54 (0.99, 2.4) | 1.16 (0.62, 2.21) | 1.18 (0.78, 1.81) | **CCB**  **(D)** | 1.36 (0.85, 2.19) | 2.10 (0.77, 5.80) | 0.92 (0.30, 2.74) | 1.21 (0.84, 1.76) | 1.22 (0.78, 1.94) | 1.04 (0.67, 1.63) | 1.54 (0.78, 3.03) | 1.69 (0.99, 2.89) | **1.73 (1.16, 2.67)** |
| 0.96 (0.71, 1.32) | 0.67 (0.28, 1.57) | 0.66 (0.43, 1.04) | 0.80 (0.55, 1.21) | 0.95 (0.70, 1.31) | 1.44 (0.64, 3.30) | 0.76 (0.33, 1.77) | 1.11 (0.40, 2.87) | 1.00 (0.50, 2.02) | 1.03 (0.61, 1.76) | 1.13 (0.85, 1.50) | 0.85 (0.49, 1.51) | 0.86 (0.64, 1.18) | 0.73 (0.46, 1.18) | **CCB**  **(V)** | 1.53 (0.59, 4.08) | 0.67 (0.23, 1.95) | 0.89 (0.66, 1.19) | 0.89 (0.63, 1.28) | 0.76 (0.54, 1.07) | 1.13 (0.63, 2.02) | 1.24 (0.78, 1.93) | 1.27 (0.95, 1.74) |
| 0.63 (0.25, 1.58) | 0.44 (0.12, 1.46) | 0.43 (0.16, 1.14) | 0.52 (0.20, 1.36) | 0.62 (0.24, 1.57) | 0.94 (0.28, 3.15) | 0.50 (0.15, 1.68) | 0.72 (0.18, 2.63) | 0.65 (0.33, 1.26) | 0.67 (0.29, 1.49) | 0.73 (0.28, 1.86) | 0.56 (0.20, 1.56) | 0.56 (0.22, 1.41) | 0.48 (0.17, 1.29) | 0.65 (0.25, 1.70) | **CCB+BB** | **0.44 (0.20, 0.90)** | 0.58 (0.22, 1.46) | 0.58 (0.23, 1.49) | 0.50 (0.19, 1.25) | 0.74 (0.25, 2.12) | 0.81 (0.30, 2.15) | 0.83 (0.33, 2.07) |
| 1.43 (0.51, 4.07) | 1.00 (0.27, 3.61) | 0.98 (0.34, 2.92) | 1.20 (0.42, 3.49) | 1.42 (0.51, 4.02) | 2.14 (0.61, 7.80) | 1.13 (0.32, 4.17) | 1.64 (0.40, 6.53) | 1.48 (0.67, 3.37) | 1.53 (0.61, 3.94) | 1.68 (0.59, 4.76) | 1.27 (0.41, 3.96) | 1.29 (0.46, 3.63) | 1.09 (0.36, 3.29) | 1.49 (0.51, 4.32) | **2.27 (1.11, 4.91)** | **CCB+DI** | 1.32 (0.47, 3.75) | 1.33 (0.47, 3.82) | 1.14 (0.40, 3.24) | 1.68 (0.52, 5.37) | 1.84 (0.62, 5.44) | 1.89 (0.69, 5.32) |
| 1.08 (0.90, 1.32) | 0.76 (0.32, 1.72) | 0.74 (0.51, 1.10) | 0.90 (0.67, 1.26) | 1.07 (0.87, 1.34) | 1.63 (0.74, 3.60) | 0.86 (0.38, 1.94) | 1.25 (0.46, 3.16) | 1.12 (0.59, 2.18) | 1.15 (0.72, 1.87) | 1.27 (1.00, 1.62) | 0.96 (0.58, 1.61) | 0.97 (0.80, 1.19) | 0.82 (0.57, 1.20) | 1.12 (0.84, 1.51) | 1.73 (0.69, 4.47) | 0.76 (0.27, 2.12) | **CT** | 1.00 (0.78, 1.32) | 0.86 (0.67, 1.10) | 1.27 (0.72, 2.23) | 1.39 (0.94, 2.05) | **1.43 (1.20, 1.74)** |
| 1.08 (0.86, 1.36) | 0.75 (0.32, 1.72) | 0.74 (0.50, 1.10) | 0.90 (0.65, 1.27) | 1.07 (0.83, 1.36) | 1.61 (0.73, 3.62) | 0.85 (0.38, 1.95) | 1.24 (0.45, 3.15) | 1.12 (0.58, 2.18) | 1.15 (0.71, 1.87) | 1.26 (0.95, 1.66) | 0.95 (0.57, 1.61) | 0.97 (0.78, 1.20) | 0.82 (0.51, 1.28) | 1.12 (0.78, 1.58) | 1.72 (0.67, 4.44) | 0.75 (0.26, 2.11) | 1.00 (0.76, 1.29) | **DI**  **(TI)** | 0.85 (0.64, 1.12) | 1.26 (0.70, 2.25) | 1.39 (0.91, 2.07) | **1.43 (1.14, 1.79)** |
| **1.26 (1.02, 1.59)** | 0.88 (0.38, 2.00) | 0.87 (0.59, 1.28) | 1.05 (0.77, 1.48) | 1.25 (0.99, 1.59) | 1.89 (0.86, 4.21) | 1.00 (0.44, 2.28) | 1.46 (0.53, 3.68) | 1.31 (0.68, 2.56) | 1.35 (0.84, 2.20) | **1.48 (1.15, 1.90)** | 1.11 (0.67, 1.89) | 1.13 (0.92, 1.42) | 0.96 (0.61, 1.50) | 1.31 (0.93, 1.84) | 2.01 (0.80, 5.20) | 0.88 (0.31, 2.49) | 1.17 (0.91, 1.50) | 1.17 (0.89, 1.57) | **DI**  **(TT)** | 1.48 (0.84, 2.61) | **1.62 (1.09, 2.42)** | **1.67 (1.37, 2.07)** |
| 0.86 (0.49, 1.50) | 0.60 (0.22, 1.55) | 0.58 (0.31, 1.12) | 0.71 (0.39, 1.33) | 0.85 (0.49, 1.48) | 1.28 (0.50, 3.25) | 0.68 (0.26, 1.77) | 0.99 (0.32, 2.81) | 0.88 (0.39, 2.08) | 0.91 (0.46, 1.85) | 1.00 (0.60, 1.67) | 0.75 (0.37, 1.58) | 0.77 (0.44, 1.34) | 0.65 (0.33, 1.28) | 0.89 (0.49, 1.59) | 1.36 (0.47, 4.05) | 0.59 (0.19, 1.91) | 0.79 (0.45, 1.39) | 0.79 (0.44, 1.43) | 0.68 (0.38, 1.20) | **non**  **BB** | 1.10 (0.58, 2.08) | 1.13 (0.66, 1.98) |
| 0.78 (0.54, 1.13) | 0.54 (0.22, 1.30) | **0.53 (0.33, 0.88)** | 0.65 (0.42, 1.02) | 0.77 (0.56, 1.07) | 1.17 (0.51, 2.72) | 0.62 (0.26, 1.48) | 0.90 (0.32, 2.37) | 0.81 (0.39, 1.69) | 0.83 (0.48, 1.47) | 0.91 (0.62, 1.35) | 0.69 (0.38, 1.26) | 0.70 (0.49, 1.01) | 0.59 (0.35, 1.01) | 0.81 (0.52, 1.27) | 1.24 (0.47, 3.36) | 0.54 (0.18, 1.60) | 0.72 (0.49, 1.06) | 0.72 (0.48, 1.09) | **0.62 (0.41, 0.92)** | 0.91 (0.48, 1.73) | **non**  **RASI** | 1.03 (0.72, 1.50) |
| **0.76 (0.64, 0.89)** | 0.53 (0.23, 1.19) | **0.52 (0.37, 0.72)** | **0.63 (0.49, 0.82)** | **0.75 (0.64, 0.87)** | 1.13 (0.52, 2.47) | 0.60 (0.27, 1.34) | 0.87 (0.32, 2.18) | 0.78 (0.42, 1.46) | 0.81 (0.52, 1.24) | 0.89 (0.71, 1.08) | 0.67 (0.41, 1.08) | **0.68 (0.59, 0.78)** | **0.58 (0.38, 0.86)** | 0.79 (0.57, 1.05) | 1.21 (0.48, 3.03) | 0.53 (0.19, 1.45) | **0.70 (0.57, 0.83)** | **0.70 (0.56, 0.87)** | **0.60 (0.48, 0.73)** | 0.88 (0.51, 1.52) | 0.97 (0.67, 1.38) | **Placebo** |

Abbreviations: CrI, credible interval; ARB, angiotensin receptor blockers; DI, Diuretics; DI(TL), thiazide-like diuretics; DI(TT), thiazide-type diuretics; CCB, calcium channel blockers; CCB(DH), dihydropyridine calcium channel blockers; CCB(D), calcium channel blockers (diltiazem); CCB(V), calcium channel blockers (verapamil); ACEI, angiotensin-converting enzyme inhibitor; BB, β adrenergic receptor blockers; nonRASI, non-renin-angiotensin system (RAS) inhibitors; RI, renin inhibitors.

Effect sizes represent summary relative risk and 95% credible intervals. Bold values indicate significant results. In the upper triangle, values greater than 1 favor the treatment in the corresponding row, whereas values less than 1 favor the treatment in the corresponding column. In the lower triangle, values greater than 1 favor the treatment in the corresponding column, whereas values less than 1 favor the treatment in the corresponding row.
