# Supplementary material for: Comparative efficacy of different antihypertensive drug classes for stroke prevention: A network meta-analysis of randomized controlled trials
Source: PLoS One. 2025 Feb 21;20(2):e0313309. doi: 10.1371/journal.pone.0313309 (PMC11845040; doi:10.1371/journal.pone.0313309)
Supplement: S22 Table — (DOCX) [file pone.0313309.s023.docx]

**S22 Table. Relative risk [RR] with 95% CrI for all-cause mortality of the overall population.**

| **ACEI** | 0.81 (0.56, 1.16) | **0.79 (0.66, 0.94)** | 0.92 (0.82, 1.02) | **1.05 (1.01, 1.11)** | 1.05 (0.97, 1.13) | 1.15 (0.83, 1.60) | 0.53 (0.26, 1.03) | 0.91 (0.63, 1.34) | 1.07 (0.90, 1.28) | **1.08 (1.00, 1.18)** | 0.88 (0.70, 1.09) | 1.02 (0.96, 1.09) | 0.84 (0.45, 1.68) | 0.84 (0.43, 1.72) | 0.97 (0.89, 1.06) | 1.01 (0.94, 1.08) | 1.15 (0.86, 1.52) | 0.99 (0.75, 1.30) | **1.09 (1.05, 1.15)** | 1.15 (0.97, 1.36) |
| --- | --- | --- | --- | --- | --- | --- | --- | --- | --- | --- | --- | --- | --- | --- | --- | --- | --- | --- | --- | --- |
| 1.24 (0.86, 1.79) | **ACEI+BB** | 0.97 (0.65, 1.51) | 1.13 (0.78, 1.67) | 1.30 (0.91, 1.88) | 1.30 (0.91, 1.87) | 1.44 (0.99, 2.08) | 0.66 (0.31, 1.23) | 1.14 (0.67, 1.93) | 1.33 (0.89, 2.00) | 1.34 (0.93, 1.95) | 1.09 (0.71, 1.70) | 1.26 (0.88, 1.83) | 1.06 (0.50, 2.26) | 1.05 (0.49, 2.38) | 1.20 (0.83, 1.76) | 1.25 (0.87, 1.82) | 1.42 (0.89, 2.25) | 1.23 (0.79, 1.95) | 1.36 (0.95, 1.96) | 1.43 (0.96, 2.13) |
| **1.27 (1.07, 1.51)** | 1.03 (0.66, 1.53) | **ACEI+**  **CCB** | 1.16 (0.98, 1.37) | **1.34 (1.12, 1.59)** | **1.33 (1.10, 1.61)** | **1.47 (1.01, 2.11)** | 0.67 (0.33, 1.32) | 1.16 (0.77, 1.76) | **1.36 (1.05, 1.73)** | **1.38 (1.14, 1.66)** | 1.11 (0.98, 1.27) | **1.29 (1.08, 1.54)** | 1.07 (0.56, 2.20) | 1.07 (0.54, 2.19) | **1.23 (1.03, 1.49)** | **1.28 (1.07, 1.53)** | **1.45 (1.06, 2.01)** | 1.25 (0.92, 1.73) | **1.39 (1.17, 1.65)** | **1.46 (1.16, 1.84)** |
| 1.09 (0.98, 1.22) | 0.88 (0.60, 1.28) | 0.86 (0.73, 1.02) | **ACEI+DI** | **1.15 (1.03, 1.28)** | **1.14 (1.01, 1.30)** | 1.26 (0.89, 1.77) | 0.58 (0.28, 1.13) | 1.00 (0.68, 1.48) | 1.17 (0.96, 1.43) | **1.18 (1.05, 1.35)** | 0.96 (0.78, 1.18) | **1.11 (1.00, 1.25)** | 0.92 (0.48, 1.86) | 0.92 (0.47, 1.89) | 1.06 (0.93, 1.21) | 1.10 (0.98, 1.24) | 1.25 (0.93, 1.68) | 1.08 (0.81, 1.44) | **1.19 (1.08, 1.32)** | **1.25 (1.04, 1.52)** |
| **0.95 (0.90, 0.99)** | 0.77 (0.53, 1.10) | **0.75 (0.63, 0.89)** | **0.87 (0.78, 0.97)** | **ARB** | 1.00 (0.92, 1.08) | 1.09 (0.79, 1.52) | **0.50 (0.25, 0.97)** | 0.87 (0.60, 1.27) | 1.02 (0.85, 1.21) | 1.03 (0.95, 1.12) | 0.83 (0.67, 1.03) | 0.97 (0.91, 1.03) | 0.80 (0.42, 1.60) | 0.80 (0.41, 1.64) | 0.92 (0.84, 1.01) | 0.96 (0.89, 1.03) | 1.09 (0.82, 1.44) | 0.94 (0.72, 1.23) | **1.04 (1.00, 1.09)** | 1.09 (0.92, 1.29) |
| 0.95 (0.88, 1.03) | 0.77 (0.53, 1.10) | **0.75 (0.62, 0.91)** | **0.87 (0.77, 0.99)** | 1.00 (0.93, 1.08) | **ARB+**  **ACEI** | 1.10 (0.79, 1.52) | **0.50 (0.25, 0.98)** | 0.87 (0.60, 1.29) | 1.02 (0.85, 1.23) | 1.03 (0.93, 1.15) | 0.84 (0.66, 1.05) | 0.97 (0.89, 1.07) | 0.80 (0.42, 1.61) | 0.80 (0.41, 1.66) | 0.93 (0.83, 1.04) | 0.96 (0.87, 1.06) | 1.09 (0.81, 1.46) | 0.94 (0.71, 1.25) | 1.04 (0.96, 1.13) | 1.10 (0.91, 1.32) |
| 0.87 (0.63, 1.21) | 0.70 (0.48, 1.01) | **0.68 (0.47, 0.99)** | 0.79 (0.57, 1.12) | 0.91 (0.66, 1.27) | 0.91 (0.66, 1.27) | **ARB+**  **ACEI+BB** | **0.47 (0.22, 0.85)** | 0.79 (0.48, 1.30) | 0.93 (0.65, 1.35) | 0.94 (0.68, 1.32) | 0.76 (0.52, 1.12) | 0.89 (0.64, 1.24) | 0.73 (0.36, 1.55) | 0.73 (0.35, 1.61) | 0.84 (0.60, 1.18) | 0.88 (0.63, 1.22) | 0.99 (0.64, 1.52) | 0.86 (0.56, 1.33) | 0.95 (0.69, 1.32) | 1.00 (0.69, 1.44) |
| 1.90 (0.98, 3.81) | 1.51 (0.82, 3.19) | 1.50 (0.76, 3.03) | 1.73 (0.89, 3.53) | **2.00 (1.03, 4.01)** | **1.99 (1.02, 4.00)** | **2.15 (1.17, 4.52)** | **ARB+BB** | 1.74 (0.82, 3.83) | **2.03 (1.03, 4.19)** | **2.06 (1.06, 4.12)** | 1.66 (0.83, 3.41) | 1.93 (0.99, 3.87) | 1.60 (0.66, 4.20) | 1.59 (0.65, 4.35) | 1.84 (0.95, 3.71) | 1.91 (0.98, 3.86) | **2.17 (1.08, 4.64)** | 1.87 (0.91, 4.03) | 2.08 (1.07, 4.18) | 2.18 (1.10, 4.45) |
| 1.09 (0.75, 1.59) | 0.88 (0.52, 1.50) | 0.86 (0.57, 1.29) | 1.00 (0.67, 1.48) | 1.15 (0.78, 1.68) | 1.15 (0.78, 1.68) | 1.26 (0.77, 2.09) | 0.58 (0.26, 1.23) | **ARB+**  **CCB** | 1.17 (0.83, 1.64) | 1.19 (0.80, 1.74) | 0.96 (0.62, 1.46) | 1.11 (0.76, 1.63) | 0.93 (0.54, 1.66) | 0.92 (0.53, 1.64) | 1.06 (0.72, 1.55) | 1.10 (0.75, 1.61) | 1.25 (0.78, 2.01) | 1.08 (0.68, 1.74) | 1.20 (0.82, 1.74) | 1.26 (0.83, 1.89) |
| 0.93 (0.78, 1.11) | 0.75 (0.50, 1.13) | **0.73 (0.58, 0.95)** | 0.85 (0.70, 1.04) | 0.98 (0.82, 1.17) | 0.98 (0.81, 1.18) | 1.08 (0.74, 1.55) | **0.49 (0.24, 0.97)** | 0.85 (0.61, 1.21) | **ARB+DI** | 1.01 (0.84, 1.22) | 0.82 (0.62, 1.08) | 0.95 (0.80, 1.14) | 0.79 (0.42, 1.53) | 0.79 (0.41, 1.59) | 0.91 (0.75, 1.10) | 0.94 (0.78, 1.13) | 1.07 (0.77, 1.48) | 0.92 (0.67, 1.27) | 1.02 (0.86, 1.21) | 1.07 (0.85, 1.36) |
| **0.92 (0.85, 1.00)** | 0.74 (0.51, 1.07) | **0.73 (0.60, 0.87)** | **0.85 (0.74, 0.95)** | 0.97 (0.89, 1.05) | 0.97 (0.87, 1.07) | 1.06 (0.76, 1.48) | **0.48 (0.24, 0.94)** | 0.84 (0.57, 1.25) | 0.99 (0.82, 1.19) | **BB** | 0.81 (0.64, 1.01) | 0.94 (0.87, 1.02) | 0.78 (0.41, 1.56) | 0.78 (0.40, 1.60) | **0.90 (0.80, 1.00)** | 0.93 (0.85, 1.02) | 1.06 (0.80, 1.39) | 0.91 (0.69, 1.21) | 1.01 (0.93, 1.09) | 1.06 (0.88, 1.27) |
| 1.14 (0.92, 1.42) | 0.92 (0.59, 1.40) | 0.90 (0.79, 1.03) | 1.05 (0.85, 1.29) | 1.20 (0.97, 1.49) | 1.20 (0.95, 1.51) | 1.32 (0.89, 1.94) | 0.60 (0.29, 1.20) | 1.04 (0.68, 1.60) | 1.22 (0.92, 1.61) | 1.24 (0.99, 1.56) | **BB+**  **DI** | 1.16 (0.94, 1.45) | 0.96 (0.49, 1.99) | 0.96 (0.48, 1.99) | 1.11 (0.88, 1.40) | 1.15 (0.92, 1.44) | 1.30 (0.92, 1.86) | 1.12 (0.80, 1.60) | **1.25 (1.01, 1.55)** | **1.31 (1.01, 1.72)** |
| 0.98 (0.92, 1.04) | 0.79 (0.55, 1.14) | **0.77 (0.65, 0.92)** | **0.90 (0.80, 1.00)** | 1.03 (0.97, 1.10) | 1.03 (0.94, 1.13) | 1.13 (0.81, 1.57) | 0.52 (0.26, 1.01) | 0.90 (0.61, 1.32) | 1.05 (0.88, 1.26) | 1.07 (0.99, 1.15) | 0.86 (0.69, 1.07) | **CCB** | 0.83 (0.43, 1.66) | 0.83 (0.42, 1.71) | 0.95 (0.88, 1.04) | 0.99 (0.92, 1.06) | 1.13 (0.84, 1.49) | 0.97 (0.74, 1.28) | **1.08 (1.01, 1.14)** | 1.13 (0.95, 1.34) |
| 1.19 (0.59, 2.25) | 0.94 (0.44, 1.99) | 0.93 (0.46, 1.80) | 1.08 (0.54, 2.08) | 1.25 (0.63, 2.37) | 1.24 (0.62, 2.37) | 1.37 (0.64, 2.81) | 0.62 (0.24, 1.52) | 1.08 (0.60, 1.84) | 1.27 (0.65, 2.36) | 1.29 (0.64, 2.46) | 1.04 (0.50, 2.03) | 1.21 (0.60, 2.30) | **CCB+BB** | 0.99 (0.57, 1.82) | 1.15 (0.57, 2.19) | 1.20 (0.60, 2.28) | 1.35 (0.65, 2.75) | 1.17 (0.57, 2.35) | 1.30 (0.65, 2.46) | 1.36 (0.67, 2.63) |
| 1.18 (0.58, 2.31) | 0.95 (0.42, 2.04) | 0.93 (0.46, 1.84) | 1.09 (0.53, 2.13) | 1.25 (0.61, 2.44) | 1.24 (0.60, 2.43) | 1.37 (0.62, 2.88) | 0.63 (0.23, 1.53) | 1.08 (0.61, 1.89) | 1.27 (0.63, 2.44) | 1.29 (0.62, 2.52) | 1.04 (0.50, 2.07) | 1.21 (0.58, 2.36) | 1.01 (0.55, 1.76) | **CCB+DI** | 1.15 (0.56, 2.26) | 1.20 (0.59, 2.34) | 1.35 (0.64, 2.85) | 1.16 (0.56, 2.46) | 1.30 (0.64, 2.53) | 1.36 (0.66, 2.72) |
| 1.03 (0.94, 1.13) | 0.83 (0.57, 1.21) | 0.81 (0.67, 0.97) | 0.94 (0.82, 1.07) | 1.08 (0.99, 1.19) | 1.08 (0.96, 1.21) | 1.19 (0.85, 1.66) | 0.54 (0.27, 1.06) | 0.94 (0.64, 1.39) | 1.10 (0.91, 1.34) | **1.12 (1.00, 1.25)** | 0.90 (0.72, 1.13) | 1.05 (0.96, 1.14) | 0.87 (0.46, 1.74) | 0.87 (0.44, 1.78) | **CT** | 1.04 (0.94, 1.15) | 1.18 (0.88, 1.57) | 1.02 (0.77, 1.36) | **1.13 (1.03, 1.23)** | 1.18 (0.98, 1.43) |
| 0.99 (0.93, 1.06) | 0.80 (0.55, 1.15) | **0.78 (0.65, 0.93)** | 0.91 (0.81, 1.02) | 1.04 (0.97, 1.12) | 1.04 (0.94, 1.15) | 1.14 (0.82, 1.59) | 0.52 (0.26, 1.02) | 0.91 (0.62, 1.34) | 1.06 (0.89, 1.28) | 1.07 (0.98, 1.18) | 0.87 (0.70, 1.08) | 1.01 (0.94, 1.09) | 0.84 (0.44, 1.67) | 0.84 (0.43, 1.70) | 0.96 (0.87, 1.07) | **DI** | 1.13 (0.85, 1.51) | 0.98 (0.74, 1.30) | **1.08 (1.01, 1.16)** | 1.14 (0.95, 1.36) |
| 0.87 (0.66, 1.16) | 0.70 (0.45, 1.12) | **0.69 (0.50, 0.94)** | 0.80 (0.59, 1.08) | 0.92 (0.69, 1.22) | 0.92 (0.69, 1.23) | 1.01 (0.66, 1.55) | **0.46 (0.22, 0.93)** | 0.80 (0.50, 1.28) | 0.94 (0.68, 1.31) | 0.95 (0.72, 1.25) | 0.77 (0.54, 1.08) | 0.89 (0.67, 1.19) | 0.74 (0.36, 1.55) | 0.74 (0.35, 1.56) | 0.85 (0.64, 1.14) | 0.88 (0.66, 1.18) | **non**  **BB** | 0.86 (0.59, 1.27) | 0.95 (0.72, 1.27) | 1.00 (0.73, 1.39) |
| 1.01 (0.77, 1.33) | 0.82 (0.51, 1.27) | 0.80 (0.58, 1.08) | 0.93 (0.69, 1.23) | 1.07 (0.81, 1.40) | 1.06 (0.80, 1.40) | 1.17 (0.75, 1.79) | 0.53 (0.25, 1.10) | 0.93 (0.58, 1.46) | 1.08 (0.79, 1.50) | 1.10 (0.83, 1.46) | 0.89 (0.63, 1.24) | 1.03 (0.78, 1.35) | 0.86 (0.42, 1.77) | 0.86 (0.41, 1.78) | 0.98 (0.73, 1.31) | 1.02 (0.77, 1.35) | 1.16 (0.79, 1.71) | **non**  **RASI** | 1.11 (0.84, 1.45) | 1.16 (0.84, 1.60) |
| **0.91 (0.87, 0.95)** | 0.74 (0.51, 1.06) | **0.72 (0.61, 0.85)** | **0.84 (0.76, 0.92)** | **0.96 (0.92, 1.00)** | 0.96 (0.88, 1.04) | 1.05 (0.76, 1.46) | **0.48 (0.24, 0.94)** | 0.84 (0.57, 1.22) | 0.98 (0.82, 1.16) | 0.99 (0.92, 1.07) | **0.80 (0.65, 0.99)** | **0.93 (0.88, 0.99)** | 0.77 (0.41, 1.53) | 0.77 (0.40, 1.57) | **0.89 (0.81, 0.97)** | **0.92 (0.86, 0.99)** | 1.05 (0.79, 1.38) | 0.90 (0.69, 1.19) | **Placebo** | 1.05 (0.89, 1.24) |
| 0.87 (0.73, 1.03) | 0.70 (0.47, 1.04) | **0.69 (0.54, 0.87)** | **0.80 (0.66, 0.96)** | 0.92 (0.77, 1.09) | 0.91 (0.76, 1.10) | 1.00 (0.69, 1.45) | **0.46 (0.22, 0.91)** | 0.80 (0.53, 1.20) | 0.93 (0.73, 1.18) | 0.94 (0.79, 1.13) | **0.76 (0.58, 0.99)** | 0.89 (0.74, 1.06) | 0.73 (0.38, 1.48) | 0.74 (0.37, 1.51) | 0.85 (0.70, 1.02) | 0.88 (0.73, 1.05) | 1.00 (0.72, 1.38) | 0.86 (0.63, 1.19) | 0.95 (0.81, 1.12) | **RI** |

Abbreviations: CrI, credible interval; ARB, angiotensin receptor blockers; DI, Diuretics; DI(TL), thiazide-like diuretics; DI(TT), thiazide-type diuretics; CCB, calcium channel blockers; CCB(DH), dihydropyridine calcium channel blockers; CCB(D), calcium channel blockers (diltiazem); CCB(V), calcium channel blockers (verapamil); ACEI, angiotensin-converting enzyme inhibitor; BB, β adrenergic receptor blockers; nonRASI, non-renin-angiotensin system (RAS) inhibitors; RI, renin inhibitors.

Effect sizes represent summary relative risk and 95% credible intervals. Bold values indicate significant results. In the upper triangle, values greater than 1 favor the treatment in the corresponding row, whereas values less than 1 favor the treatment in the corresponding column. In the lower triangle, values greater than 1 favor the treatment in the corresponding column, whereas values less than 1 favor the treatment in the corresponding row.
