# Supplementary material for: Comparative efficacy of different antihypertensive drug classes for stroke prevention: A network meta-analysis of randomized controlled trials
Source: PLoS One. 2025 Feb 21;20(2):e0313309. doi: 10.1371/journal.pone.0313309 (PMC11845040; doi:10.1371/journal.pone.0313309)
Supplement: S24 Table — (DOCX) [file pone.0313309.s025.docx]

**S24 Table. Relative risk [RR] with 95% CrI for subgroup analysis of all-cause mortality among hypertensive patients.**

| **ACEI** | 0.86 (0.58, 1.31) | **0.78 (0.64, 0.94)** | 0.92 (0.79, 1.08) | 1.05 (0.94, 1.17) | 1.14 (0.73, 1.75) | 1.25 (0.86, 1.82) | 0.61 (0.28, 1.10) | 0.95 (0.63, 1.40) | 1.13 (0.93, 1.39) | **1.18 (1.05, 1.33)** | 0.87 (0.68, 1.10) | 1.00 (0.93, 1.11) | 1.03 (0.81, 1.31) | 1.13 (0.98, 1.32) | 0.89 (0.45, 1.71) | 0.88 (0.45, 1.76) | 1.01 (0.90, 1.13) | 1.01 (0.91, 1.11) | 1.05 (0.94, 1.19) | 1.26 (0.93, 1.72) | 1.00 (0.76, 1.34) | **1.16 (1.06, 1.27)** |
| --- | --- | --- | --- | --- | --- | --- | --- | --- | --- | --- | --- | --- | --- | --- | --- | --- | --- | --- | --- | --- | --- | --- |
| 1.17 (0.76, 1.74) | **ACEI+BB** | 0.91 (0.56, 1.40) | 1.08 (0.68, 1.65) | 1.23 (0.80, 1.85) | 1.32 (0.86, 2.01) | 1.45 (0.98, 2.11) | 0.71 (0.33, 1.30) | 1.11 (0.62, 1.92) | 1.33 (0.83, 2.05) | 1.38 (0.91, 2.07) | 1.01 (0.62, 1.60) | 1.18 (0.77, 1.77) | 1.21 (0.75, 1.88) | 1.33 (0.86, 2.00) | 1.03 (0.49, 2.18) | 1.05 (0.48, 2.21) | 1.18 (0.76, 1.78) | 1.17 (0.76, 1.77) | 1.23 (0.81, 1.86) | 1.48 (0.89, 2.39) | 1.18 (0.73, 1.91) | 1.35 (0.88, 2.03) |
| **1.28 (1.06, 1.57)** | 1.09 (0.71, 1.79) | **ACEI+**  **CCB** | **1.18 (1.00, 1.41)** | **1.34 (1.11, 1.65)** | 1.46 (0.91, 2.34) | **1.61 (1.04, 2.41)** | 0.78 (0.36, 1.45) | 1.21 (0.78, 1.86) | **1.44 (1.13, 1.89)** | **1.51 (1.24, 1.88)** | 1.11 (0.96, 1.29) | **1.28 (1.08, 1.57)** | 1.31 (0.99, 1.79) | **1.45 (1.17, 1.84)** | 1.14 (0.57, 2.22) | 1.13 (0.57, 2.32) | **1.28 (1.06, 1.61)** | **1.28 (1.05, 1.58)** | **1.35 (1.10, 1.68)** | **1.61 (1.13, 2.30)** | 1.29 (0.94, 1.81) | **1.48 (1.24, 1.80)** |
| 1.08 (0.92, 1.27) | 0.93 (0.61, 1.47) | **0.85 (0.71, 1.00)** | **ACEI+DI** | 1.14 (0.97, 1.35) | 1.24 (0.76, 1.94) | 1.37 (0.90, 2.02) | 0.66 (0.30, 1.21) | 1.03 (0.67, 1.54) | 1.23 (0.98, 1.55) | **1.28 (1.09, 1.54)** | 0.94 (0.75, 1.18) | 1.09 (0.94, 1.28) | 1.11 (0.85, 1.48) | **1.23 (1.02, 1.50)** | 0.96 (0.49, 1.87) | 0.95 (0.49, 1.94) | 1.09 (0.92, 1.31) | 1.09 (0.91, 1.29) | 1.14 (0.97, 1.37) | 1.36 (0.99, 1.92) | 1.09 (0.80, 1.50) | **1.25 (1.10, 1.45)** |
| 0.95 (0.85, 1.06) | 0.81 (0.54, 1.25) | **0.74 (0.60, 0.90)** | 0.88 (0.74, 1.03) | **ARB** | 1.08 (0.69, 1.67) | 1.19 (0.80, 1.77) | 0.58 (0.27, 1.06) | 0.91 (0.60, 1.34) | 1.08 (0.88, 1.33) | **1.13 (1.01, 1.26)** | 0.83 (0.64, 1.05) | 0.95 (0.88, 1.06) | 0.98 (0.77, 1.25) | 1.08 (0.93, 1.25) | 0.85 (0.43, 1.62) | 0.84 (0.43, 1.68) | 0.96 (0.85, 1.09) | 0.96 (0.84, 1.08) | 1.00 (0.88, 1.14) | 1.20 (0.89, 1.62) | 0.95 (0.73, 1.26) | **1.10 (1.01, 1.21)** |
| 0.88 (0.57, 1.37) | 0.76 (0.50, 1.17) | 0.69 (0.43, 1.09) | 0.81 (0.51, 1.31) | 0.93 (0.60, 1.45) | **ARB+**  **ACEI** | 1.09 (0.74, 1.69) | 0.53 (0.26, 1.01) | 0.83 (0.47, 1.52) | 0.99 (0.62, 1.63) | 1.04 (0.68, 1.62) | 0.77 (0.46, 1.25) | 0.89 (0.58, 1.39) | 0.91 (0.57, 1.50) | 1.00 (0.64, 1.58) | 0.77 (0.36, 1.69) | 0.78 (0.35, 1.73) | 0.89 (0.57, 1.40) | 0.88 (0.57, 1.39) | 0.92 (0.60, 1.47) | 1.12 (0.67, 1.86) | 0.89 (0.55, 1.51) | 1.02 (0.66, 1.61) |
| 0.80 (0.55, 1.17) | 0.69 (0.47, 1.02) | **0.62 (0.41, 0.96)** | 0.73 (0.5, 1.11) | 0.84 (0.57, 1.24) | 0.91 (0.59, 1.35) | **ARB+**  **ACEI+BB** | **0.49 (0.24, 0.86)** | 0.75 (0.43, 1.32) | 0.90 (0.60, 1.40) | 0.94 (0.64, 1.39) | 0.69 (0.45, 1.09) | 0.80 (0.55, 1.19) | 0.82 (0.53, 1.29) | 0.90 (0.61, 1.35) | 0.70 (0.34, 1.48) | 0.70 (0.31, 1.49) | 0.80 (0.54, 1.20) | 0.80 (0.54, 1.19) | 0.84 (0.58, 1.25) | 1.01 (0.64, 1.61) | 0.80 (0.52, 1.32) | 0.92 (0.63, 1.36) |
| 1.64 (0.91, 3.54) | 1.41 (0.77, 3.00) | 1.28 (0.69, 2.77) | 1.51 (0.83, 3.38) | 1.72 (0.95, 3.77) | 1.87 (0.99, 3.86) | **2.05 (1.16, 4.20)** | **ARB+BB** | 1.56 (0.76, 3.74) | 1.86 (0.99, 4.09) | **1.94 (1.07, 4.19)** | 1.43 (0.75, 3.13) | 1.65 (0.92, 3.57) | 1.69 (0.90, 3.78) | **1.86 (1.02, 4.06)** | 1.45 (0.63, 3.93) | 1.48 (0.60, 4.12) | 1.65 (0.91, 3.61) | 1.65 (0.91, 3.56) | 1.73 (0.96, 3.80) | **2.10 (1.02, 4.54)** | 1.68 (0.85, 3.76) | **1.89 (1.05, 4.19)** |
| 1.05 (0.71, 1.59) | 0.90 (0.52, 1.62) | 0.82 (0.54, 1.28) | 0.97 (0.65, 1.49) | 1.10 (0.75, 1.67) | 1.21 (0.66, 2.13) | 1.33 (0.76, 2.30) | 0.64 (0.27, 1.32) | **ARB+**  **CCB** | 1.19 (0.85, 1.70) | 1.24 (0.83, 1.89) | 0.91 (0.58, 1.45) | 1.05 (0.72, 1.60) | 1.08 (0.69, 1.75) | 1.19 (0.79, 1.82) | 0.93 (0.54, 1.59) | 0.93 (0.51, 1.65) | 1.06 (0.72, 1.61) | 1.06 (0.71, 1.61) | 1.11 (0.75, 1.69) | 1.31 (0.83, 2.18) | 1.06 (0.66, 1.75) | 1.22 (0.83, 1.82) |
| 0.88 (0.72, 1.08) | 0.75 (0.49, 1.20) | **0.69 (0.53, 0.89)** | 0.81 (0.65, 1.02) | 0.93 (0.75, 1.13) | 1.01 (0.61, 1.60) | 1.11 (0.71, 1.67) | 0.54 (0.24, 1.01) | 0.84 (0.59, 1.17) | **ARB+DI** | 1.05 (0.85, 1.29) | 0.77 (0.57, 1.02) | 0.89 (0.73, 1.09) | 0.91 (0.67, 1.22) | 1.00 (0.80, 1.25) | 0.79 (0.41, 1.47) | 0.79 (0.40, 1.54) | 0.89 (0.72, 1.09) | 0.89 (0.71, 1.09) | 0.93 (0.75, 1.15) | 1.12 (0.79, 1.58) | 0.89 (0.64, 1.24) | 1.02 (0.85, 1.22) |
| **0.84 (0.75, 0.95)** | 0.72 (0.48, 1.10) | **0.66 (0.53, 0.81)** | **0.78 (0.65, 0.92)** | **0.89 (0.79, 0.99)** | 0.96 (0.62, 1.47) | 1.06 (0.72, 1.55) | **0.51 (0.24, 0.93)** | 0.81 (0.53, 1.20) | 0.96 (0.78, 1.18) | **BB** | **0.73 (0.56, 0.94)** | **0.85 (0.76, 0.96)** | 0.87 (0.68, 1.11) | 0.96 (0.85, 1.08) | 0.75 (0.38, 1.44) | 0.75 (0.38, 1.50) | **0.85 (0.75, 0.97)** | **0.85 (0.74, 0.97)** | 0.89 (0.79, 1.01) | 1.07 (0.80, 1.41) | 0.85 (0.64, 1.14) | 0.98 (0.88, 1.08) |
| 1.15 (0.91, 1.47) | 0.99 (0.62, 1.63) | 0.90 (0.78, 1.04) | 1.06 (0.85, 1.34) | 1.21 (0.95, 1.55) | 1.30 (0.80, 2.15) | 1.45 (0.92, 2.23) | 0.70 (0.32, 1.33) | 1.10 (0.69, 1.71) | 1.30 (0.98, 1.77) | **1.36 (1.07, 1.77)** | **BB+**  **DI** | 1.16 (0.92, 1.49) | 1.19 (0.86, 1.67) | **1.30 (1.00, 1.72)** | 1.03 (0.50, 2.03) | 1.01 (0.51, 2.11) | 1.16 (0.91, 1.50) | 1.16 (0.90, 1.49) | 1.22 (0.95, 1.57) | 1.45 (0.99, 2.12) | 1.16 (0.82, 1.68) | **1.33 (1.06, 1.70)** |
| 1.00 (0.90, 1.07) | 0.85 (0.57, 1.31) | **0.78 (0.64, 0.93)** | 0.92 (0.78, 1.07) | 1.05 (0.94, 1.14) | 1.13 (0.72, 1.74) | 1.24 (0.84, 1.82) | 0.60 (0.28, 1.09) | 0.95 (0.62, 1.39) | 1.13 (0.92, 1.37) | **1.18 (1.05, 1.31)** | 0.86 (0.67, 1.08) | **CCB**  **(DH)** | 1.03 (0.80, 1.29) | 1.13 (0.97, 1.29) | 0.89 (0.45, 1.68) | 0.88 (0.44, 1.75) | 1.00 (0.89, 1.11) | 1.00 (0.89, 1.09) | 1.05 (0.93, 1.17) | 1.25 (0.93, 1.70) | 1.00 (0.75, 1.32) | **1.15 (1.05, 1.25)** |
| 0.97 (0.77, 1.23) | 0.83 (0.53, 1.33) | 0.76 (0.56, 1.01) | 0.90 (0.68, 1.17) | 1.02 (0.80, 1.30) | 1.10 (0.67, 1.77) | 1.21 (0.78, 1.89) | 0.59 (0.26, 1.12) | 0.93 (0.57, 1.45) | 1.10 (0.82, 1.49) | 1.15 (0.90, 1.47) | 0.84 (0.60, 1.17) | 0.97 (0.77, 1.24) | **CCB**  **(D)** | 1.10 (0.86, 1.41) | 0.87 (0.42, 1.70) | 0.86 (0.41, 1.76) | 0.98 (0.80, 1.21) | 0.98 (0.76, 1.25) | 1.02 (0.80, 1.32) | 1.23 (0.83, 1.76) | 0.97 (0.69, 1.40) | 1.12 (0.89, 1.43) |
| 0.88 (0.76, 1.02) | 0.75 (0.50, 1.16) | **0.69 (0.54, 0.86)** | **0.82 (0.67, 0.99)** | 0.93 (0.80, 1.07) | 1.00 (0.63, 1.56) | 1.11 (0.74, 1.64) | **0.54 (0.25, 0.98)** | 0.84 (0.55, 1.26) | 1.00 (0.80, 1.26) | 1.05 (0.93, 1.18) | **0.77 (0.58, 1.00)** | 0.89 (0.77, 1.03) | 0.91 (0.71, 1.16) | **CCB**  **(V)** | 0.79 (0.40, 1.51) | 0.78 (0.39, 1.57) | 0.89 (0.78, 1.02) | 0.89 (0.75, 1.04) | 0.93 (0.79, 1.09) | 1.11 (0.81, 1.51) | 0.88 (0.66, 1.21) | 1.02 (0.89, 1.17) |
| 1.12 (0.59, 2.20) | 0.97 (0.46, 2.06) | 0.87 (0.45, 1.76) | 1.04 (0.54, 2.06) | 1.18 (0.62, 2.31) | 1.30 (0.59, 2.75) | 1.42 (0.68, 2.91) | 0.69 (0.25, 1.59) | 1.07 (0.63, 1.86) | 1.27 (0.68, 2.42) | 1.33 (0.69, 2.61) | 0.97 (0.49, 1.98) | 1.13 (0.59, 2.22) | 1.15 (0.59, 2.40) | 1.27 (0.66, 2.52) | **CCB+BB** | 1.00 (0.58, 1.79) | 1.13 (0.59, 2.25) | 1.12 (0.59, 2.22) | 1.19 (0.62, 2.34) | 1.43 (0.71, 2.92) | 1.14 (0.57, 2.33) | 1.30 (0.68, 2.54) |
| 1.13 (0.57, 2.24) | 0.95 (0.45, 2.10) | 0.89 (0.43, 1.77) | 1.05 (0.51, 2.06) | 1.20 (0.59, 2.35) | 1.27 (0.58, 2.84) | 1.42 (0.67, 3.23) | 0.68 (0.24, 1.66) | 1.08 (0.61, 1.96) | 1.27 (0.65, 2.49) | 1.34 (0.67, 2.66) | 0.99 (0.47, 1.98) | 1.14 (0.57, 2.26) | 1.16 (0.57, 2.42) | 1.28 (0.64, 2.56) | 1.00 (0.56, 1.73) | **CCB+DI** | 1.14 (0.57, 2.29) | 1.14 (0.57, 2.26) | 1.19 (0.59, 2.37) | 1.42 (0.67, 2.96) | 1.13 (0.55, 2.36) | 1.32 (0.66, 2.59) |
| 0.99 (0.88, 1.11) | 0.85 (0.56, 1.31) | **0.78 (0.62, 0.94)** | 0.92 (0.77, 1.08) | 1.05 (0.92, 1.18) | 1.13 (0.71, 1.75) | 1.25 (0.83, 1.84) | 0.61 (0.28, 1.10) | 0.95 (0.62, 1.40) | 1.12 (0.91, 1.39) | **1.18 (1.03, 1.33)** | 0.86 (0.66, 1.10) | 1.00 (0.90, 1.12) | 1.02 (0.83, 1.26) | 1.13 (0.98, 1.28) | 0.89 (0.45, 1.69) | 0.88 (0.44, 1.75) | **CT** | 1.00 (0.87, 1.14) | 1.05 (0.91, 1.20) | 1.26 (0.91, 1.70) | 1.00 (0.75, 1.34) | **1.15 (1.04, 1.28)** |
| 0.99 (0.90, 1.10) | 0.85 (0.57, 1.31) | **0.78 (0.63, 0.95)** | 0.92 (0.77, 1.09) | 1.05 (0.93, 1.19) | 1.13 (0.72, 1.75) | 1.25 (0.84, 1.83) | 0.61 (0.28, 1.10) | 0.95 (0.62, 1.41) | 1.13 (0.92, 1.41) | **1.18 (1.03, 1.36)** | 0.86 (0.67, 1.11) | 1.00 (0.92, 1.12) | 1.03 (0.80, 1.32) | 1.13 (0.96, 1.33) | 0.89 (0.45, 1.70) | 0.88 (0.44, 1.75) | 1.00 (0.88, 1.15) | **DI**  **(TL)** | 1.05 (0.92, 1.21) | 1.25 (0.92, 1.71) | 1.00 (0.75, 1.35) | **1.15 (1.04, 1.30)** |
| 0.95 (0.84, 1.06) | 0.81 (0.54, 1.24) | **0.74 (0.60, 0.91)** | 0.88 (0.73, 1.03) | 1.00 (0.88, 1.13) | 1.08 (0.68, 1.66) | 1.19 (0.80, 1.73) | 0.58 (0.26, 1.05) | 0.90 (0.59, 1.34) | 1.07 (0.87, 1.33) | 1.12 (0.99, 1.27) | 0.82 (0.63, 1.05) | 0.95 (0.86, 1.07) | 0.98 (0.76, 1.25) | 1.07 (0.91, 1.27) | 0.84 (0.43, 1.62) | 0.84 (0.42, 1.68) | 0.95 (0.83, 1.10) | 0.95 (0.83, 1.09) | **DI**  **(TT)** | 1.19 (0.88, 1.61) | 0.95 (0.71, 1.28) | 1.10 (0.99, 1.22) |
| 0.80 (0.58, 1.07) | 0.68 (0.42, 1.13) | **0.62 (0.44, 0.88)** | 0.73 (0.52, 1.01) | 0.83 (0.62, 1.12) | 0.90 (0.54, 1.49) | 0.99 (0.62, 1.57) | **0.48 (0.22, 0.98)** | 0.76 (0.46, 1.21) | 0.89 (0.63, 1.27) | 0.94 (0.71, 1.25) | 0.69 (0.47, 1.01) | 0.80 (0.59, 1.07) | 0.82 (0.57, 1.20) | 0.90 (0.66, 1.24) | 0.70 (0.34, 1.42) | 0.70 (0.34, 1.50) | 0.79 (0.59, 1.10) | 0.80 (0.58, 1.08) | 0.84 (0.62, 1.13) | **nonBB** | 0.79 (0.54, 1.19) | 0.92 (0.68, 1.24) |
| 1.00 (0.75, 1.31) | 0.85 (0.52, 1.37) | 0.78 (0.55, 1.06) | 0.92 (0.67, 1.25) | 1.05 (0.79, 1.37) | 1.12 (0.66, 1.83) | 1.24 (0.76, 1.93) | 0.59 (0.27, 1.18) | 0.95 (0.57, 1.52) | 1.12 (0.81, 1.57) | 1.18 (0.88, 1.56) | 0.86 (0.60, 1.22) | 1.00 (0.76, 1.33) | 1.03 (0.72, 1.46) | 1.13 (0.83, 1.51) | 0.88 (0.43, 1.76) | 0.89 (0.42, 1.83) | 1.00 (0.74, 1.33) | 1.00 (0.74, 1.33) | 1.05 (0.78, 1.40) | 1.26 (0.84, 1.85) | **nonRASI** | 1.15 (0.87, 1.52) |
| **0.86 (0.79, 0.94)** | 0.74 (0.49, 1.13) | **0.68 (0.56, 0.80)** | **0.80 (0.69, 0.91)** | **0.91 (0.82, 0.99)** | 0.98 (0.62, 1.51) | 1.08 (0.73, 1.60) | **0.53 (0.24, 0.95)** | 0.82 (0.55, 1.20) | 0.98 (0.82, 1.17) | 1.02 (0.92, 1.13) | **0.75 (0.59, 0.94)** | **0.87 (0.80, 0.95)** | 0.89 (0.70, 1.12) | 0.98 (0.85, 1.12) | 0.77 (0.39, 1.46) | 0.76 (0.39, 1.52) | **0.87 (0.78, 0.97)** | **0.87 (0.77, 0.96)** | 0.91 (0.82, 1.01) | 1.09 (0.81, 1.47) | 0.87 (0.66, 1.16) | **Placebo** |

Abbreviations: CrI, credible interval; ARB, angiotensin receptor blockers; DI, Diuretics; DI(TL), thiazide-like diuretics; DI(TT), thiazide-type diuretics; CCB, calcium channel blockers; CCB(DH), dihydropyridine calcium channel blockers; CCB(D), calcium channel blockers (diltiazem); CCB(V), calcium channel blockers (verapamil); ACEI, angiotensin-converting enzyme inhibitor; BB, β adrenergic receptor blockers; nonRASI, non-renin-angiotensin system (RAS) inhibitors; RI, renin inhibitors.

Effect sizes represent summary relative risk and 95% credible intervals. Bold values indicate significant results. In the upper triangle, values greater than 1 favor the treatment in the corresponding row, whereas values less than 1 favor the treatment in the corresponding column. In the lower triangle, values greater than 1 favor the treatment in the corresponding column, whereas values less than 1 favor the treatment in the corresponding row.
