# Supplementary material for: Comparative efficacy of different antihypertensive drug classes for stroke prevention: A network meta-analysis of randomized controlled trials
Source: PLoS One. 2025 Feb 21;20(2):e0313309. doi: 10.1371/journal.pone.0313309 (PMC11845040; doi:10.1371/journal.pone.0313309)
Supplement: S2 Fig — (DOCX) [file pone.0313309.s033.docx]

**S2 Fig. Risk of bias graph for RCTs**

(Review authors' judgement about each risk of bias item presented as percentages across all included RCTs).
