# Supplementary material for: Comparative efficacy of different antihypertensive drug classes for stroke prevention: A network meta-analysis of randomized controlled trials
Source: PLoS One. 2025 Feb 21;20(2):e0313309. doi: 10.1371/journal.pone.0313309 (PMC11845040; doi:10.1371/journal.pone.0313309)
Supplement: S3 Fig — (DOCX) [file pone.0313309.s034.docx]

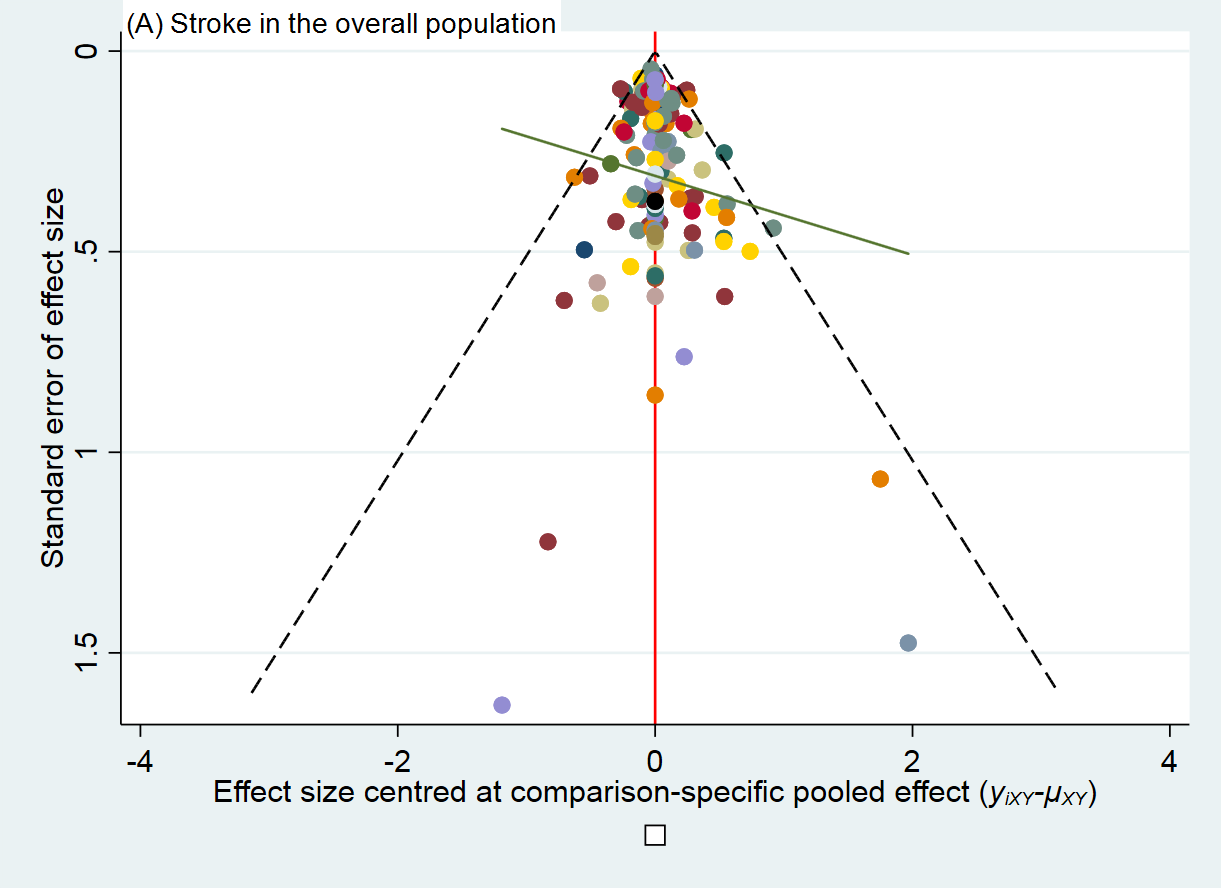


**S3 Fig.** Comparison-adjusted funnel plots regarding the outcome of stroke in the overall population.
